# Evaluating normalization accounts against the dense vowel space of Central Swedish

**DOI:** 10.3389/fpsyg.2023.1165742

**Published:** 2023-06-21

**Authors:** Anna Persson, T. Florian Jaeger

**Affiliations:** ^1^Department of Swedish Language and Multilingualism, Stockholm University, Stockholm, Sweden; ^2^Brain and Cognitive Sciences, University of Rochester, Rochester, NY, United States; ^3^Computer Science, University of Rochester, Rochester, NY, United States

**Keywords:** vowel normalization, ideal observers, speech production, speech perception, category separability

## Abstract

Talkers vary in the phonetic realization of their vowels. One influential hypothesis holds that listeners overcome this inter-talker variability through pre-linguistic auditory mechanisms that normalize the acoustic or phonetic cues that form the input to speech recognition. Dozens of competing normalization accounts exist—including both accounts specific to vowel perception and general purpose accounts that can be applied to any type of cue. We add to the cross-linguistic literature on this matter by comparing normalization accounts against a new phonetically annotated vowel database of Swedish, a language with a particularly dense vowel inventory of 21 vowels differing in quality and quantity. We evaluate normalization accounts on how they differ in predicted consequences for perception. The results indicate that the best performing accounts either center or standardize formants by talker. The study also suggests that general purpose accounts perform as well as vowel-specific accounts, and that vowel normalization operates in both temporal and spectral domains.

## 1. Introduction

Talkers differ in their pronunciation of individual speech sounds due to both physiological differences and socio-cultural factors, including style, regional dialect, and second language accents. For listeners, this means that the mapping from acoustic cues to linguistic categories—phonemes, syllables, words, and ultimately word meanings—varies depending on the talker. How listeners manage to typically understand talkers despite this “lack of invariance” (Liberman et al., 1967) has remained one of the central questions for research on speech perception. Hypotheses about the mechanisms underlying this ability can be grouped into three, mutually compatible and complementary, accounts: (1) low-level, pre-linguistic auditory transformation of the acoustic signal, (2) learning of changes in the linguistic representations, and (3) post-linguistic changes in decision-making biases (see, e.g., Johnson, 2006; Pardo and Remez, 2006; Xie et al., 2023). The present study focuses on the first type of account, that the acoustic signal is transformed and normalized early on during auditory processing (for recent reviews, Stilp, 2020; Johnson and Sjerps, 2021).

Accounts of pre-linguistic normalization are motivated by *a priori* considerations about both the physics of sounds (cf. the discussion of uniform scaling in Barreda, 2020) and evolutionary arguments (e.g., even non-human animals exhibit similar abilities, Barreda, 2020). They are also supported by brain imaging evidence: talker-normalized information about the speech signal can be decoded from areas as early as the brain stem (e.g., Skoe et al., 2021), and thus prior to even the earliest cortical areas typically associated with linguistic category representations or decision-making. While it is rather uncontroversial that normalization is part of adaptive speech perception, questions remain about the specific nature of the operations involved in normalization. We contribute to this line of research by comparing different types of normalization accounts against vowel production data from a new phonetically annotated database of Central Swedish vowels (the SwehVd database).

**Figure 1:**
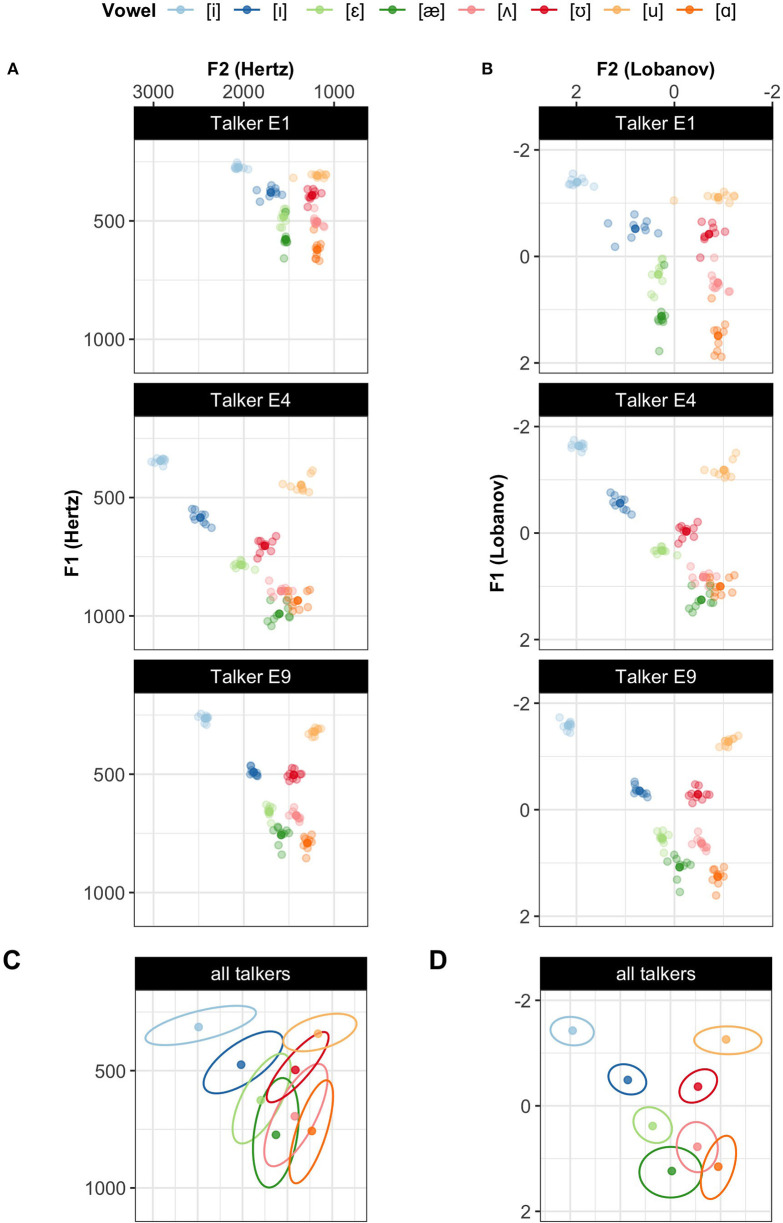
Illustrating how normalization reduces category overlap for the 8 monophthongs of L1 US English. Three talkers from the Xie and Jaeger (2020) database are shown before **(A)** and after Lobanov normalization (Lobanov, 1971)—one of the most commonly applied accounts **(B)**. Lobanov normalization reduces inter-talker variability in the category means and, to some extent, in the category variances. The bottom two panels aggregate the data from all 17 talkers in the database (5 female, 12 male), showing the means and 95% probability mass bivariate Gaussian densities for each vowel before **(C)** and after Lobanov normalization **(D)**.

Normalization accounts were originally proposed as a theory of how the brain removes *physiologically*-caused variation from the speech signal (e.g., Peterson, 1961; Gerstman, 1968; Lobanov, 1971; Nordström and Lindblom, 1975; Nearey, 1978; Bladon et al., 1984; Sussman, 1986; Syrdal and Gopal, 1986; Miller, 1989). Much of this early work focused specifically on differences in formants, the primary cues to the perception of vowel quality. These formants—peaks in the energy distribution over frequencies—are affected by talkers' vocal tract size (e.g., Peterson and Barney, 1952; Verbrugge and Shankweiler, 1977; Fox et al., 1995; Yang and Fox, 2014). Successful normalization was meant to account for these physiological differences, thereby reducing inter-talker variability in the phonetic realization of vowels (compare Figure 1B and Figure 1A), which can result in reduced category overlap (compare Figure 1D and Figure 1C).^1^

Over the decades, dozens of competing accounts of vowel normalization have been proposed (e.g., Joos, 1948; Gerstman, 1968; Lobanov, 1971; Fant, 1975; Nordström and Lindblom, 1975; Nearey, 1978; Traunmüller, 1981; Bladon et al., 1984; Syrdal and Gopal, 1986; Miller, 1989; Zahorian and Jagharghi, 1991; Watt and Fabricius, 2002; for reviews, see Weatherholtz and Jaeger, 2016; Barreda, 2020). Carpenter and Govindarajan (1993) summarize over 100 different vowel-specific accounts, though—as we discuss later in more detail—many of them share the same basic operations. More recently, additional *general* normalization accounts have emerged that can be applied to *any* type of cue and phonological contrast, rather than just vowel formants (e.g., Cole et al., 2010; McMurray and Jongman, 2011). The most widely used of these proposals, C-CuRE, has since been successfully applied to the categorization of US English fricatives (McMurray and Jongman, 2011; Apfelbaum et al., 2014; Crinnion et al., 2020), stop voicing (Toscano and McMurray, 2015; Kulikov, 2022; Xie et al., 2023), sentence-final rising question vs. statement intonation (Xie et al., 2021), as well as vowels (Kleinschmidt, 2019). In each of these studies, C-CuRE reduced inter-talker variability and improved categorization. C-CuRE, which stands for **c**omputing **cu**es **r**elative to **e**xpectations, captures the motivation behind earlier normalization accounts that the acoustic-phonetic properties of the current speech input should be interpreted relative to their expected distribution in the present context. Unlike many of these earlier accounts, however, C-CuRE is not just meant to account for expectations based on talkers' *physiology* but applies equally to expectations based on, for example, talkers' social identity or language background. This makes C-CuRE a potential candidate mechanism for adaptive speech perception beyond physiological effects on vowel formants, and is the reason we include it in our comparison of normalization accounts.

### 1.1. The present study

Table 1 lists the normalization accounts investigated in the present study. This includes both the most influential vowel-specific normalization accounts that have been found to perform well in previous works (e.g., Lobanov and Nearey2 normalization) and several variants of the general purpose normalization C-CuRE. As indicated through shading in the table, the accounts can be grouped into four types based on the computational assumptions they make. *Transformations* are meant to transform the formant data from acoustic (Hz) into a perceptual space that approximates the perceptual organization of auditory information in the human brain. All other accounts instead or additionally adjust each formant value based on either the values of other formants on the same segment (*vowel-intrinsic* approaches) or summary statistics of the formant across segments (*vowel-extrinsic* approaches).^2^ We further distinguish two types of vowel-extrinsic approaches that differ in their computational complexity and tractability: approaches that *center* each cue relative to its mean across all vowel segments, and approaches that instead/additionally *standardize* cues relative to the overall variability or range of the cue across all vowel segments (for reviews, see also e.g., Johnson, 2005; Kohn and Farrington, 2012; Weatherholtz and Jaeger, 2016).^3^ The former type includes C-CuRE, and we consider different variants of this approach, one for each transformation approach in Table 1.

**Table 1:** Normalization accounts considered in the present study.

		**Normalization procedure**	**Perceptual scale**	**Source**	**Formula**
		**None**	**Hz**	**n/a**	**n/a**
transformation		none	Bark	Traunmüller, 1990	$F_{n}^{Bark}=\frac{26.81\times F_{n}}{1960+F_{n}}-0.53$
		—	ERB	Glasberg and Moore, 1990	$F_{n}^{ERB}=21.4\times \log_{10}\left(1+F_{n}\times 0.00437\right)$
		—	Mel	Stevens and Volkmann, 1940	$F_{n}^{Mel}=2595\times \log_{10}\left(1+\frac{F_{n}}{700}\right)$
		—	Semitones conversion	Fant et al., 2002	$F_{n}^{ST}=12\times \frac{ln\left(\frac{F_{n}}{100}\right)}{ln}$
intrinsic		Syrdal and Gopal's	Bark	Syrdal and Gopal, 1986	*F*1^*SyrdalGopal*^ = *F*1^*Bark*^−*F*0^*Bark*^
		Bark-distance model^a^			*F*2^*SyrdalGopal*^ = *F*2^*Bark*^−*F*1^*Bark*^
		Miller	log	Miller, 1989	$SR=k{\left(\frac{GMf0}{k}\right)}^{1/3}$
		(formant-ratio)			$F1^{Miller}=log\left(\frac{F1}{SR}\right)$
					$F2^{Miller}=log\left(\frac{F2}{F1}\right)$
					$F3^{Miller}=log\left(\frac{F3}{F2}\right)$
extrinsic	centering	C-CuRE	Hz	McMurray and Jongman, 2011	$F_{n}^{C-CuRE}=F_{n}-mean\left(F_{n}\right)$
		—	Bark		
		—	ERB		
		—	Mel		
		Nearey1	log	Nearey, 1978	$F_{n}^{Nearey1}=\ln\left(F_{n}\right)-mean\left(ln\left(F_{n}\right)\right)$
		(log-mean)			
		Nearey2	log	Nearey, 1978	$F_{n}^{Nearey2}=\ln\left(F_{n}\right)-mean\left(ln\left(F\right)\right)$
		(single log-mean)			
	standardizing	Gerstman	Hz	Gerstman, 1968	$F_{n}^{Gerstman}=999\times \frac{F_{n}-F_{n}^{min}}{F_{n}^{max}-F_{n}^{min}}$
		(range normalization)			
			
		Lobanov	Hz	Lobanov, 1971	$F_{n}^{Lobanov}=\frac{F_{n}-mean\left(F_{n}\right)}{sd\left(F_{n}\right)}$
		(z-score)			

Unless otherwise marked, formant variables (*F*s) in the right-handside of normalization formulas are in Hz. F_*n*_ refers to the *n*th formant. F refers to the vector of all formants.

^a^Previous work has considered two different implementations of Syrdal & Gopal's Bark-distance model for F2, depending on the language (Fant, 1983; Syrdal and Gopal, 1986; Adank, 2003). In the SI (Section 3), we compare these two implementations, and find that the F2-F1 implementation performs better for the present data. We thus present that version of Syrdal & Gopal's model in the main text.

The selection of accounts we consider in the present study is primarily based on their influence and performance in previous evaluations against other data sets. Additionally, we only consider accounts that are sufficiently general in nature to be applied across languages. This decision stems from our goal to understand the mechanisms underlying *human* speech perception. This means that we for instance do not include Watt and Fabricius (Watt and Fabricius, 2002; Fabricius et al., 2009), as this account requires specific assumptions of vowel inventories of the language. Finally, we do not consider *combinations* of accounts. This follows the majority of previous work but is an important limitation that we return to in the general discussion.

Existing evaluations of normalization accounts can be broadly grouped into two types: studies that compare accounts in terms of their effectiveness in reducing inter-talker variability in the phonetic realization of categories (Table 2), and studies that compare accounts in terms of their expected consequences for perception (Table 3). While the two approaches have often yielded similar results, they measure different aspects, and do not *have to* agree. As we show in Supplementary Section 7 and discuss after the presentation of our results, measures of between- vs. within-category separability/variability have downsides that can lead to misleading results. Simply put, reduction of variance is not the ultimate goal of speech perception, and reduced variance does not always result in improved perception. We thus focus on the second approach, as our ultimate interest is in evaluating normalization as a hypothesis about the mechanisms underlying adaptive speech perception. We note, however, that the present study is limited to evaluating the *predicted* consequences for perception, rather than the *fit* of different normalization accounts against perception data. This limitation is shared with the majority of previous work—very few studies to date have compared normalization against listeners' responses in perception experiments (Nearey, 1989; Richter et al., 2017; Barreda, 2021). We return to this important caveat in the discussion.

**Table 2:** Previous studies comparing the effectiveness of normalization accounts in reducing within-category cue variability.

**Language investigated**	**Article**	**Speech materials**	**Normalization accounts**	**Approach**	**Best two performing**
US English	Barreda and Nearey, 2018	120,000 simulated languages (of 5 or 9 vowels) modeled on Hillenbrand et al.'s (1995) data (98 female/male child/adult talkers * 12 vowels)	Nearey2, Lobanov, log-mean in linear regression framework	Distance between means (Eucledian distance)	Log-mean in linear regression framework (1), Nearey2 (2)
	Clopper, 2009	2 female/male talkers from Ohio (1 token * 10 vowels)	Bladon et al. (1984) scale factor of 1 Bark, Syrdal and Gopal, Nordström and Lindblom, Nearey1, Nearey2, Watt and Fabricius, Gerstman, Lobanov, Miller	Variance reduction (visual inspection)	Nearey, Watt and Fabricius, Gerstman, Lobanov (no order)
	Hindle, 1978	Peterson and Barney's (1952) database; 19 female/male talkers from Philadelphia + 60 telephone informants (minimum 3 tokens per category; analysis focus on /ay/)	Nearey2, Nordström-Lindblom, Sankoff-Shorrock-McKay	Distance between means, variance reduction (regression)	Sankoff (1)
	Kohn and Farrington, 2012	Longitudinal data from 10 female/male African American talkers from North Carolina (approx. 10 tokens * 10 vowels * 5 ages)	Lobanov, Gerstman, Nearey1, Nordström and Lindblom, Syrdal and Gopal/Thomas, Watt and Fabricius	Variance reduction (regression)	Lobanov (1), Gerstman, Watt and Fabricius (2)
	Labov, 2010	Peterson and Barney's (1952) database; Philadelphia/Linguistic Change and Variation project (120 female/male talkers, stratified for age, sociolinguistic factors)	Nearey2, Nordström-Lindblom, Sankoff-Shorrock-McKay	Distance between means (F-statistics)	Sankoff (1), Nearey2 (2)
US English, Norwegian, Swedish, German, Danish, Dutch	Disner, 1980	Differing number of tokens, vowels, and phonetic contexts across the six languages	Gerstman, Lobanov, Nearey2, Harshman's PARAFAC model	Variance reduction (visual inspection)	Nearey2 (1), Lobanov (2)
UK English	Fabricius et al., 2009	20 old/young female/male talkers of Received pronunciation (11 vowels); 6 old/young female/male talkers of Aberdeen English (8 vowels in different phonetic contexts)	Watt and Fabricius, Lobanov, Nearey1	Variance reduction (SCV in talker-means)	Lobanov (1), Watt and Fabricius (2)
	Flynn and Foulkes, 2011	20 old/young female/male Nottingham talkers (mean 180 recordings per talker; categories not reported)	log-transformation (base 10), log-transformation (natural), Mel, ERB, Bark (*2 gender-specific versions), Syrdal and Gopal, Nordström (*2 gender-specific versions), LCE, Gerstman, Lobanov, Watt and Fabricius (* 4 versions), lettER, Nearey (*4 versions)		Gerstman (1), LCE (2)
Russian	Lobanov, 1971	5 female/male talkers (9 vowels in different phonetic contexts)	linear compression or expansion (Fant, 1960), Gerstman, Lobanov	Distance between means	Lobanov (1), Gerstman (2)

**Table 3:** Previous studies comparing normalization accounts in terms of their predicted consequences for perception.

**Language(s) investigated**	**Article**	**Speech materials**	**Normalization accounts**	**Approach**	**Accuracy assessed**	**Best two performing**
US English	Barreda, 2021	Synthesized stimuli representing 6 talker types (based on data from 30 female/male talkers of California English (15 tokens * 11 vowels))	Nearey2, Watt and Fabricius, Lobanov	Regression	Against perceived category	Nearey2 (1), Watt and Fabricius (2)
	Carpenter and Govindarajan, 1993	Peterson and Barney's (1952) database, 75 female/male child/adult talkers (2 tokens * 10 vowels)	Bark, Mel, ERB, 2 log-transformations, Syrdal and Gopal, Miller, Nearey1, Nearey2, Gerstman, linear transformation (Watrous, 1993)	Fuzzy ARTMAP, K-nearest neighbor	Against intended category	Linear transformation (1), Nearey1 (2)
	Cole et al., 2010	10 female/male talkers (3 tokens * 2 target vowels * 4 context vowels * 6 consonants)	C-CuRE	Regression		C-CuRE (1)
	Johnson and Sjerps, 2021	Peterson and Barney's (1952) database, 75 female/male child/adult talkers (2 tokens * 10 vowels); Hillenbrand et al.'s (1995) database, 138 female/male child/adult talkers (1–3 tokens * 12 vowels)	Mean λ, F3 anchor, F1 anchor, Mean F* anchor (Sussman, 1986), Nordström, VTLN (Lammert and Narayanan, 2015), Nearey2, Gerstman, VTLN (ΔF), Nearey1, Watt and Fabricius, Lobanov, Miller, Syrdal and Gopal	Support vector machine classification models		Lobanov (1), Watt and Fabricius (2)
	McMurray et al., 2011	Cole et al. (2010) database, 10 female/male talkers (1 token * 2 target vowels * 4 context vowels * 6 consonants)	C-CuRE	Regression		C-CuRE (1)
	Nearey, 1989	Synthesized stimuli of male child/adult talker (based on male talker data from Fant, 1973, and Peterson and Barney, 1952)	Intrinsic normalization, extrinsic normalization	Response patterns (F-ratio)	Against perceived category	Extrinsic effects (1), intrinsic effects (2)
	Richter et al., 2017	Models based on Clopper and Pisoni's (2006) NSP vowel corpus, 60 female/male talkers, 6 varieties (5 tokens * 10 vowels); perceptual data from Feldman et al., 2009 (synthesized stimuli of male talker)	Vocal Tract Length Normalization (VTLN), Lobanov	Discrimination model likelihoods		VTLN (1), Lobanov (2)
	Syrdal, 1985	Peterson and Barney's (1952) database, 75 female/male child/adult talkers (2 tokens * 10 vowels)	Log-transformation, Bark, Syrdal's bark-difference model, Miller (2 accounts), Nearey1, Nearey2, Gerstman	Linear discriminant analysis	Nearey1 (1), Nearey2 (2)	Against intended category
Brazilian Portuguese and US English	Escudero and Bion, 2007	Models trained on 400,000 F1-F2 combinations generated on recordings of 8 female/male talkers (20 tokens * 7 vowels and 15 tokens * 11 vowels)	Nearey1, Lobanov, Gerstman	Constraint rankings	Lobanov (1), Nearey1 (2)	
Dutch	Adank et al., 2004	160 female/male talkers, 8 varieties (2 tokens * 9 vowels)	Log-transformation, Bark, Mel, ERB, Syrdal, and Gopal, Lobanov, Nearey1, Nearey2^a^, Gerstman, Nordström, Miller	Linear discriminant analysis		

^a^Barreda and Nearey (2018) identify a mistake in the implementation of the Nearey2 account in Adank et al. (2004), so that the relative performance of Nearey2 reported by Adank and colleagues should be interpreted with caution.

Several generalizations emerge from Tables 2, 3. First, transformations of the acoustic input to a perceptual scale alone are not particularly effective at reducing variability or improving recognition (see also Carpenter and Govindarajan, 1993; Adank et al., 2004; Escudero and Bion, 2007; Clopper, 2009; Flynn and Foulkes, 2011; Kohn and Farrington, 2012). Accounts that additionally apply intrinsic or extrinsic normalization perform significantly better. In particular, extrinsic normalization accounts that center and/or standardize formants seem to perform best both in reducing inter-talker variability (see, e.g., Lobanov, 1971; Disner, 1980; Fabricius et al., 2009; Labov, 2010; Kohn and Farrington, 2012; Barreda and Nearey, 2018) and in improving recognition (e.g., Syrdal, 1985; Adank et al., 2004; Escudero and Bion, 2007; Johnson and Sjerps, 2021). When Lobanov and Gerstman normalization—both involving standardizing—were included in a study, they often rank among the top two performing accounts. Of note, Nearey normalization (Nearey, 1978) often performs well even though it does not involve the computationally more complex operation of standardizing. This suggests that simple centering of formants relative to the talker's mean *might* be sufficient to achieve significant variance reduction (but see Disner, 1980 for Swedish, which is revisited in this study).

In the present study, we go beyond previous work by modeling the effects of normalization on the predicted perception of both vowel quality and vowel quantity over a particularly dense vowel space. Previous comparisons of normalization accounts have primarily focused on English (e.g., Hindle, 1978; Disner, 1980; Syrdal, 1985; Carpenter and Govindarajan, 1993; Adank et al., 2004; Escudero and Bion, 2007; Clopper, 2009; Fabricius et al., 2009; Labov, 2010; Flynn and Foulkes, 2011; Kohn and Farrington, 2012; Richter et al., 2017; Barreda and Nearey, 2018). Additional studies have investigated, for example, Dutch (Disner, 1980; Adank et al., 2004), Russian (Lobanov, 1971), and Brazilian Portuguese (Escudero and Bion, 2007). The complexity of the vowel inventories (7–11 monophthongs) and the number of these vowels included in the comparison (2–11) varied across these studies. We add to this literature by comparing normalization accounts against a new phonetically annotated database of Central Swedish (SwehVd, introduced below). With a total of 21 monophthong allophones that vary in quantity (long vs. short vowels) and quality, the vowel inventory of Swedish is crowded compared to most languages previously studied in the normalization literature. This allows us to test whether the same normalization accounts that work well for simpler vowel inventories generalize well to more crowded vowel spaces.

To the best of our knowledge, only one previous study has compared normalization accounts against Swedish, as part of a cross-linguistic comparison across six Germanic languages (Disner, 1980). Disner (1980) compared 4 normalization accounts, using F1 and F2 means of the nine long Swedish vowels spoken by 24 male Swedish talkers (from a database presented in Fant et al., 1969). Of interest to the present study, the results for Swedish differed from the other Germanic languages in two unexpected ways. Whereas Lobanov normalization—which involves centering and standardizing—performed best for Swedish, Nearey2 normalization—which involves only centering—performed best for the other four languages. And, while normalization effectively reduced inter-talker variability in category variances for the other four languages by 61–71%, it was substantially less effective for Swedish (41%). As discussed by Disner (1980), this raises the question as to whether these findings reflect an inherent property of Swedish or merely differences in the phonetically annotated databases available for each language. In particular, the Swedish data consisted of *vowels* produced in isolation without any lexical or phonetic context, whereas the data for the five other languages consisted of isolated *word* productions (paralleling the majority of research on normalization). The present study addresses this difference: the new database we introduce consists of *h*-VOWEL-*d* word recordings, which makes our stimuli directly comparable to those used in previous work on normalization, and lets us revisit whether simple *centering* accounts perform best for Swedish—like for the other languages in Disner (1980). Additionally, we complement Disner's study by focusing on female, rather than male talkers, and by considering both long and short vowels (separately and together). The presence of quantity contrasts between long and short allophones makes Swedish a suitable case study to bridge the literature between vowel-specific normalization accounts (which focus on formants, and thus only quality contrasts) and general normalization accounts that can be applied to any type of cue (and thus also vowel duration, which is expected to be the primary cue to vowel quantity). While both F3 and vowel duration are known to be important cues to vowel categorization in Swedish (e.g., Hadding-Koch and Abramson, 1964; Fujimura, 1967; Behne et al., 1997), the two cues have never (duration) or rarely (F3, but see, e.g., Syrdal, 1985; Nearey, 1989; Carpenter and Govindarajan, 1993; Adank et al., 2004; Barreda and Nearey, 2018) been included in comparisons of normalization accounts.

We compare the normalization accounts in Table 1 in terms of the predicted consequences for perception. The study compares accounts applied to (1) only F1 and F2, as in the majority of previous studies, (2) F1-F3, as in, e.g., Adank et al. (2004), and (3) F0-F3 as well as vowel duration. This allows us to assess whether differences in the effectiveness of normalization accounts depend on the number and types of cues that are considered. Since listeners integrate cues beyond F1 and F2 (e.g., Assmann et al., 1982; Nearey and Assmann, 1986; Hillenbrand and Nearey, 1999), this is an important gap in evaluating the plausibility of different normalization accounts as models of adaptive speech perception. All three comparisons are evaluated both separately for short and long vowels, and for the entire space of the 21 vowels. This allows us to assess whether the same types of normalization perform well across the entire vowel inventory.

As shown in Table 3, previous work has employed a number of model types to compare the expected effects of normalization on perception, ranging from models based on phonological theory (e.g., optimality theory, Escudero and Bion, 2007), to more general models of categorization (e.g., linear discriminant analysis, Syrdal, 1985; Adank et al., 2004; k-nearest neighbors as in exemplar theory or ARTMAP, Carpenter and Govindarajan, 1993; Bayesian inference, Richter et al., 2017; Kleinschmidt et al., 2018; support vector machine classification models, Johnson and Sjerps, 2021), to general frameworks for data analysis (e.g., regression, Cole et al., 2010). In this study, we use a general model of speech perception, Bayesian ideal observers (e.g., Nearey and Hogan, 1986; Clayards et al., 2008; Norris and McQueen, 2008), to predict the vowel identities in the SwehVd database under different normalization accounts. We then compare normalization accounts based on the recognition accuracy that they achieve when the (un)normalized cues are fed into the otherwise identical categorization model. We repeat this comparisons for different combinations of cues, and while categorizing different subsets of the vowel space. We use ideal observers, rather than other approaches, because *all* of their degrees of freedom can be estimated from the phonetic database we use (see also Tan et al., 2021; Xie et al., 2023). In contrast, k-nearest neighbor categorization introduces the choice of a similarity metric, which can introduce one or more degrees of freedom into the modeling, and requires a choice for *k*. Similarly, linear discriminant analysis, support vector machines, or regression introduce *at least* one degree of freedom for each cue considered. This means that any comparison of normalization accounts needs to be conducted over the entire range of possible values for these degrees of freedom, making comparisons computationally more demanding and interpretation of the results more difficult. Bayesian ideal observers avoid this issue because of their assumption that listeners use and integrate cues *optimally*. As a consequence, the predicted posterior probabilities of all categories are fully determined by the combination of (1) the category-specific distribution of cues in the previous input and (2) the cue values of the input. The ideal observer approach employed here thus minimizes the degrees of freedom in the model that are not fully determined by the cue statistics in the input.

All data and code for this article can be downloaded from OSF at https://osf.io/zb8gx/. This article is written in R markdown, allowing readers to replicate our analyses using freely available software (RStudio Team, 2020; R Core Team, 2021), while changing any of the parameters of our models. Readers can revisit and alter the assumptions we make—for example, categorization method, models of linguistic representations, the normalization accounts selected. The Supplementary material lists the software/libraries required to compile this document.

## 2. Methods

We begin by introducing the new phonetically annotated corpus of Central Swedish vowel productions used in the present study. We then present the perceptual model that we use for assessing the predicted effects of different normalization accounts—a Bayesian ideal observer.

### 2.1. Materials: the SwehVd database

The SwehVd database is a new phonetically annotated corpus of Swedish *h*-VOWEL-*d* (short: hVd) word recordings. All recordings, annotations, and acoustic measurements are available on an OSF separate from the paper, at https://osf.io/ruxnb/. SwehVd was collected with the goal to characterize the Central Swedish vowel space within and across talkers—specifically, the regional standard variety of Swedish spoken in an area around and beyond Stockholm (eastern Svealand), including Mälardalssvenska, Sveamål, Uppsvenska, Mellansvenska (see, e.g., Elert, 1994; Bruce, 2009; Riad, 2014).

SwehVd covers the entire monophthong inventory of Central Swedish, including all nine long vowels (*hid, hyd, hud, hed, häd, höd, had, håd, hod*), eight short vowels (*hidd, hydd, hudd, hedd, hädd, hödd, hadd, hådd, hodd*), and four allophones (*härd, härr, hörd, hörr*). To our knowledge, there are few publicly available databases of Swedish vowel productions that are phonetically annotated (e.g., Fant et al., 1969; Eklund and Traunmüller, 1997; Bruce et al., 1999; Kuronen, 2000). The largest and perhaps best-known is SweDia 2000 (Bruce et al., 1999). SweDia 2000 was developed to characterize differences in vowel pronunciations *across* regional varieties of Swedish. It consists of recordings of spontaneous speech, isolated words in varying phonological contexts, and phrases in isolation from approximately 1300 talkers of 107 regional backgrounds, with 10-12 recorded talkers per region and 5-15 recordings per vowel for each talker.

Unlike most existing databases, SwehVd focuses on a single regional variety, providing high resolution within and across talkers for this variety: SwehVd consists of *N* = 10 recordings of each hVd word (for a total of 220 recordings for the 22 different hVd words) per talker. Specifically, we target *N* = 24 male and female talkers each (current *N* = 24, all female) for a total targeted *N* of tokens = 10,560 (current *N* = 4,731 tokens). The database contains first to third formant (F1-F3) measurements for each talker at five time points across each vowel, together with vowel duration and mean F0 over the entire vowel.

SwehVd follows the gross of research on normalization and uses hVd words for recording in order to minimize coarticulatory effects from the surrounding phonetic context. The hVd context was originally chosen for studies on English because the glottal /h/ in onset position minimizes supraglottal articulations (confirmed in, e.g., Chesworth et al., 2003; Robb and Chen, 2009). Since then hVd words have played a central role in research on vowel production (e.g., Peterson and Barney, 1952; Hillenbrand et al., 1995) and perception (e.g., Peterson and Barney, 1952; Malinasky et al., 2020). Since Swedish onset /h/ is a glottal approximant (Riad, 2014) similar to English, the use of this context in SwehVd facilitates comparison to similar databases from other languages. It deviates, however, from the majority of previous studies on Swedish vowels, which have either not held phonetic context constant across vowels (e.g., Bruce et al., 1999), or have investigated vowel production out of context (Fant et al., 1969; Disner, 1980; Eklund and Traunmüller, 1997) or in different CVC contexts (e.g., *k*V*p* and *p*V*k* in Nordstrand et al., 2004; *v*V*t, v*V*tt, f* V*t, f* V*tt*, in Behne et al., 1997).

#### 2.1.1. The Swedish vowel inventory

The Central Swedish vowel inventory contains 21 monophthong vowels. Seventeen of these vowels form nine pairs distinguished by quantity (long and short): in Central Swedish, the two long vowels [εː] and [eː] both neutralize to the same short vowel [ε] (resulting in a total of 17, rather than 18, distinct vowels). The two variants of a pair are considered allophones, the selection of which is determined primarily by stress and syllable complexity. Quantity is neutralized in unstressed positions (Riad, 2014).^4^ Vowels lengthen in open word-final syllables, before morpheme-final single consonants, and in non-final syllables.

Additionally, there are four contextually conditioned allophones to [ε] and [ø]. Before /r/ (or any retroflex segment), both the long and short versions of these vowels lower to long and short [æ] and [œ], respectively. As shown in Table 4 (adapted from Riad, 2014), some long-short vowel pairs are described to differ not only in quantity but also in quality: generally, short vowels are described as more open and also more centralized, forming a more condensed vowel space. In ongoing work (Persson, 2023), we found this to be confirmed for SwehVd.

**Table 4:** The phonetic characterization of long (left) and short (right) Central Swedish vowels (based on Riad, 2014).

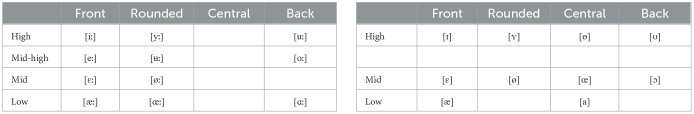

Several of the long vowels have been claimed to be diphthongized in Central Swedish (e.g., Fant et al., 1969; Fant, 1971; Elert, 1981; Kuronen, 2000) and/or with consonantal elements (McAllister et al., 1974), though empirical evaluations of this claim have returned mixed results (Fant et al., 1969; Eklund and Traunmüller, 1997; Leinonen, 2010). Here we do not discuss this issue further (but see Persson, 2023) since it is unclear how the presence of diphthongization would *bias* our results (rather than to lead to worse performance across all accounts).

#### 2.1.2. Participants

L1 talkers of Stockholm Swedish were recruited through word-of-mouth, flyers at Stockholm University Campus (see example flyer in Supplementary Section 2.1), and online channels (accindi.se). Participants were selected based on the following criteria: L1 talkers of Swedish, born and raised in the greater Stockholm area or its surroundings, 20–40 years old (mean age = 28; SD = 5.45). All participants were reimbursed with a voucher to the value of SEK 100 after completing the recordings.

#### 2.1.3. Recording procedure

Recording for the SwehVd database began in 2020 and is ongoing. The data were collected by the first author and Maryann Tan (Stockholm University). The hVd words were recorded together with another set of recordings targeting the production of Swedish word-initial stop voicing. Recording took place in a sound-attenuated room at the Multilingualism Laboratory, Department of Swedish Language and Multilingualism, Stockholm University.

Prior to recording, participants were informed about the study and given the possibility to ask questions before signing a consent form. They were then given instructions and seated at approximately 10 cm distance from an Audio Technica AT3035 microphone facing a computer screen. Words were presented one at a time, centered on screen, using PsychoPy software (Peirce et al., 2019). Participants were instructed to read the words with their natural voice as they appeared on screen. Each talker read the same 22 target words, with 48 mono- and bi-syllabic filler words interspersed. Each target word was repeated 10 times and each filler word was repeated five times, generating a total of 460 productions per talker, 220 target productions and 240 filler productions. We generated two pseudo-randomized lists of the words, each list divided into four different blocks. Words were blocked across block lists and randomized within block lists, with the constraint that the same word would not appear more than twice in succession. Each participant was randomly assigned to one of the two lists. The pace of the presentation of the words was controlled by the experimenter, who was listening over Sennheiser HD215 headphones in the next room. A Yamaha MG102c mixing console with a built-in preamplifier was used together with a high-end ground isolator for preventing signal interference (Monacor FGA-40HQ). The speech was recorded at 44.1 kHz in Audacity (Audacity, 2021). Each long sound file was split into individual short sound files of one word each. The boundaries of each file were slightly trimmed and the files were labeled with the target word. All sound files from the same talker were concatenated into one long file before further processing.

The complete list of target hVd words is provided in Supplementary Table 1. It consists of four real Swedish words, *hed, härd, hörd, hud* (English translations: *heath, hearth, heard*, and *skin*, respectively) and 18 phonotactically legal pseudowords. Following Swedish orthographical conventions for quantity, we used orthographic *hVdd* to elicit the short vowel allophone (e.g., *hudd* for [ɵ]) and orthographic *hVd* to elicit the long vowel allophone (e.g., *hud* for [ʉː]). This orthography reflects systematic phonological process of complementary quantity in Swedish (Riad, 2014). In order to elicit the contextual allophones to [ε] and [ø], we added the supradental [ɖ] to elicit the long allophones (*härd, hörd*), and [r] to elicit the short allophones (*härr, hörr*). Challenges that came up during recording that were addressed are reported in Supplementary Section 2.3.

The recordings were divided into five blocks: one practice block and four recording blocks, with breaks in between. The purpose of the practice block was three-fold: to familiarize the participants with the recording procedure, to adjust the recording level, and if necessary, to further instruct the participant (e.g., if the participant used inappropriate or inconsistent intonation or stress pattern). Each recording block consisted of either 110 (*N* = 2 blocks) or 120 (*N* = 2 blocks) trials. The length of each block was approximately 8 min, for a total of roughly 30 min recording time per talker. After the recording, participants filled out a language background questionnaire and received their reimbursement.

#### 2.1.4. Word and vowel segmentation

SweFA, a Swedish version of the Montreal Forced Aligner developed by Young and McGarrah (2021), was used to obtain estimates for word and segment boundaries. The boundaries were manually corrected by the first author (an L1 talker of Central Swedish). Following standard segmentation protocol and guidelines in Engstrand et al. (2001), segment boundaries were adjusted using spectrogram, waveforms and pitch and intensity tracks. The boundaries between /h/ and the vowel were adjusted to align with clear appearance of an F1, and the boundaries between the vowel and the coda consonant were aligned to a simultaneous rapid cessation of most or all formants.

#### 2.1.5. Extraction of phonetic cues

**Figure 2:**
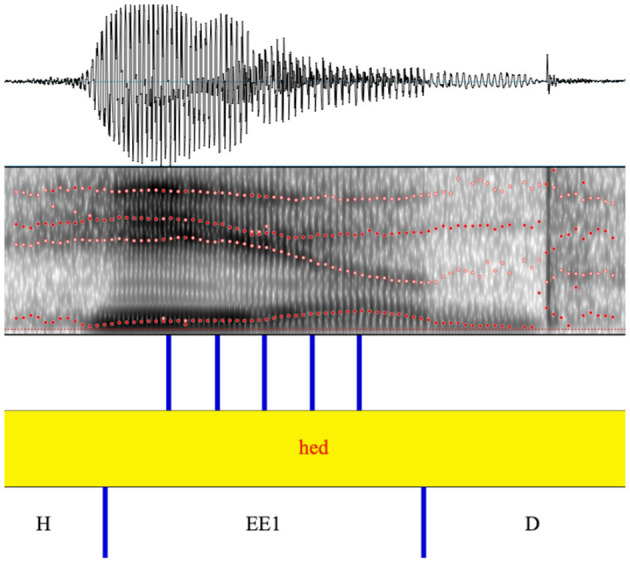
Example of Praat textgrid with annotated segment boundaries and measurement points for the automatic extraction of F1-F3 formant frequencies.

We used the Burg algorithm in Praat (Boersma and Weenink, 2022) to extract estimates of the first three formants (F1-F3) at five points of the vowel (20, 35, 50, 65, and 80 percent into the vowel; see Figure 2). The following parameterization of the Burg algorithm was used:

- Time step (s): 0.01
- Max. number of formants: 5
- Formant ceiling (Hz): 5,500
- Window length (s): 0.025
- Pre-emphasis from (Hz): 50.

In addition to F1-F3, we automatically extracted vowel duration and the fundamental frequency (F0) across the entire vowel. The Praat scripts that extract this information are shared as part of the SwehVd OSF repository, allowing researchers to choose additional or alternative time points at which to extract formants.

In order to correct for measurement errors in the automatic extraction of cues, we estimated the joint multivariate distribution along all five extracted cues (F0, F1, F2, F3, and vowel duration) for each unique combination of vowel and talker. This approach allowed us to detect outliers relative to the joint distribution of the five cues for that vowel and talker. Points outside of the 0.5th to 99.5th quantile of the multivariate Gaussian distribution of each vowel were identified, checked for measurements errors, and corrected. For measurements of the first three formants, we first checked the segmentation boundaries in the Praat textgrid and then manually measured new formant values using visual approximation of time points and Praat's function *Formant: Formant listing* or manually reading off the spectrogram. Segmentation boundaries were also checked for the identified vowel duration outliers. For measurements of F0, we extracted new estimated F0s across the vowel, after changing the pitch range settings. Given that there were still instances of pitch halving after measurement correction, in order to be conservative, we also checked all F0 values below the point of intersection between the two halves. This approach to F0 and formant correction strikes a middleground between the ideal (manual correction of all tokens) and feasibility. As SwehVd is open source, future work can contribute additional corrections to the database (e.g., via pull requests submitted to the repository linked on OSF). For the present purpose, additional undetected measurement errors are expected to bias *against* normalization, as outlier correction was conducted on the basis of raw F0 and formant values (Hz). If anything, the present study thus might under-estimate the effectiveness of normalization.

The procedure of adding written guides to *hod* and *hodd* to facilitate vowel identification was mostly successful, however not for all talkers. Some talkers corrected themselves after one trial, others failed to produce the intended vowel altogether. The SwehVd database contains columns for both the targeted vowel category, and the vowel category that the talker actually produced (as annotated by the first author).

#### 2.1.6. Characterizing vowel productions in SwehVd

**Figure 3:**
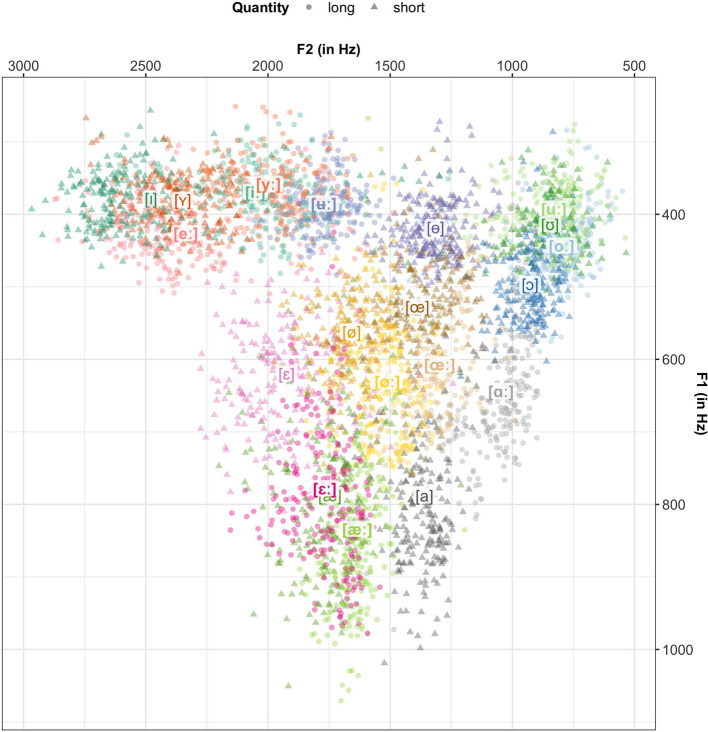
The SwehVd vowel data in unnormalized F1-F2 space. Points show recordings of each of the 21 Central Swedish vowels by the 24 female L1 talkers in the database, averaged across the five measurement points within each vowel segment. Vowel labels indicate category means across talkers. Long vowels are boldfaced. Vowels that mismatched intended label are excluded (1.18% of all recordings).

Figure 3 visualizes the vowel data from the SwehVd in F1-F2 space. The plot highlights the density of the Central Swedish vowel space, the categories are numerous and closely located. Category overlap is especially large among some of the high vowels (e.g., [iː] & [yː]; [uː], [oː] & [ʊ]). The contextually conditioned allophone [æ], almost completely overlaps with the long [εː], whereas the contextual allophones to [ø] are more separated. Not all contextual allophones are articulated lower (higher F1) in relation to their phonemes (compare, e.g., Riad, 2014). They are, however, all articulated further back (lower F2). In line with Riad (2014), the short vowels are overall more centralized and form a more condensed space, whereas the long vowels are more dispersed.

**Figure 4:**
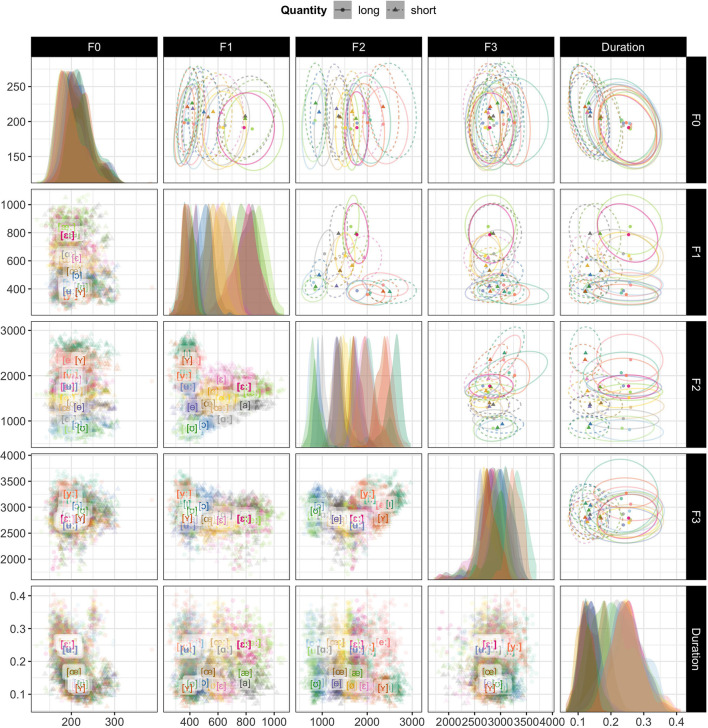
The same data as in Figure 3 but for all pairwise combinations of five cues: F0, F1, F2, F3, and vowel duration. The primary purpose of this figure is to provide an overview of the SwehVd data. Additionally, comparisons across the panels sheds light on which cues carry information about vowel quality and vowel quantity, respectively. Note that, unlike in Figure 3, axis directions are not reversed. **Panels on diagonal:** marginal cue densities of all five cues. **Lower off-diagonal panels:** each point corresponds to a recording, averaged across the five measurement points within each vowel segment. Vowel labels indicate category means across talkers. Long vowels are boldfaced. **Upper off-diagonal panels:** Same data as in the lower off-diagonal panels but showing bivariate Gaussian 95% probability mass ellipses around category means. This makes it more obvious, for example, that long and short vowels are primarily distinguished by vowel duration **(top right panel)**.

Figure 4 visualizes the vowel data from the SwehVd database for all pairwise combinations of five cues: F0, F1, F2, F3 and vowel duration. As is to be expected, vowels differing in quality are most separated in the F1-F2 plot, indicating the two cues most important for vowel category distinction. However, the F1-F3 and F3-F2 plots both display less overlap between the high vowels [iː], [yː], and [ʉː], comparing to when plotted along F1-F2. The increased separation of these categories along F3 in vowel production data could point to the importance of F3 for some category distinctions, as found in previous studies (see, e.g., Fujimura, 1967; Fant et al., 1969; Kuronen, 2000, for [iː] and [yː] categorization). Also as expected, duration is the primary cue that distinguishes vowel quantity: in the last column of Figure 4, the short vowels cluster on the left, and the long vowels on the right. They are separable, but overlapping. In addition to duration, F1-F3 can also carry information about vowels differing in quantity. This is evident, for example, for [iː] vs. [ɪ], [yː] vs. [ʏ], [ʉː] vs. [ɵ], [ɑː] vs. [a], [εː] vs. [ε] in F1-F2 space, and for [iː] vs. [ɪ], [yː] vs. [ʏ], [ʉː] vs. [ɵ] in F2-F3 space.

Finally, the densities along the diagonal of Figure 4 suggest that F0 carries the least information about vowel identity, exhibiting the least between-category separation, followed by F3. This, too, is not surprising: while some accounts use F0 to *normalize* F1 and F2 (e.g., Syrdal and Gopal, 1986; Miller, 1989), F0 is not considered an important cue to vowel identity by itself (for demonstrations that F0 can, however, have strong *in*direct effects on vowel categorization, see Barreda and Nearey, 2012; Barreda, 2020).

### 2.2. Exclusions

We use the SwehVd database with some exclusions. Since we are interested in assessing the effects of normalization, we excluded any productions on which the talker did not produce the targeted vowel. We then excluded all talkers (*N* = 7) with fewer than 5 remaining recordings for at least one of the vowels. This left data from 17 female L1 talkers, with on average 847 (se = 2.5) tokens per vowel (range = 815–865), for a total of 17,780 observations. We also exclude all *hädd* productions, as they elicited the same vowel as *hedd* (in line with Riad, 2014; see Supplementary Section 2.4). This way, we have about equally many tokens from all vowels, simplifying the cross-validation procedure presented below and facilitating visual comparisons across vowels in our figures.

Since our goal is to obtain a reliable estimate of the formant values during the steady state of the vowel, we use only the three formant measurements extracted from the middle of the vowel (at 35, 50, and 65% into the vowel).^5^

### 2.3. Modeling approach

#### 2.3.1. Cues included in the normalization

We compare the expected effects of different normalization accounts for the perception of Central Swedish vowels under three different assumptions about the relevant cues. The first comparison follows most previous research and focuses on the two primary cues to vowel perception, F1 and F2. The second comparison considers F3 in addition to F1 and F2, following Syrdal (1985), Nearey (1989), Adank et al. (2004), and Barreda and Nearey (2018).^6^ Finally, the third comparison includes F0 and duration in addition to F1-F3. Since Syrdal and Gopal (1986)'s bark-difference model only considers normalization along two dimensions—height, implemented as F1-F0, and backness, implemented as F2-F1—this account will only be included in the first comparison. Furthermore, given that C-CuRE is the only account that applies to any type of cue, we will consider duration as centered to each talker's mean (for the C-CuRE accounts), or as raw input (in ms; for all other accounts). We evaluate the predicted effects for perception both separately for long and short vowels, and on all 21 vowels together.

#### 2.3.2. Guarding against over-fitting: cross-validation

As shown in Table 1, many of the normalization accounts involve parameters that are set based on the data (e.g., Gerstman, 1968; Lobanov, 1971; Nearey, 1978; Miller, 1989; McMurray and Jongman, 2011). This raises the question of how much these parameters can be affected by outliers, or other issues such as over-fitting to the sample. Unlike previous work, we thus use five-fold cross-validation to obtain 5 separate estimates of model predictions for each combination of normalization procedure and cues. Specifically, we randomly split the data for each unique combination of talker and vowel into 5 even parts (folds). On each of the five-folds, we then fit the normalization parameters based on four of the folds (the training data) and evaluated the effects of the normalization on the fifth fold (the test data). This resulted in five model estimates for each combination of normalization procedure and cues. Our result graphs average over those folds.

#### 2.3.3. Ideal observers to predict the consequences of normalization for perception

Ideal observers provide an analytical framework for estimating how a rational listener would optimally behave in response to input (here: *n*-way alternative forced-choice categorization). Ideal observer models have been found to provide a good qualitative and quantitative fit against human speech perception (e.g., Clayards et al., 2008; Norris and McQueen, 2008; Feldman et al., 2009; Kleinschmidt and Jaeger, 2015; Kronrod et al., 2016; Xie et al., 2021). Unlike most other models of speech perception, ideal observers in their simplest form—as employed here—have zero degrees of freedom in the link from production to perception: once the ideal observer is trained on phonetic data from a database of *productions*, its predictions about *perception* are not mediated by additional parameters (unlike, e.g., exemplar models, connectionist accounts, or neural networks).

In line with influential theories of speech perception (e.g., exemplar theory, Johnson, 1997; Bayesian accounts, Nearey, 1990; Luce and Pisoni, 1998; Norris and McQueen, 2008; interactive-activation accounts and their offsprings, McClelland and Elman, 1986; Magnuson et al., 2020), ideal observers describe the posterior probability of a category as dependent both on the prior probability of the category in the current context, *p*(*category*), and the likelihood of the acoustic input under the hypothesis that it originates from the category, *p*(*cues*|*category*):


(1) $$\begin{array}{l} p(category|cues)=\frac{p(cues|category)\times p(category)}{\sum_{c}p(cues|category_{c})\times p(category_{c})} \end{array}$$

The category prior, *p*(*category*), describes how much the surrounding context favors each category. For the present study, the choice of category prior cannot affect the qualitative results since category priors are independent of the cues and held identical across all normalization accounts (category priors have a constant additive effect on the posterior log-odds of categories). We arbitrarily assume uniform category priors. Specifically, for ideal observers trained and tested on the long and short vowels separately, we model categorization as an 11- and 10-alternatives-forced-choice task, respectively, resulting in *p*(*category*) = 0.091 for the former and *p*(*category*) = 0.1 for the latter. For ideal observers trained and tested on the entire vowel space, we model categorization as a 21-alternatives-forced-choice task, resulting in *p*(*category*) = 0.048.

The likelihood, *p*(*cues*|*category*), describes the distribution of cues for each category. Here, we follow previous work and assume multivariate Gaussian distributions to describe the cue likelihood (e.g., Clayards et al., 2008; Kleinschmidt and Jaeger, 2015; Kronrod et al., 2016; Xie et al., 2021). That is, we use the model in Equation (2), where μ and Σ refer to the category mean and variance-covariance matrix of the category's multivariate normal distribution.^7^ In terms of representational complexity, the assumption of multivariate Gaussian categories strikes a compromise between exemplar storage (less representationally parsimonious, Johnson, 1997; Pierrehumbert, 2001) and cue integration over multiple separate univariate Gaussians (more parsimonious, Toscano and McMurray, 2010; see also Xie et al., 2023). Additionally, the multivariate approach entails optimal cue weighting, whereas optimal cue weights need to be determined separately for cue integration over independent univariate Gaussians.


(2) $$p(category|cues)=\frac{N(cues|\mu ,\Sigma )\times p(category)}{\sum_{c}N(cues|\mu_{c},\Sigma_{c})\times p(category_{c})}$$

Each ideal observer was trained on the training portion of the folded unnormalized and normalized data (using the R package MVBeliefUpdatr, Human Language Processing Lab, 2023), and subsequently evaluated on the held-out test fold. This means that the parameters of each normalization account (e.g., the cue means in C-CuRE) and the resulting category parameters (the μ_*c*_s and Σ_*c*_s for all categories) were set on the training data, and not changed for the test data. This reflects the realities of speech perception: although this is often ignored in evaluations of normalization accounts (e.g., McMurray and Jongman, 2011; Barreda, 2021), listeners do not *a priori* know the cue means, cue variance, etc. of an unfamiliar talker. Rather, listeners need to incrementally *infer* those statistical properties from the talker's speech input (for discussion and a model, see Xie et al., 2023). An additional advantage of cross-validation is that it gives us an estimate of the uncertainty about the model predictions. The performance of each ideal observer during test is assessed by calculating the ideal observer's predicted posterior probability of the *intended* category for each test token, under the accuracy-maximizing decision rule (criterion choice). Additional analyses not summarized here confirmed that all results replicate if Luce's choice rule is used instead.

## 3. Results

**Figure 5:**
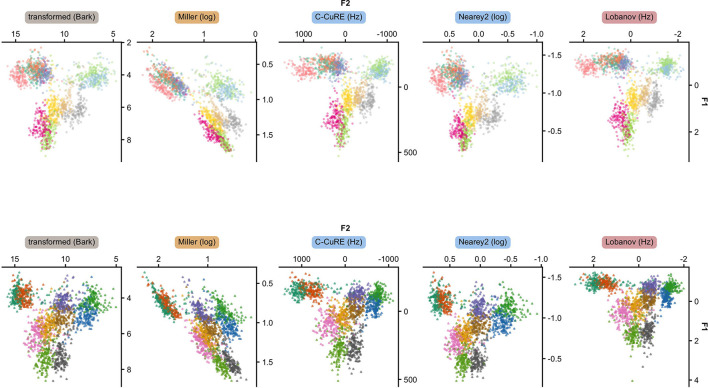
The 11 long vowels **(top)** and the 10 short vowels **(bottom)** of Central Swedish when F1 and F2 are transformed into a perceptual scale (gray), intrinsically normalized (yellow), or extrinsically normalized through centering (blue) or standardizing (purple). Each point corresponds to one recording, averaged across the five measurement points within each vowel segment. Each panel combines the data from all five test folds.

As an initial visualization of how normalization transforms the acoustic space, Figure 5 shows the transformed F1-F2 space for 5 of the accounts we evaluate. Supplementary Section 4 provides plots of all 15 accounts.

**Figure 6:**
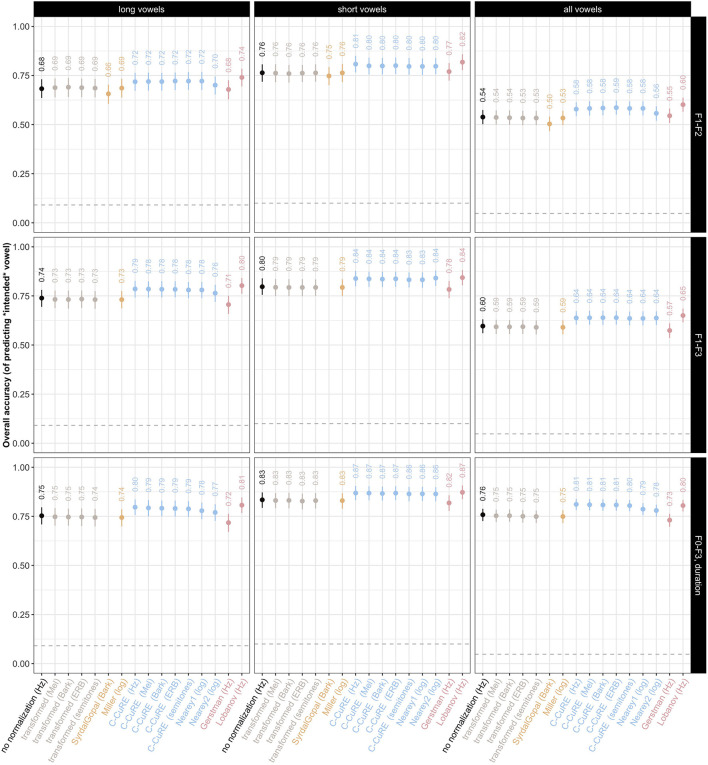
Predicted recognition accuracy of ideal observer under different normalization accounts for long vowels, short vowels, and all vowels together (columns), shown for three different combinations of cues (rows). Labels indicate mean across the five test folds. Intervals show average bootstrapped 95% confidence intervals across the test folds. The dashed horizontal line indicates chance (different across columns because of the different number of long and short vowels).

Figure 6 visualizes the unnormalized and normalized models' predictions for perception of Central Swedish vowels, under different assumptions about the relevant cues. This figure aggregates results across vowels of a given type (long, short, all). Additional studies in Supplementary Section 6 show results separately for each vowel, as well as visualizations summarizing how normalization affects the vowel-to-vowel confusion. These additional studies demonstrate, for example, that not all vowels benefit equally from normalization.

Averaging over all vowels, Figure 6 highlights that the relative performance of the different normalization accounts within each panel is remarkably constant across panels. Regardless of the combination of cues or the vowel types considered (long, short, all), transformation into a perceptual space does little to improve recognition accuracy, compared to unnormalized cues. Intrinsic normalization, too, does not improve recognition accuracy. This replicates previous work on Dutch (Adank et al., 2004) but conflicts with some evaluations of English (e.g., Syrdal, 1985). Adank et al. (2004) discussed whether the discrepancy in results might be attributed to implementations of the Bark-transformation, or to what Syrdal (1985) describes as language-specificity of the second dimension of Syrdal and Gopal (1986) normalization. The present results would seem to confirm this vulnerability of intrinsic normalizations. Extrinsic normalization, however, tends to substantially improve recognition accuracy (with the exception of Gerstman normalization). Depending on the specific combination of cues and the vowel qualities considered, the best-performing normalization model increases recognition accuracy by at least 60.2% (from 53.8% for unnormalized cues for all vowels when only F1-F2 are considered) to 87.2% (from 83.4% for short vowels when all cues are considered). The benefit of extrinsic normalization models, as well as the lower performance of perceptual transformations, replicates previous findings on other languages (e.g., Nearey, 1989 found effects of both intrinsic and extrinsic accounts, but larger effects for extrinsic; Adank et al., 2004; Escudero and Bion, 2007).

We also see that all models—even for unnormalized cues—perform substantially above chance. When long and short vowels are considered separately, the best ideal observers achieve recognition accuracies of 80.7% for long vowels and 87.2% for short vowels. For reference, in a recent perception experiment we conducted on the eight monophthongs of US English, L1-US English listeners achieved 71.1% accuracy in categorizing isolated hVd words (chance = 12.5%, Persson and Jaeger, 2023). A previous study on Swedish report an average recognition accuracy of 94.7% for the categorization of the long (isolated) vowels (Eklund and Traunmüller, 1997). The ideal observers for the Central Swedish vowel system thus achieve performance that is more or less comparable to that of human listeners, at least when cues are normalized.

Looking across columns of Figure 6, short vowels are always recognized with higher accuracy compared to long vowels. This increase in performance cannot be explained by the small increase in the chance baseline alone (10% for the 10 short vowels, compared to 9.1% for the 11 long vowels). This result might initially be puzzling, given that previous descriptions of Central Swedish vowel inventories characterize the inventory of short vowels as being more centralized and more densely clustered (e.g., Kuronen, 2000; Riad, 2014). Indeed, this claim seems to hold for SwehVd—compare Supplementary Figures 5, 6. However, they also exhibit less variability. Overall, this makes those vowels *easier* to recognize.

When long and short vowels are categorized together, performance of the ideal observers is comparatively poor unless vowel duration is included as a cue. This is expected given that vowel duration is the primary cue to vowel quantity. Of interest, however, is that even the inclusion of only F3 (second row) yields a substantial improvement in recognition accuracy, in line with Johnson and Sjerps (2021). Remarkably, once vowel duration is included, the best-performing ideal observer achieves 81.1% recognition accuracy across the 21 long and short vowels (compared to chance = 4.8%).

Finally, looking across rows, we note that Lobanov normalization performs best especially when only the first two formants are considered. However, this advantage of Lobanov normalization decreases when additional cues are considered.^8^

## 4. Discussion

We have compared low-level pre-linguistic normalization accounts against a new phonetically annotated database of Central Swedish vowels. We set out to evaluate how the different accounts differ in predicted consequences for perception. Previous work found that the types of normalization accounts that performed well on other languages did not seem to perform well on Swedish vowel data (Disner, 1980). However, as pointed out by Disner, the Swedish data differed from the data for other languages in that study, and the majority of studies on other languages. Here, we followed the majority of previous work on vowel productions and analyzed productions of hVd recordings. We find that the same accounts found in previous work to perform well on other languages also perform well for the dense vowel space of Swedish. Specifically, Lobanov and centering approaches—incl. Nearey normalization and C-CuRE normalization—were the top-performing accounts, replicating the pattern found in previous studies on other languages (e.g., Syrdal, 1985; Carpenter and Govindarajan, 1993; Adank et al., 2004; Escudero and Bion, 2007). This result suggests that the (somewhat) diverging results for Swedish in Disner (1980)'s study, were not caused by properties inherent to Swedish, but more likely were an artifact of the dataset employed by Disner. It also suggests that languages with dense vowel spaces do not necessarily require more complex normalization mechanisms.

Evaluating the predicted effects of normalization against SwehVd has allowed for a comparison of how normalization accounts perform on subsets of a large vowel space and on the entire vowel space, while also evaluating the combined effects of different cues. By comparing performance on long and short vowels separately and together, we found that category variability seems to have a larger impact on model performance than the dispersion of the categories in the space. The highest model performance was achieved when models were trained on the short vowels that are more densely clustered but less variable, hence occupying a smaller perceptual space. Of importance for the evaluation of normalization is also that models patterned largely the same way across evaluations, indicating that the relative performance of each normalization account is the same regardless of the number of cues and size of vowel space. The best-performing centering accounts (C-CuRE) often achieve performance that is statistically indistinguishable from the best-performing standardization accounts (Lobanov). This is the case, in particular, when all five cues were considered and all 21 vowels were included in the categorization (see text footnote 8). Together with similar findings from research on consonants and supra-segmental categories (e.g., McMurray and Jongman, 2011; Apfelbaum et al., 2014; Toscano and McMurray, 2015; Kleinschmidt, 2019; Crinnion et al., 2020; Xie et al., 2021, 2023; Kulikov, 2022), this suggests that simple centering operations might be sufficient to maximize the benefits achievable by normalization. Given that these accounts involve computationally less complex operations, they might make up for a more plausible model of human perception, in contrast to standardizing accounts that involve more parameters for the listener to estimate.

The inclusion of both long and short vowels in the present study also motivated the inclusion of a temporal cue alongside the spectral cues that have been the focus of previous studies. Overall, including duration improves the model accuracy across evaluations. More specifically, when all vowels are considered and duration is included as cue, we see the largest increase in model performance across models, with the best-performing accounts moving from 57.9% recognition accuracy when only F1-F2 are considered, to 81.1% when all cues are considered, and chance being as low as 4.8%. This confirms the importance of duration as acoustic-perceptual cue for vowel quantity distinctions. It furthermore suggests that temporal cues, such as duration, are susceptible to normalization, and that vowel normalization mechanisms operate not only in frequency domains but also time domains. General purpose accounts that can take any type of cue as input, such as C-CuRE, would presumably have an advantage against vowel-specific accounts, even more so in languages with a systematic quantity distinction, such as Swedish. Future studies could investigate the relative advantage of general purpose accounts for languages that does not have a systematic quantity distinction, to see whether the results generalize.

In the remainder of the discussion, we first summarize some methodological considerations based on the present study, and then discuss limitations of our work, and how they can be addressed in future work.

### 4.1. Methodological considerations

In the present study, we employed Bayesian ideal observers to evaluate normalization accounts. Compared to the other perceptual models in Table 3, this has the advantage of reducing researchers' degrees of freedom. As mentioned in the introduction, support vector machines (Johnson and Sjerps, 2021), k-nearest neighbors (Carpenter and Govindarajan, 1993), or linear/logistic regression (Cole et al., 2010) would necessarily introduce additional degrees of freedom in the link from production to predicted perception. We emphasize, however, that other researchers can download the R markdown document for this article (which contains the R code for our models) from OSF and substitute any other perceptual model for the ideal observers to assess the extent to which our choice of computational framework affects our findings.

An auxiliary study presented in Supplementary Section 7 further demonstrates that the use of ideal observers also has advantages over the type of category variability/separability measure that has been used in many previous studies (cf. Table 2). We find that such separability indices can be dominated by a single cue, even when that cue is not particularly informative about category identity. This is unlikely to adequately reflect how listeners' perception would be affected by normalization. The ultimate reason for the deficiency of separability/variability indices is conceptual: the goal of speech perception is presumably not to reduce cue variability around the category mean but rather to improve speech recognition. These two goals are not the same (see also discussion in Barreda, 2020).

**Figure 7:**
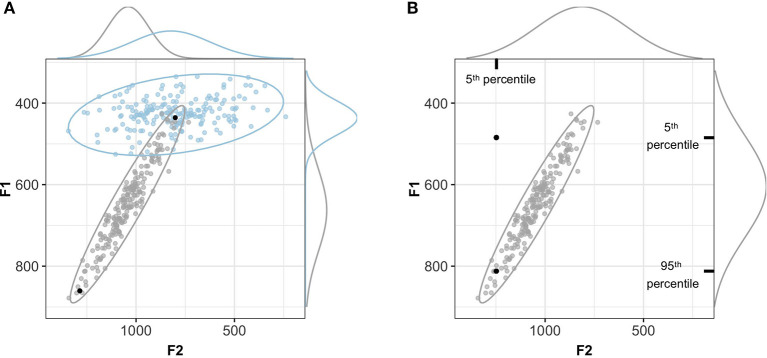
Using a perceptual model to evaluate normalization accounts avoids the pitfalls of separability/variability indices. **(A)** Two acoustic tokens that are equally far from the mean of one category can have radically different consequences for perception, depending on where the tokens fall relative to other categories. Under the hypothesis that the two black points are instances of the gray category, they would be attributed the same separability index but radically different probabilities given the joint distribution of cues relative to the other category in the space. Both points are on the 90% highest density interval isoline. **(B)** An acoustic token can be an improbable instance of a category if each cue is considered separately (the marginal densities along the sides of the plot), but highly probable if considered relative to the joint distribution of cues (the bivariate distribution indicated by the ellipse).

This is further illustrated in Figure 7A: by normalizing the support for a category by the support for all other categories (the denominator in Equations 1 and 2), ideal observers consider the perceptual consequences of an acoustic input *relative to all possible categories*. This means that a token that is relatively far away from its category mean does not necessarily result in low recognition accuracy. Rather, low recognition accuracy is only predicted if the relative position of the acoustic input in the acoustic-phonetic space makes it more probable that the input originated from another (unintended) category. This parallels human perception, and is illustrated in Figure 7A: e.g., while a more mid-fronted [ɑː] with high F1- and F2-values is atypical, human listeners are more likely to recognize it as a [ɑː] compared to a more high-back articulated, but equally atypical, [ɑː], presumably because the observed phonetics would be equally likely to occur if the talker intended a [oː]. Measures of between- vs. within-category variability like the separability index in the auxiliary study, however, have no means of directly capturing this.

Beyond this general advantage of perceptual models over variability/separability indices, the use of *multivariate* ideal observers allowed us to capture the *joint* effect of all cues. This captures that an input can be an improbable instance of a category based on one of its cue values but a probable instance given the values of all cues taken together. This is illustrated by Figure 7B.

Finally, we note three advantages of the five-fold cross-validation approach employed in the presented study. First and most obviously, cross-validation reduces the probability of over-fitting to the sample. Second, it provides researchers with an additional measure of uncertainty about the estimated performance of different normalization approaches. Although not discussed in the main text, we did compare the estimates across all five-folds, and found that both the mean estimates and their 95% CIs were stable across folds. This suggests that the present database is sufficiently large to yield stable results that generalize across folds. Third and related to the first point, cross-validation provides a more realistic—though still very crude—approximation of the problem that listeners face for normalization: the parameters used for normalization are not *a priori* known to listeners but rather must be incrementally inferred from the talker's speech input (Xie et al., 2023). More parameters—as required by more complex normalization accounts (e.g., Lobanov)—thus entail more estimation uncertainty, potentially reducing the effectiveness of such accounts for speech perception. By assessing the performance of normalization accounts on held-out test data, cross-validation begins to capture this downside of more complex accounts.

### 4.2. Limitations and future directions

Four limitations of the present study, three of which are shared with most previous work, deserve discussion. First, the present study compared normalization accounts against speech from only female talkers of one regional variety of Central Swedish (Stockholm Swedish). In contrast, many previous studies included data from talkers of different genders (e.g., Clopper, 2009; Cole et al., 2010; McMurray et al., 2011; Barreda, 2021), and sometimes from talkers of different ages (e.g., Hindle, 1978; Syrdal, 1985; Carpenter and Govindarajan, 1993; Flynn and Foulkes, 2011; Kohn and Farrington, 2012; Barreda and Nearey, 2018; Johnson and Sjerps, 2021) and/or language backgrounds (e.g., Disner, 1980; Adank et al., 2004; Escudero and Bion, 2007; Fabricius et al., 2009; Labov, 2010; Richter et al., 2017). Given that age, gender, etc. tend to affect formants (and other cues) beyond talker-variability, it is likely that the inclusion of more diverse talkers would increase the lack of invariance problem. For example, we would expect the ideal observers over unnormalized cues to achieve lower recognition performance if vowel productions from male talkers would be included in the data. In short, the models likely over-estimates the recognition accuracy that can be achieved for unnormalized cues if a more diverse range of talkers is considered.

What does this imply for our conclusions about the relative effect of normalization? To the extent that normalization successfully overcomes inter-talker variability that is caused by gender, age, and other social or physiological factors, we expect that the benefit of normalization accounts should show more clearly, relative to unnormalized cues. In this sense, the present study might *under*-estimate the relative benefits of normalization. Whether the *relative* performance of normalization accounts—i.e., the finding of primary interest to us—would differ if a more diverse range of talkers was considered is unclear. To the extent that vowel-specific accounts were originally developed specifically to eliminate physiological differences that are correlated with gender (as reviewed in, e.g., Johnson and Sjerps, 2021), it is theoretically possible that the high performance of general normalization accounts (e.g., C-CuRE, McMurray and Jongman, 2011) might not replicate when talkers of different genders are included. Future releases of the SwehVd database will contain data from male talkers, which will allow us or other researchers to revisit these questions.

Second, the present study aggregated acoustic-phonetic measurements taken at different points of the vowel (at 35, 50, and 65% into the vowel) into a single formant measurement. This follows previous comparisons of normalization accounts but is a simplifying assumption that should be revisited in future work. Formant dynamics carry important information for category distinctions (e.g., Nearey and Assmann, 1986; Hillenbrand and Nearey, 1999; Assmann and Katz, 2005), and are hypothesized to be of particular importance for some vowel distinctions in other varieties of Central Swedish (e.g., Kuronen, 2000). Prior to other consideration, this means that this study likely under-estimates the recognition accuracy that could be achieved even from unnormalized cues alone. It is an open question whether the findings of primary interest—the relative performance of different normalization accounts—would be affected if formant dynamics were considered. Some normalization accounts, for example, consider normalization of such formant dynamics to take place *after* basic formant normalization (but before the mapping of cues to category representations, S. Barreda, personal communication, 01/06/2023). Future work could employ SwehVd to compare ideal observers or other classification models while taking into consideration formant measurements throughout the vowel.

Third, we only considered *normalization* accounts. This, too, follows previous research on normalization but is potentially problematic. As mentioned in the introduction, it is now believed that at least three different mechanisms contribute to adaptive speech perception, including not only normalization but also changes in category representations and decision-making (for review, see Xie et al., 2023). This has consequences for research on normalization. For example, Xie et al. (2021) compared normalization accounts against the production of prosodic phrasing in L1-US English, while also considering alternative hypotheses about listeners' ability to adapt category representations. Xie and colleagues found that the effectiveness of cue normalization is substantially reduced if listeners can learn and maintain talker- or group-specific category representations (as assumed in some influential theories of speech perception, exemplar models, e.g., Johnson, 1997; Pierrehumbert, 2001; Bayesian ideal adaptors, Kleinschmidt and Jaeger, 2015). Xie and colleagues only considered two general types of normalization, and their focus was on the interpretation of prosodic signals. But their results call for caution in interpreting studies like the present that do not consider the possibility of talker-specific representations—an assumption shared with basically all previous work on vowel normalization.

Similarly, as mentioned in the introduction, we limited our evaluation to a single level of normalization (and combinations of perceptual transformations and a single level of normalization). Some proposals, however, assume multiple separate normalization steps. For example, some accounts hold that evolutionarily early mechanisms first transform spectral percepts into a phonetic space (e.g., uniform scaling accounts, Nearey, 1983; Barreda, 2020), on which additional subsequent normalization might operate. There is also evidence that speech perception combines aspects of intrinsic and extrinsic normalization (Johnson and Sjerps, 2021 review relevant evidence from brain imaging; early behavioral evidence is found in Nearey, 1989). The present study—like most existing evaluations—did not consider these possibilities (for exceptions, see e.g., Nearey and Assmann, 2007; Barreda, 2021).

Fourth and finally, we followed the majority of previous work and evaluated normalization accounts against *production* data. This is potentially problematic, especially when measures like category separability or reduced cross-talker variability in category means are used to evaluated normalization accounts (as in the auxiliary study in the SI and in many previous studies). These evaluations essentially assume that the goal of speech perception is to make the perceptual realizations of the same category by different talkers as similar as possible in the normalized space (for an in-depth critique, see Barreda, 2021). However, the goal of speech perception is presumably to reliably understand the meaning intended by the talker, and this aim does not necessarily entail perfect removal of cross-talker variability.

To some extent, our study addresses this potential issue by evaluating normalization accounts in terms of how well they predict the vowel category intended by the talker. However, if the goal is to explain human perception, the most informative evaluations of normalization accounts are arguably those that compare their predictions against *listeners'* behavior (for examples, see Nearey, 1989; Richter et al., 2017; Barreda, 2020, 2021; Xie et al., 2021). In short, approaches like that employed here take an important step away from the most misleading evaluation of normalization accounts in terms of reduced category variability/increased category separability. Ultimately, however, normalization accounts should be evaluated in terms of how well they predict listeners' perception, not talker's intention.

## Data availability statement

The dataset presented in this study can be found in an online repository (SwehVd: https://osf.io/ruxnb/). All analyses and visualization code can be found in a separate online repository (https://osf.io/zb8gx/).

## Ethics statement

Ethical review and approval was not required for the study on human participants in accordance with the local legislation and institutional requirements. The patients/participants provided their written informed consent to participate in this study.

## Author contributions

AP proposed project idea, designed SwehVd materials, recorded and annotated vowel productions, coded cue extraction, coded data analyses and visualization with guidance from TJ and wrote the initial draft of the manuscript. Both authors jointly developed the conceptual approach and contributed to revisions. Both authors contributed to the article and approved the submitted version.

## Footnotes

^1^We note that this argument assumes that listeners' category representations pool experiences across talkers into a single talker-independent model. Such talker-independent category representations are assumed in many influential models of spoken word recognition (e.g., McClelland and Elman, 1986; Luce and Pisoni, 1998; Norris and McQueen, 2008). While talker-independent representations might be a simplifying assumption for some of these theories, this assumption has persisted for decades (e.g., Magnuson et al., 2020; ten Bosch et al., 2022). Exceptions include exemplar accounts (e.g., Johnson, 1997; Pierrehumbert, 2001) and the Bayesian ideal adaptor account (Kleinschmidt and Jaeger, 2015). Importantly, it is an unresolved question whether—or for which cues and phonetic contrasts—listeners maintain talker-specific *category* representations (for findings and discussion, see Kraljic and Samuel, 2007; Kleinschmidt and Jaeger, 2015; Kleinschmidt, 2019; Xie et al., 2021). Here, we follow previous work and compare the effectiveness of normalization under the assumption of talker-independent category representations.

^2^Miller's formant-ratio account (Miller, 1989) is technically a hybrid approach: the first formant (F1) is normalized with regard to an extrinsic sensory reference (based on the average F0 across segments); subsequent formants are (intrinsicly) normalized using the normalized lower formants on the same vowel segment.

^3^Here we group accounts based on their computational complexity (the number of parameters listeners are assumed to estimate). For example, we group Nearey1 and Nearey2 with the centering accounts because they require estimation of only cue means. However, since these accounts perform centering over log-transformed Hz, they can also be considered as a form of functionally constrained standardization in non-log space (Barreda and Nearey, 2018).

^4^This reflects the mainstream analytical position in present-day Swedish phonology. The opposite position, distinctive vowel quantity, has also been proposed (e.g., Linell, 1978, 1979; Schaeffler, 2005).

^5^While this is the approach most commonly employed in the literature, it has the potential downside that co-articulation might affect formant values at the measurement points differently for long and short vowels (since the long and short vowels differ in overall duration). An alternative approach would be to extract formants at fixed durations (e.g., 30 ms) after the vowel onset and before the vowel offset. Since our findings do not indicate any systematic differences in the performance of normalization accounts between long and short vowels, we do not consider this issue further here.

^6^Some of these studies additionally included F0 (Syrdal, 1985; Nearey, 1989; Adank et al., 2004). However, since F0 is a cue that can display substantial cross-talker variability without directly contributing much information to vowel categorization (recall Figure 4), we decided to add only F3 to F1-F2 in the second evaluation.

^7^Human perception is affected by an additional source of uncertainty beyond category variability: perceptual noise (for review, see Feldman et al., 2009). Since the present study compares the *relative* recognition accuracy of different normalization accounts, it is not immediately obvious how the inclusion of noise could affect our results. To avoid additional researchers degrees of freedom—such as the decision as to which acoustic or perceptual space (Hz, Mel, Bark, etc.) perceptual noise is additive in—we do not model the perceptual consequences of noise.

^8^Indeed, when all five cues are considered for the categorization of all 21 short and long vowels, the best centering account perform numerically better (81.1%) than Lobanov normalization (80.5%). This is, however, an artifact of our decision to only center vowel duration—the primary cue to vowel quantity—for the C-CuRE model. Separate modeling not shown here confirmed that Lobanov normalizaton achieves the same recognition accuracy as the C-CuRE models when duration is centered and combined with Lobanov-normalized formants (82.1%, 95%-CI: 79.2-84.9%).
