# Supplementary material for: Evaluating normalization accounts against the dense vowel space of Central Swedish
Source: Front Psychol. 2023 Jun 21;14:1165742. doi: 10.3389/fpsyg.2023.1165742 (PMC10322199; doi:10.3389/fpsyg.2023.1165742)
Supplement: Supplementary file 1 [file Data_Sheet_1.pdf]

Supplementary Material for “Evaluating normalization accounts against the dense vowel space of Central Swedish”

Anna Persson and T. Florian Jaeger

Both the main text and these supplementary information (SI) are derived from the same R markdown document available via OSF at `https://osf.io/zb8gx/`.

1 REQUIRED SOFTWARE

The document was compiled using `knitr` (Xie, 2021) in RStudio with R:

```
               _
platform       aarch64-apple-darwin20
arch           aarch64
os             darwin20
system         aarch64, darwin20
status
major          4
minor          3.0
year           2023
month          04
day            21
svn rev        84292
language       R
version.string R version 4.3.0 (2023-04-21)
nickname       Already Tomorrow
```

We used the following R packages to create this document: R (Version 4.3.0; R Core Team, 2021b) and the R-packages *assertthat* (Version 0.2.1; Wickham, 2019a), *cowplot* (Version 1.1.1; Wilke, 2020), *data.table* (Version 1.14.8; Dowle and Srinivasan, 2021), *dplyr* (Version 1.1.2; Wickham et al., 2021a), *ggh4x* (Version 0.2.4; van den Brand, 2023), *ggplot2* (Version 3.4.2; Wickham, 2016), *gridExtra* (Version 0.2.3; Auguie & Antonov, 2017), *LaplacesDemon* (Version 16.1.6; Statisticat and LLC., 2021), *latexdiffr* (Version 0.1.0; Hugh-Jones, 2021), *linguisticsdown* (Version 1.2.0; Liao, 2019), *lme4* (Version 1.1.33; Bates et al., 2015), *magick* (Ooms, 2021), *magrittr* (Version 2.0.3; Bache and Wickham, 2020), *Matrix* (Version 1.5.4; Bates and Maechler, 2021), *modelr* (Version 0.1.11; Wickham, 2020), *MVBeliefUpdatr* (Version 0.0.1.2; Kleinschmidt and Jaeger, 2015b), *nplyr* (Version 0.2.0; Rieke & Elias, 2023), *papaja* (Version 0.1.1.9001; Aust and Barth, 2020), *processx* (Version 3.8.1; Csárdi and Chang, 2021), *purrr* (Version 1.0.1; Henry and Wickham, 2020), *Rcpp* (Version 1.0.10; Eddelbuettel and François, 2011; Eddelbuettel and Balamuta, 2018), *readr* (Version 2.1.4; Wickham et al., 2021b), *rlang* (Version 1.1.1; Henry and Wickham, 2021), *stringr* (Version 1.5.0; Wickham, 2019b), *tibble* (Version 3.2.1; Müller and Wickham, 2021), *tidyr* (Version 1.3.0; Wickham, 2021b), *tidyverse* (Version 2.0.0; Wickham et al., 2019), *tinylabels* (Version 0.2.3; Barth, 2022), and *tufte* (Version 0.12; Xie and Allaire, 2022).

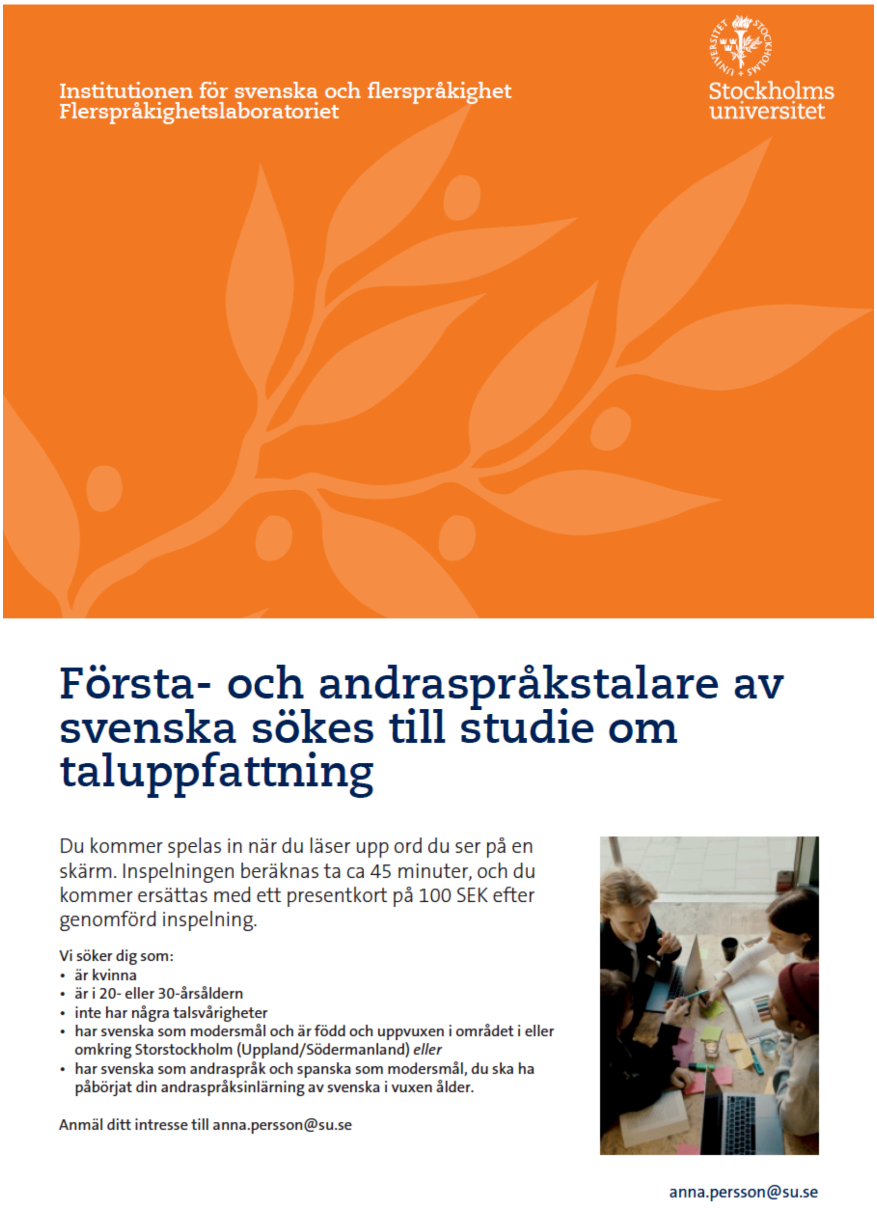

Figure S1: Example flyer for recruiting Stockholm Swedish talkers for recording of the SwehVd database.

2 ADDITIONAL INFORMATION ABOUT THE SWEHVD DATABASEE

2.1 Participant recruitment

Participants were recruited through word-of-mouth, flyers at Stockholm University Campus, and online channels (accindi.se). Figure S1 is an example of flyers distributed at Stockholm University Campus. The flyer gives information on criteria for participation, recording procedure, reimbursement and contact information to experimenter (first author).

2.2 Word list

Word list with all target and filler words, recorded by all talkers in the SwehVd database.

Table S1. Words recorded by the female talkers of Stockholm Swedish for the SwehVd database

Target words	Vowel IPA
hid	[iː]
hidd	[ɪ]
hyd	[yː]
hydd	[ʏ]
hed	[eː]
hedd	[ɛ]
häd	[ɛː]
hädd	[ɛ]
härd	[æː]
härr	[æ]
höd	[øː]
hödd	[ø]
hörd	[œː]
hörr	[œ]
hud	[ʉː]
hudd	[ɵ]
hod	[uː]
hodd	[ʊ]
håd	[oː]
hådd	[ɔ]
had	[ɑː]
hadd	[a]

Filler words	
titt	tand
damm	dipp
tå	buss
bål	ding
dill	porr
tugga	mitt
mat	dopp
norr	tal
must	namn
pil	pall
dina	bar
biff	till
Tina	mål
borr	Nina
dal	då
Pål	nick
nunna	ditt
mil	dugga
ting	mall
ball	bil
piff	par
tipp	morr
puss	nav
topp	nå

2.3 Unanticipated challenges during recording (and how they were addressed)]

In a small-scale pilot preceding recordings, the expected transparency of the orthography for eliciting the long and short vowels was confirmed by three native talkers and one non-native talker of Swedish (these talkers did not participate in the study). However, *hodd* [ʊ] and *hod* [uː] sometimes elicited [ɔ].[S1] We therefore decided to add instructions to the participants for these two words. When *hod* or *hodd* appeared on screen, a written guide indicating the target vowel appeared below the word in smaller font size: "hod som i hot", "hodd som i hosta", with *hot* and *hosta* being real Swedish words containing [uː] and [ʊ], respectively.[S2] Whenever the experimenter noticed that the pronunciations clearly targeted another vowel, recordings were stopped and participants were reminded to carefully read the guide. Despite our recording instructions, five of the talkers rarely ever produced the targeted [ʊ] vowel. Instead, they often mispronounced the vowel, hence they are not included in the subsetted SwehVd we use in this study.

2.4 Neutralization of hedd and hädd

For the vast majority of talkers, *hädd* productions elicited the same vowel as *hedd* (see Figure S2). This confirms the common assumption that the short allophone to /e/ neutralizes with the short allophone to [ɛ] in Central Swedish.

[S1] The difficulty for some native talkers to produce [ʊ] when reading *hodd* might be due to frequency effects. Forms with stressed [ʊ] are rare in the Swedish language, and phonotactically similar words are most often pronounced as [ɔ] (see e.g., Riad, 2014).

[S2] English translations: "hod as in threat"(phonologically [uː]), "hodd as in cough"(phonologically [ʊ]).

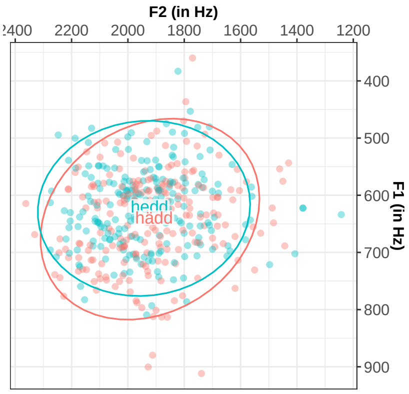

Figure S2: The *hedd* and *hädd* words in the SwehVd vowel data in unnormalized F1-F2 space. Points show recordings of the *hedd* and *hädd* words ([ɛ]) by the 24 female native talkers in the database, averaged across the five measurement points within each vowel segment. Word labels indicate word means across talkers. Since *hädd* and *hedd* resulted in the same allophone, we exclude *hädd* from this and all other visualizations below. This facilitates comparison of, for example, densities across vowels.

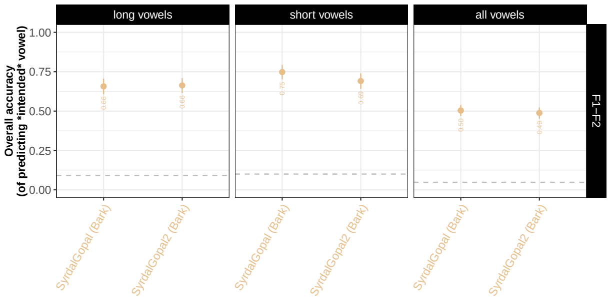

Figure S3: Predicted recognition accuracy of ideal observer under two versions of the Syrdal & Gopal (1986) account for long vowels, short vowels, and long and short vowels together, shown for the F1-F2 cue combination. Labels indicate mean across the five test folds. Intervals show average bootstrapped 95% confidence intervals across the test folds. The dashed horizontal line indicates chance (different across columns because of the different number of long and short vowels).

3 EVALUATION OF IMPLEMENTATIONS OF SYRDAL & GOPAL'S (1986) SECOND DIMENSION

For the second dimension, distinguishing between front and back vowels, Syrdal and Gopal (1986) evaluates two different bark-difference measures: F2-F1 and F3-F2. Previous studies had concluded that F2-F1 distinguishes between all Swedish vowels (Fant, 1983), however, in Syrdal and Gopal (1986)'s evaluation of American English, the F3-F2 dimension provided a better fit. Given that there seems to be language specific effects concerning Syrdal and Gopal (1986)'s second dimension (e.g., Adank, 2003), here we compare the two difference measures for the vowels in the SwehVd database.

Figure S3 displays the categorization accuracy for models trained on normalized data under the two implementations of the Syrdal & Gopal account. The first version uses the F2-F1 bark-difference metric for the second dimension, whereas the second version (labelled *SyrdalGopal2 (Bark)*) implements the second dimension as suggested by Syrdal and Gopal (1986), F3-F2. As

evident from Figure S3, the first implementation performs better at separating categories in the SwehVd data.

Figure S4 displays the separability index for the two implementations. Mirroring the results from categorization accuracy, the first implementation using F2-F1 for the second dimension, outperforms the implementation using F3-F2 bark-difference measure, replicating Fant (1983). These results taken together indicate that the F2-F1 implementation is more suitable for the materials used here, we therefore decided to use the first implementation throughout this paper.

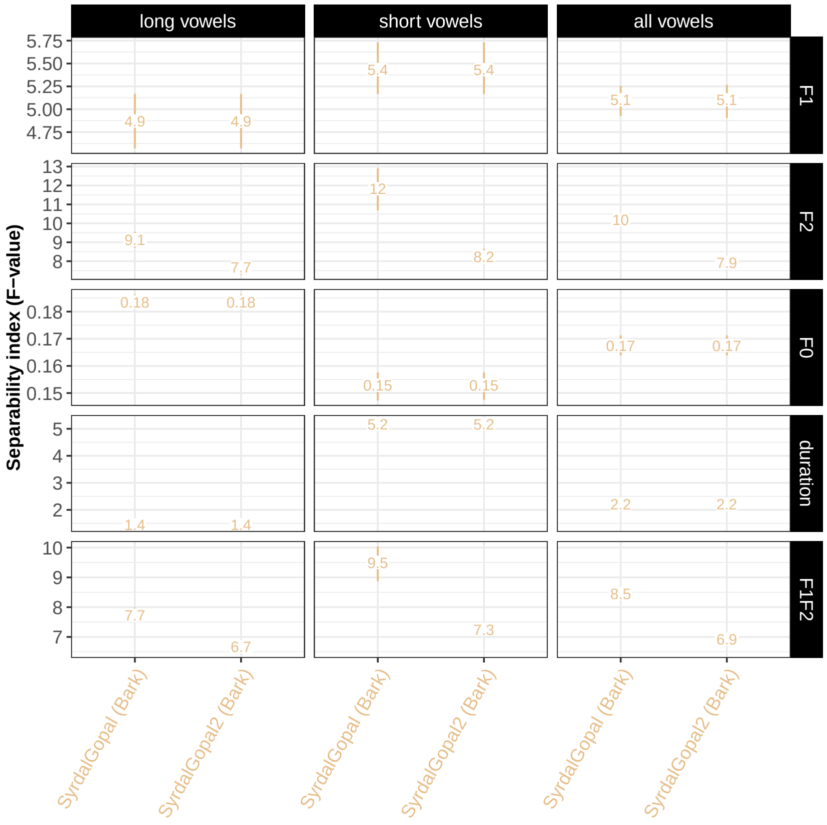

Figure S4: Separability indices of the two versions of the Syrdal & Gopal (1986) account for long vowels, short vowels, and long and short vowels together, shown for four of the five cues considered in this study and the combined F1-F2. Labels indicate mean across the five test folds. Intervals show average bootstrapped 95% confidence intervals across the test folds. Note that the ranges of the y-axes varies across plots.

4 VISUALIZING THE DISTRIBUTION OF VOWEL PRODUCTIONS

Figures S5 and S6 show the Central Swedish vowels, after applying the 15 different normalization accounts. For this purpose, we focus on F1 and F2 only. In Section Correlation matrices for all normalization accounts, we plot pairwise correlation plots of all cues for all different normalization accounts we compare.

Visual inspection suggests a few initial observations. The most striking difference is perhaps between intrinsic normalization accounts (Syrdal and Gopal, 1986; Miller, 1989) and all other approaches, though it is not immediately visually obvious which type of approach achieves better

separability. Second, transforming the vowels to a different perceptual scale does not seem to affect the vowel distributions much, besides a minor decrease in category variance for some of the vowels. Some transformations bring the vowel categories closer together, towards the center of the vowel space, e.g., ERB and semitones. Third, centering formants by subtracting each talkers’ mean (McMurray and Jongman, 2011; Nearey, 1978) reduces some of the category variance, and as a result, increases the category separability. Transforming the vowel data into different scales prior to centering also seems to further improve separability (compare e.g., C-CuRE (Hz) and C-CuRE (semitones)). Overall, the top two performing accounts across the long and short vowels appear to be Lobanov (1971) and Nearey (1978). However, even for the best performing normalization accounts, there is still considerable category overlap. This involves some of the high long vowels, and some of the mid-center short vowels. This highlights the need to more systematically quantify the effects of normalization, as we do in this study.

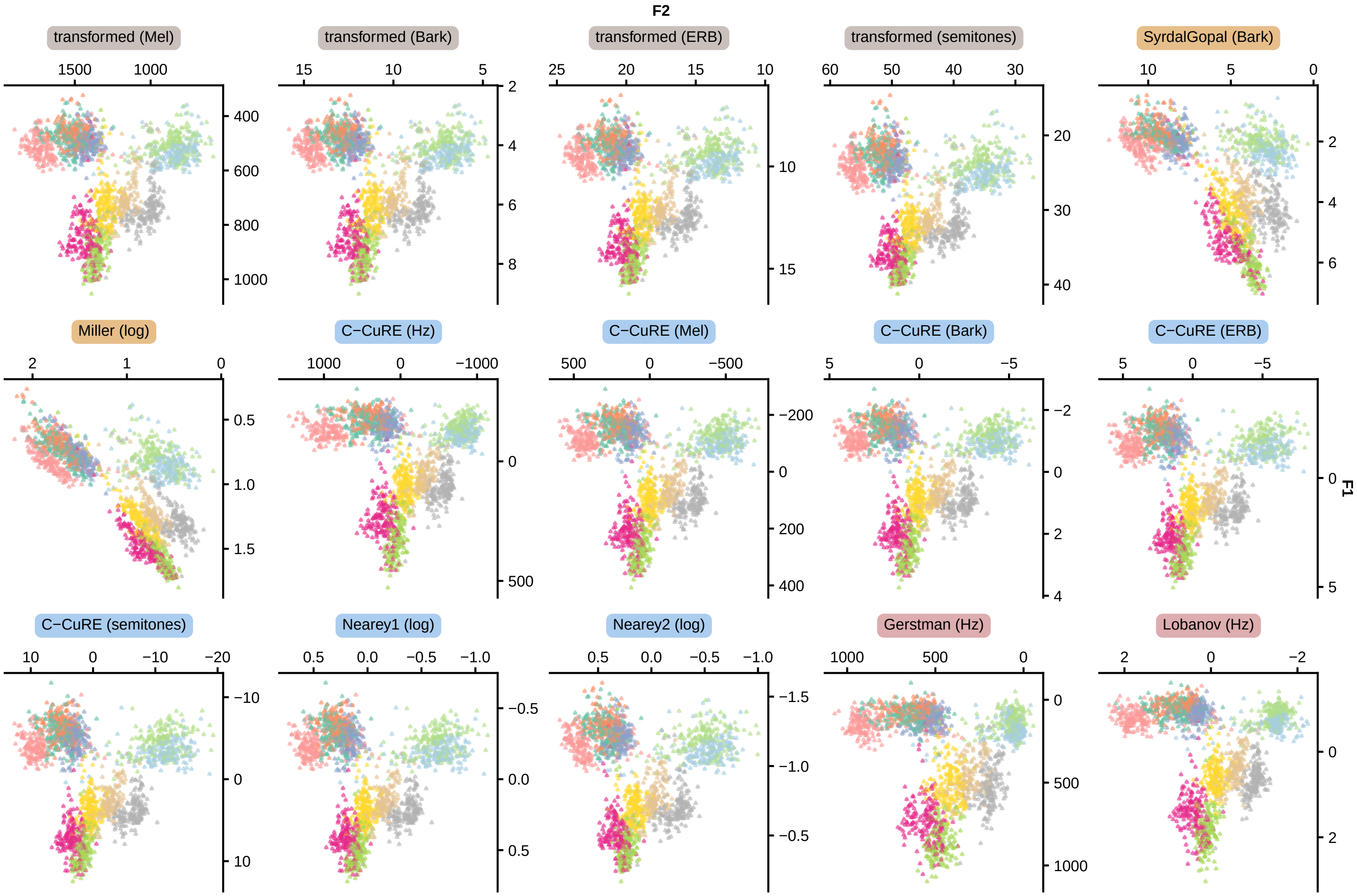

Figure S5: The 11 long vowels of Central Swedish when F1 and F2 are transformed into a perceptual scale (**grey**), intrinsically normalized (**yellow**), or extrinsically normalized through centering (**blue**) or standardizing (**purple**). Each point corresponds to one recording, averaged across the five measurement points within each vowel segment. Each panel combines the data from all five test folds.

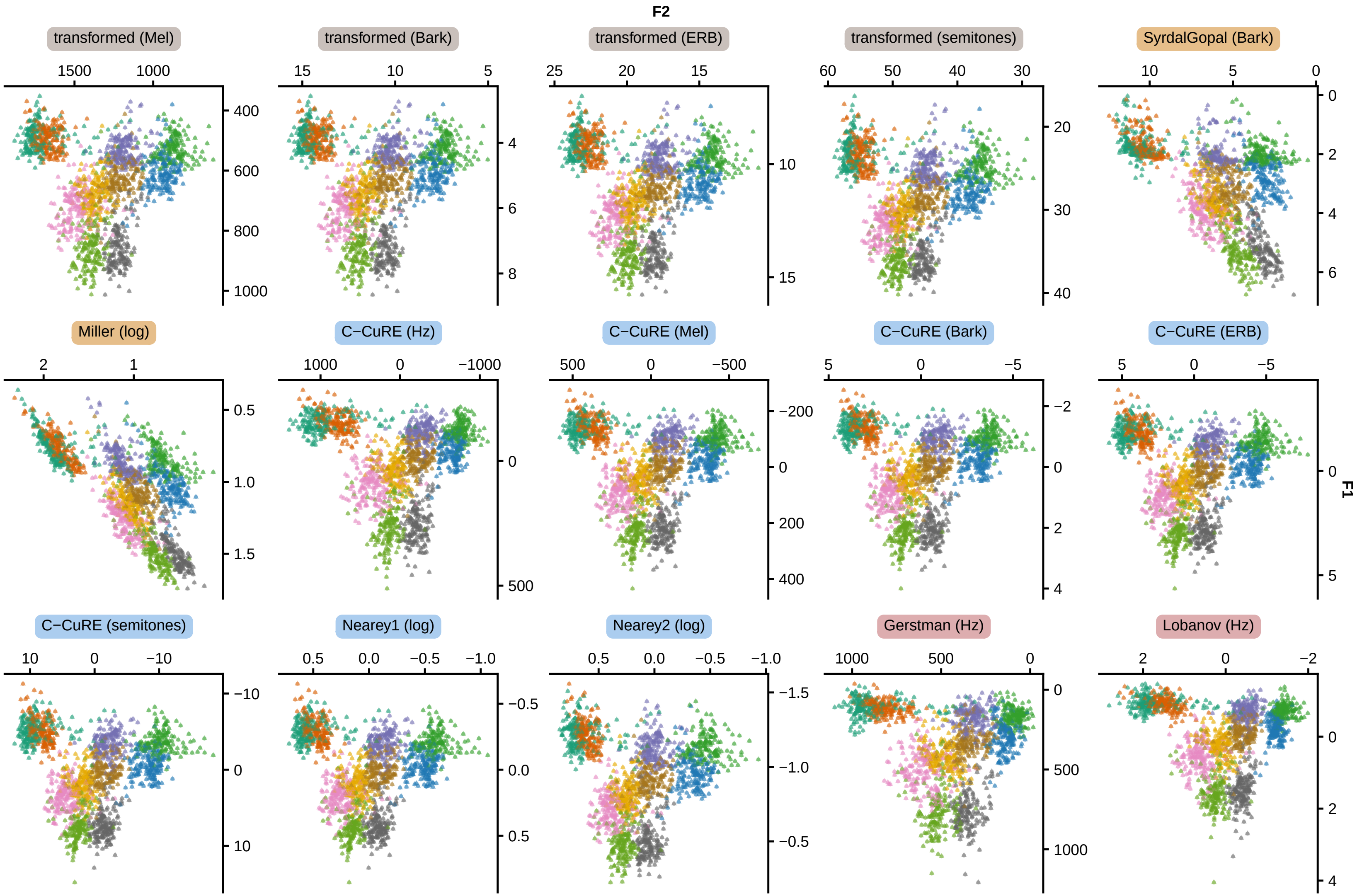

Figure S6: The 10 short vowels of Central Swedish when F1 and F2 are transformed into a perceptual scale (**grey**), intrinsically normalized (**yellow**), or extrinsically normalized through centering (**blue**) or standardizing (**purple**). Each point corresponds to one recording, averaged across the five measurement points within each vowel segment. Each panel combines the data from all five test folds.

5 CUE CORRELATION MATRICES FOR ALL NORMALIZATION ACCOUNTS

Figures S7 - S21 show the cue correlation matrices for the SwehVd vowel data for all 15 normalization accounts.

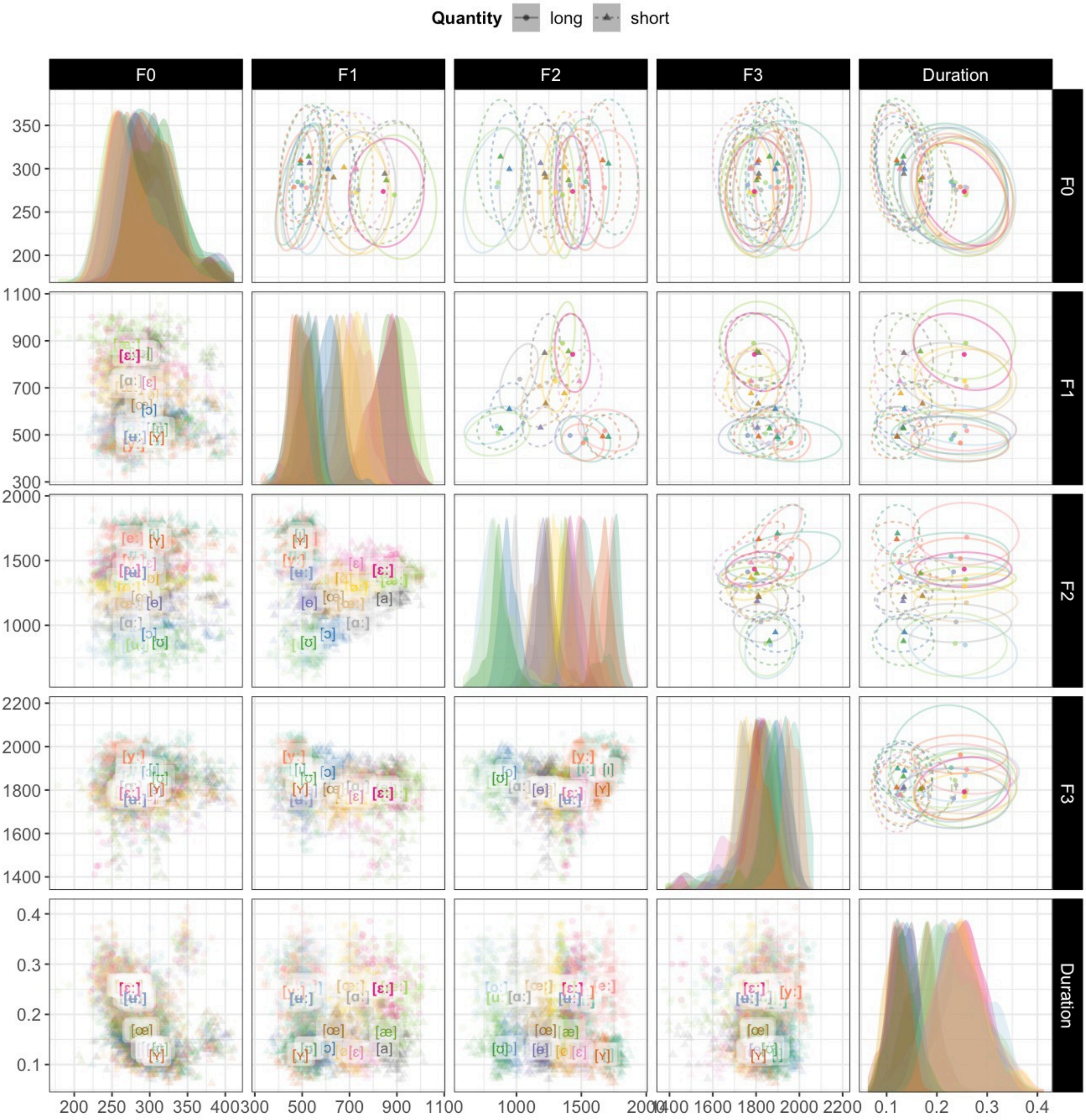

Figure S7: The SwehVd vowel data in Mel space. Points show repetitions of each of the 21 Central Swedish vowels by 16 female native talkers in the database in F0-F3 and vowel duration cue space. Vowel labels indicate category means across talkers. Long vowels are boldfaced. Ellipses show bivariate Gaussian 95% confidence interval of category means.

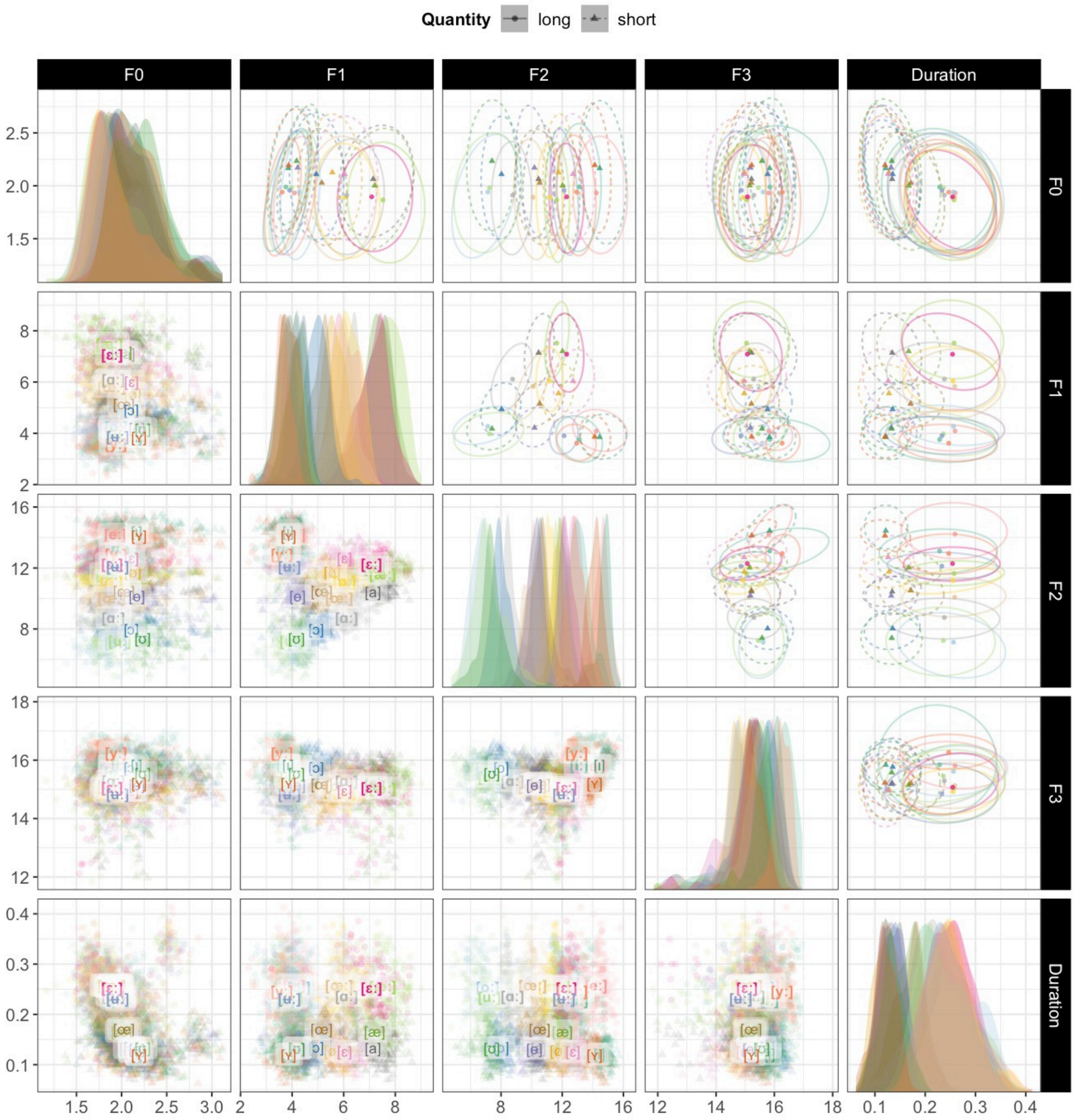

Figure S8: The SwehVd vowel data in Bark space. Points show repetitions of each of the 21 Central Swedish vowels by 16 female native talkers in the database in F0-F3 and vowel duration cue space. Vowel labels indicate category means across talkers. Long vowels are boldfaced. Ellipses show bivariate Gaussian 95% confidence interval of category means.

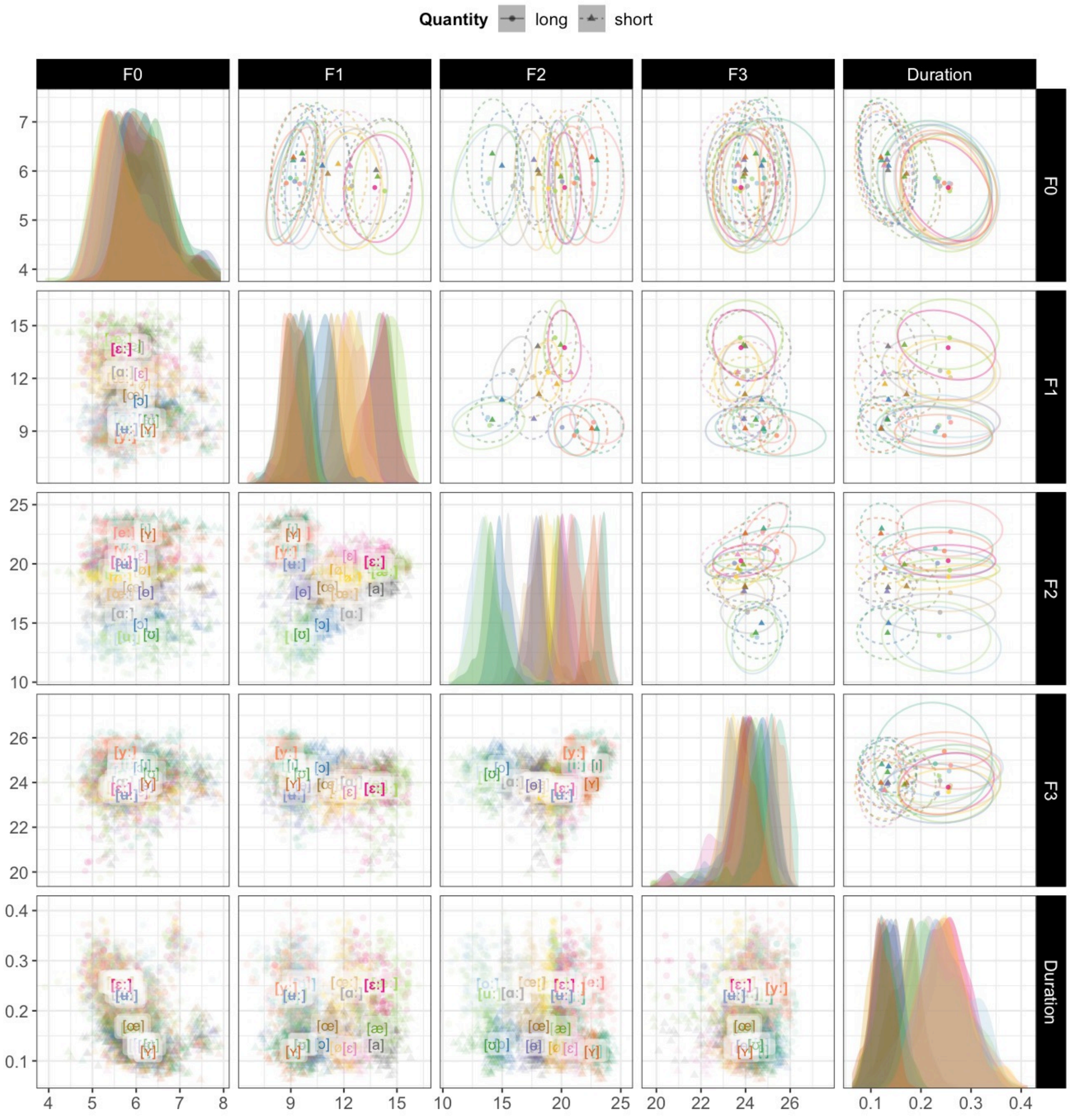

Figure S9: The SwehVd vowel data in ERB space. Points show repetitions of each of the 21 Central Swedish vowels by 16 female native talkers in the database in F0-F3 and vowel duration cue space. Vowel labels indicate category means across talkers. Long vowels are boldfaced. Ellipses show bivariate Gaussian 95% confidence interval of category means.

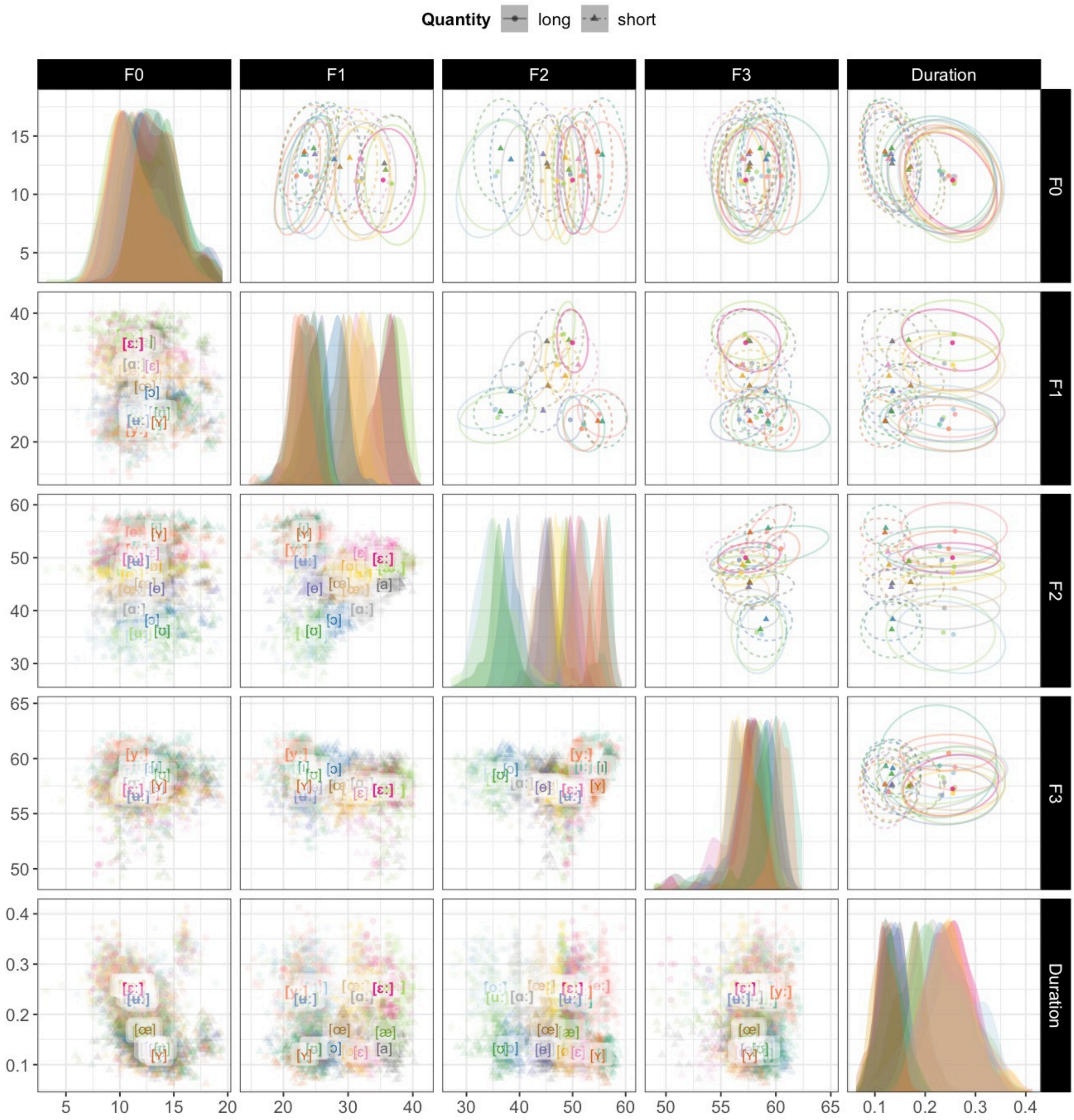

Figure S10: The SwehVd vowel data in semitones space. Points show repetitions of each of the 21 Central Swedish vowels by 16 female native talkers in the database in F0-F3 and vowel duration cue space. Vowel labels indicate category means across talkers. Long vowels are boldfaced. Ellipses show bivariate Gaussian 95% confidence interval of category means.

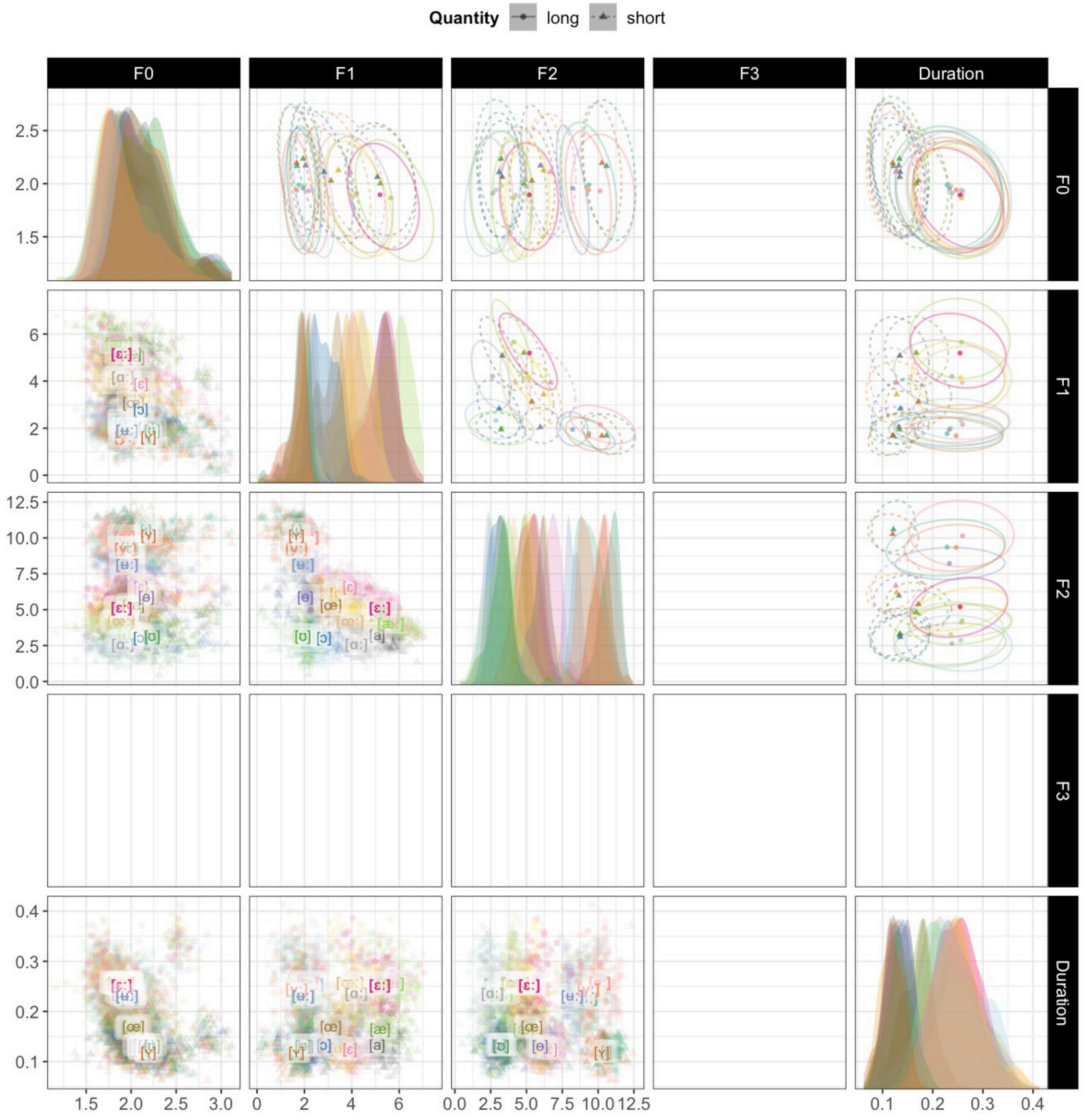

Figure S11: The SwehVd vowel data in SyrdalGopal (Bark) space. Points show repetitions of each of the 21 Central Swedish vowels by 16 female native talkers in the database in F0-F3 and vowel duration cue space. Vowel labels indicate category means across talkers. Long vowels are boldfaced. Ellipses show bivariate Gaussian 95% confidence interval of category means.

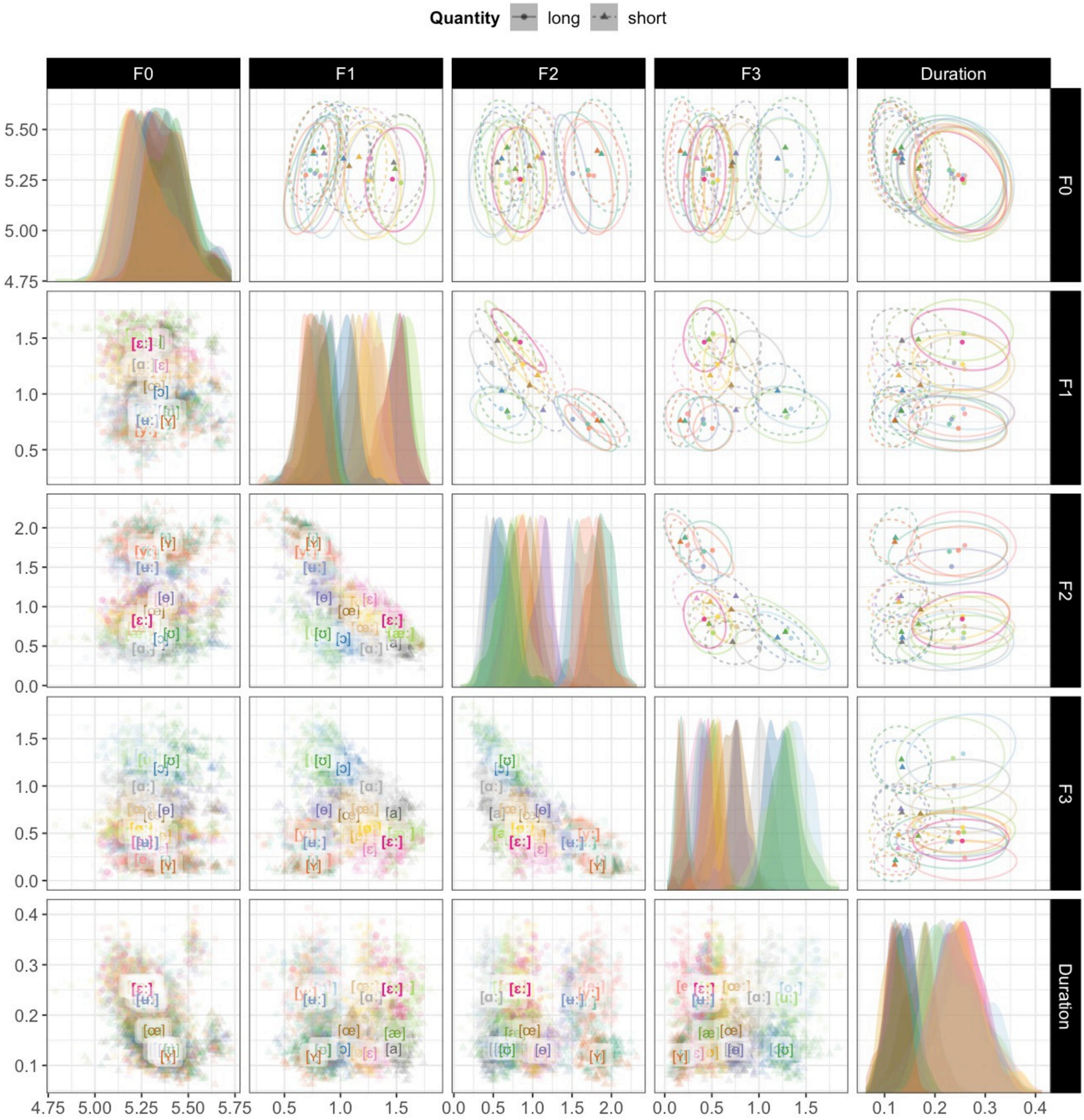

Figure S12: The SwehVd vowel data in Miller (log) space. Points show repetitions of each of the 21 Central Swedish vowels by 16 female native talkers in the database in F0-F3 and vowel duration cue space. Vowel labels indicate category means across talkers. Long vowels are boldfaced. Ellipses show bivariate Gaussian 95% confidence interval of category means.

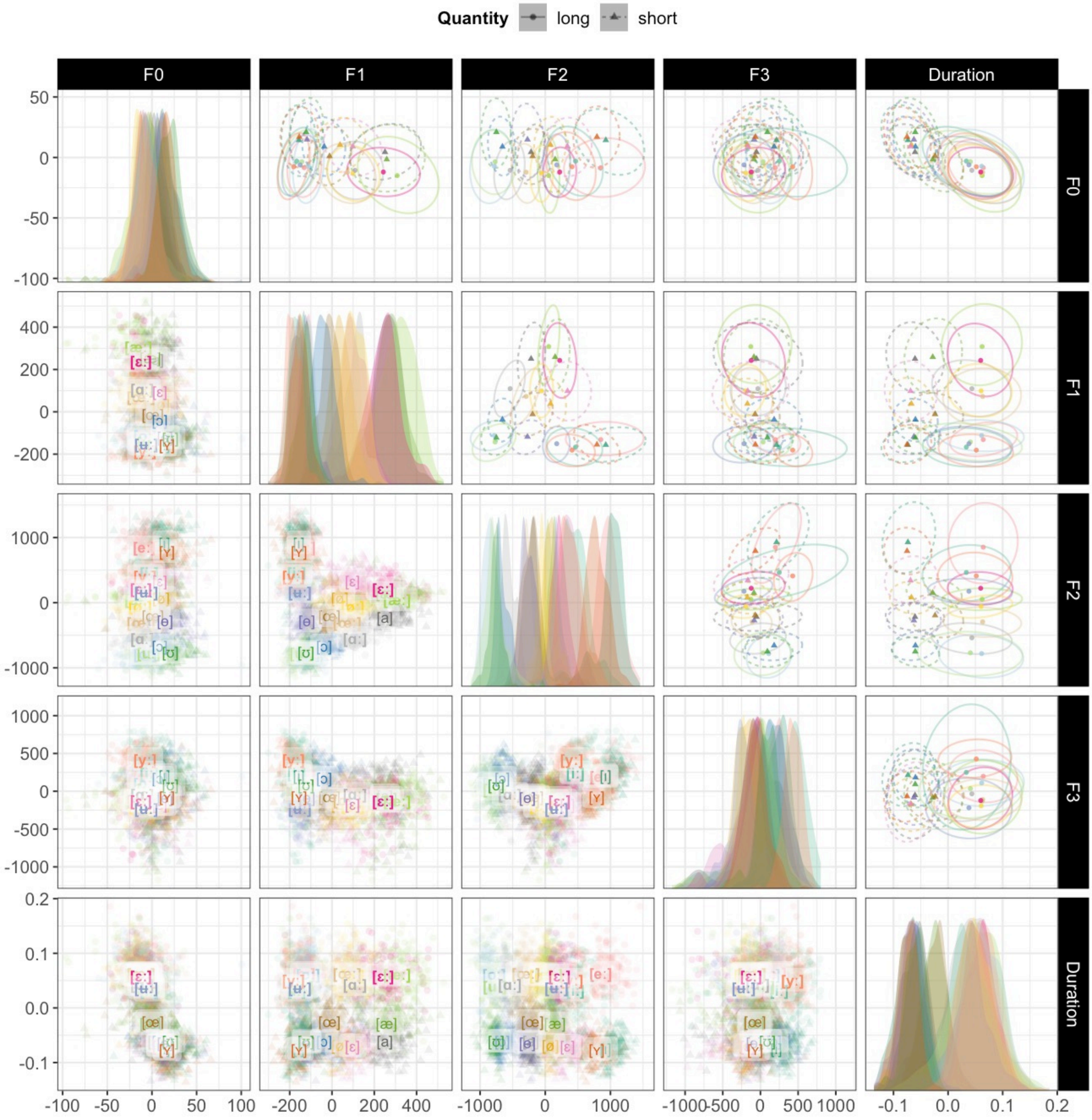

Figure S13: The SwehVd vowel data in C-CuRE Hz space. Points show repetitions of each of the 21 Central Swedish vowels by 16 female native talkers in the database in F0-F3 and vowel duration cue space. Vowel labels indicate category means across talkers. Long vowels are boldfaced. Ellipses show bivariate Gaussian 95% confidence interval of category means.

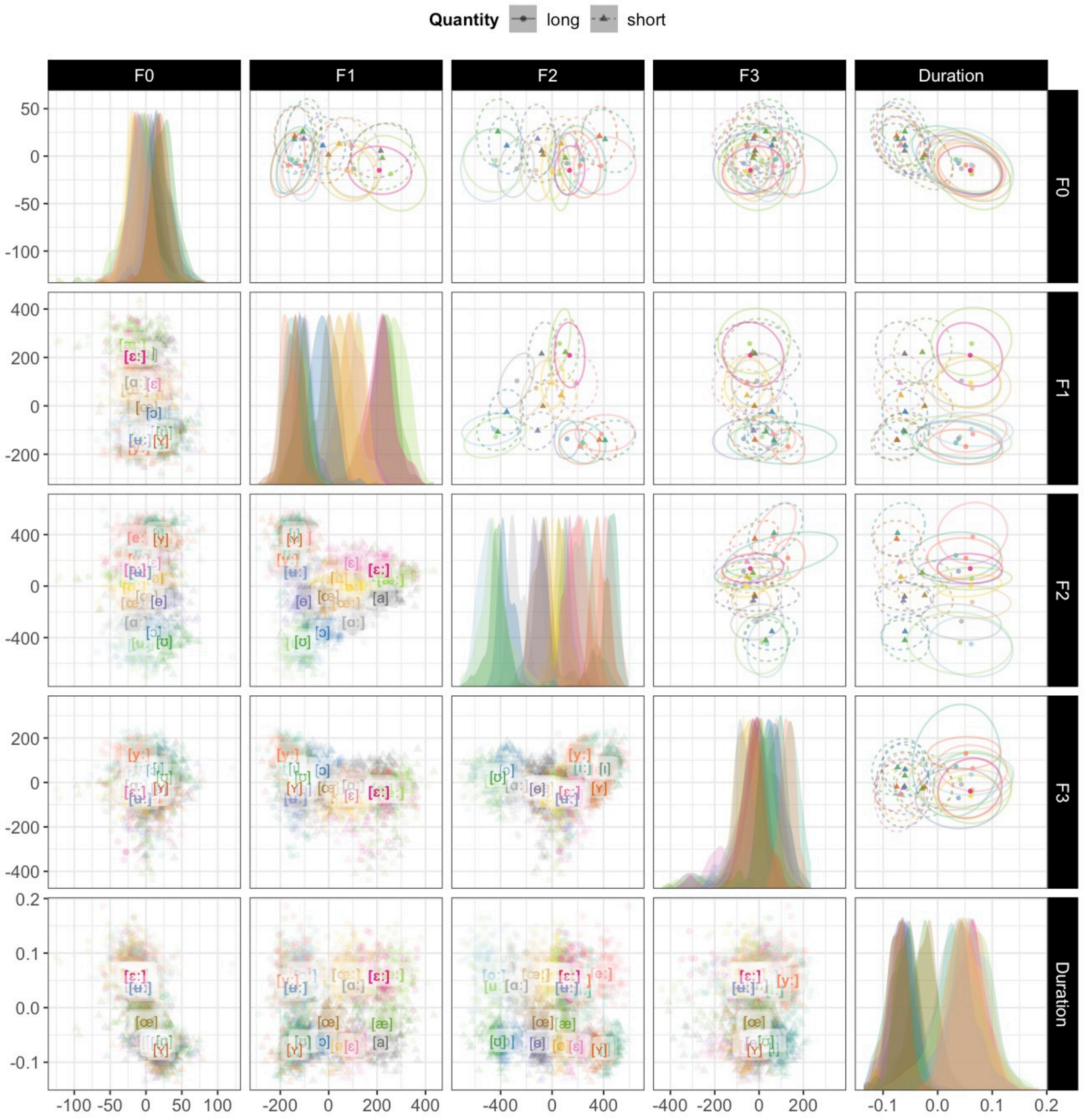

Figure S14: The SwehVd vowel data in C-CuRE Mel space. Points show repetitions of each of the 21 Central Swedish vowels by 16 female native talkers in the database in F0-F3 and vowel duration cue space. Vowel labels indicate category means across talkers. Long vowels are boldfaced. Ellipses show bivariate Gaussian 95% confidence interval of category means.

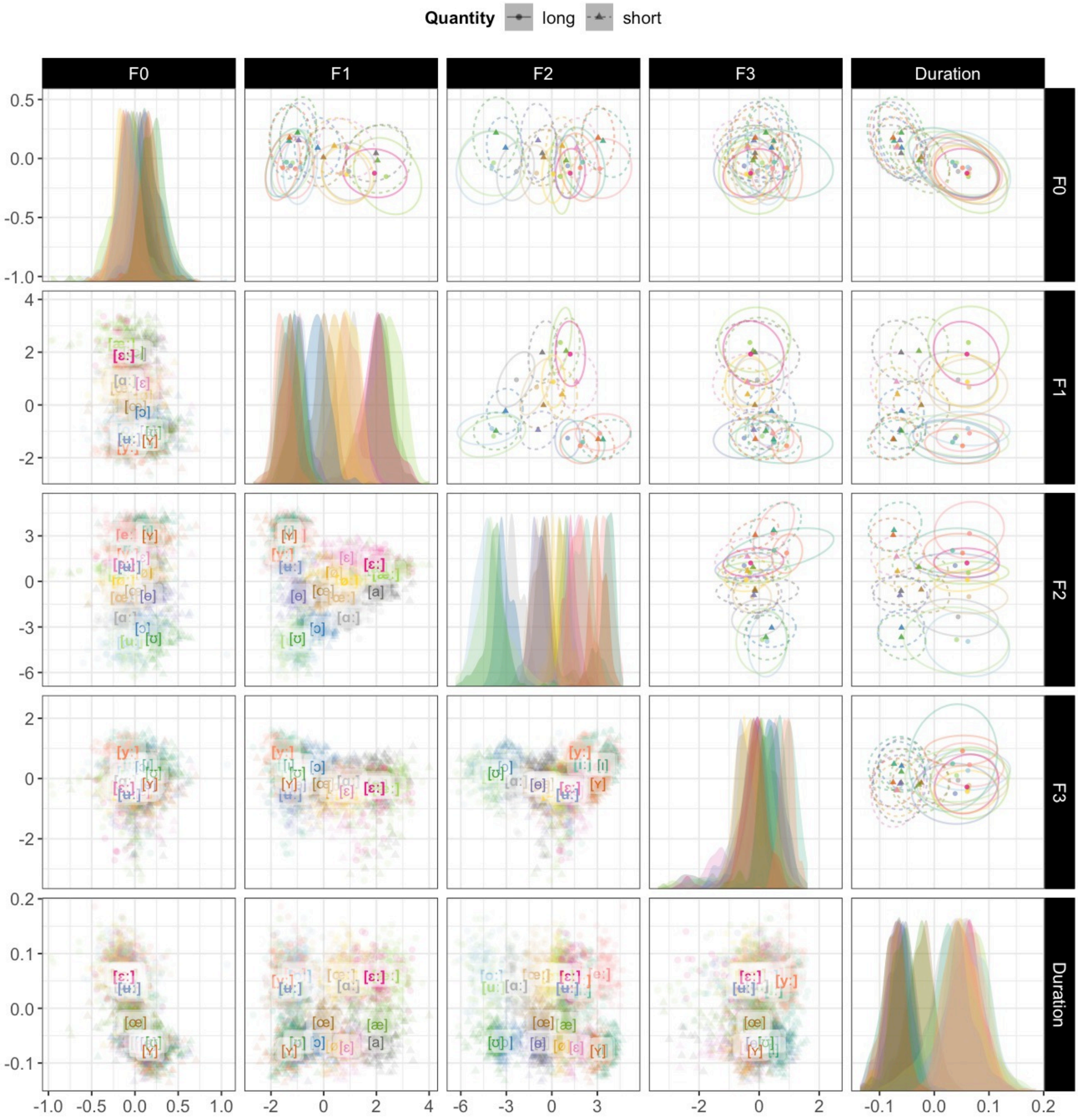

Figure S15: The SwehVd vowel data in C-CuRE Bark space. Points show repetitions of each of the 21 Central Swedish vowels by 16 female native talkers in the database in F0-F3 and vowel duration cue space. Vowel labels indicate category means across talkers. Long vowels are boldfaced. Ellipses show bivariate Gaussian 95% confidence interval of category means.

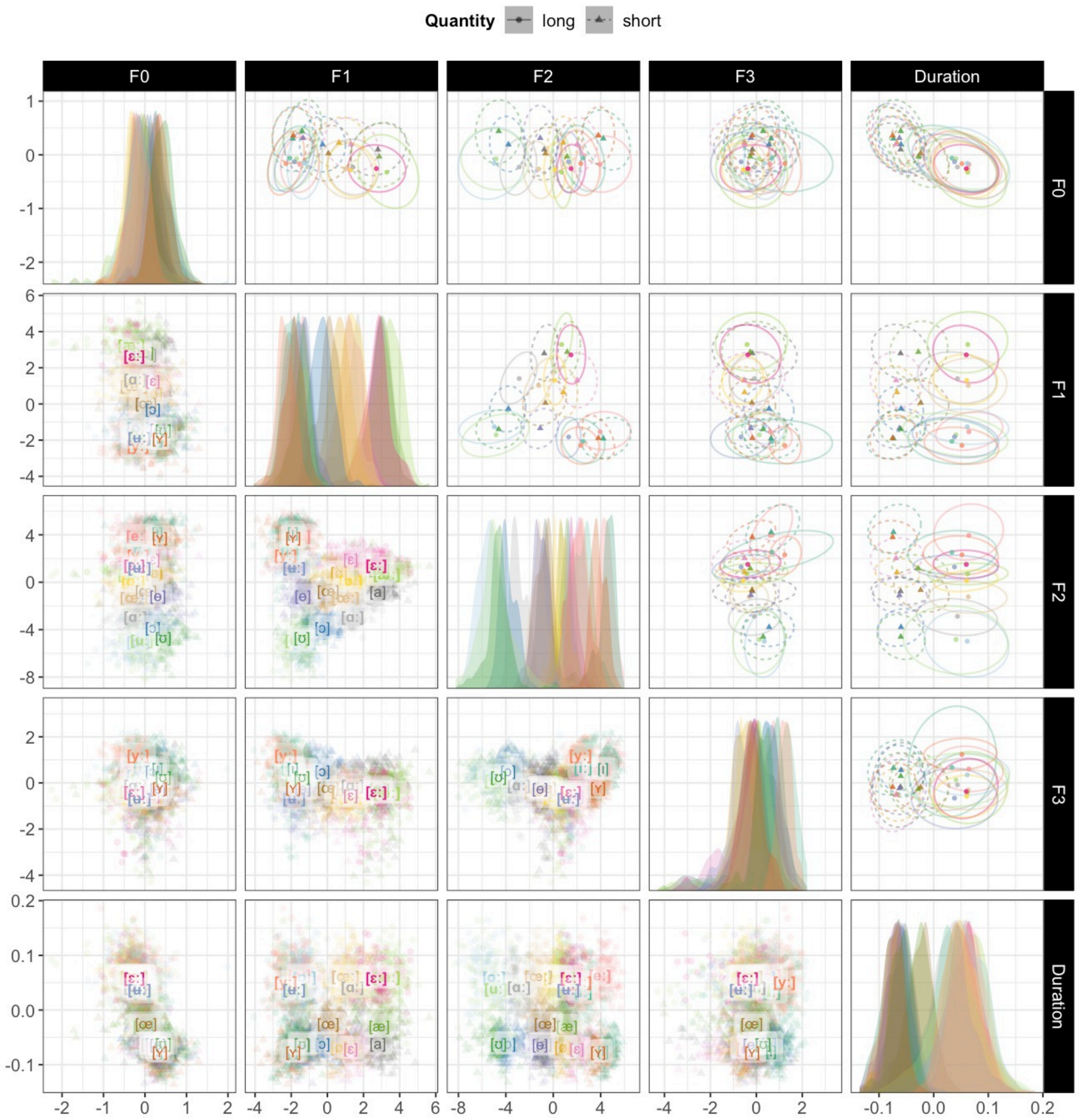

Figure S16: The SwehVd vowel data in C-CuRE ERB space. Points show repetitions of each of the 21 Central Swedish vowels by 16 female native talkers in the database in F0-F3 and vowel duration cue space. Vowel labels indicate category means across talkers. Long vowels are boldfaced. Ellipses show bivariate Gaussian 95% confidence interval of category means.

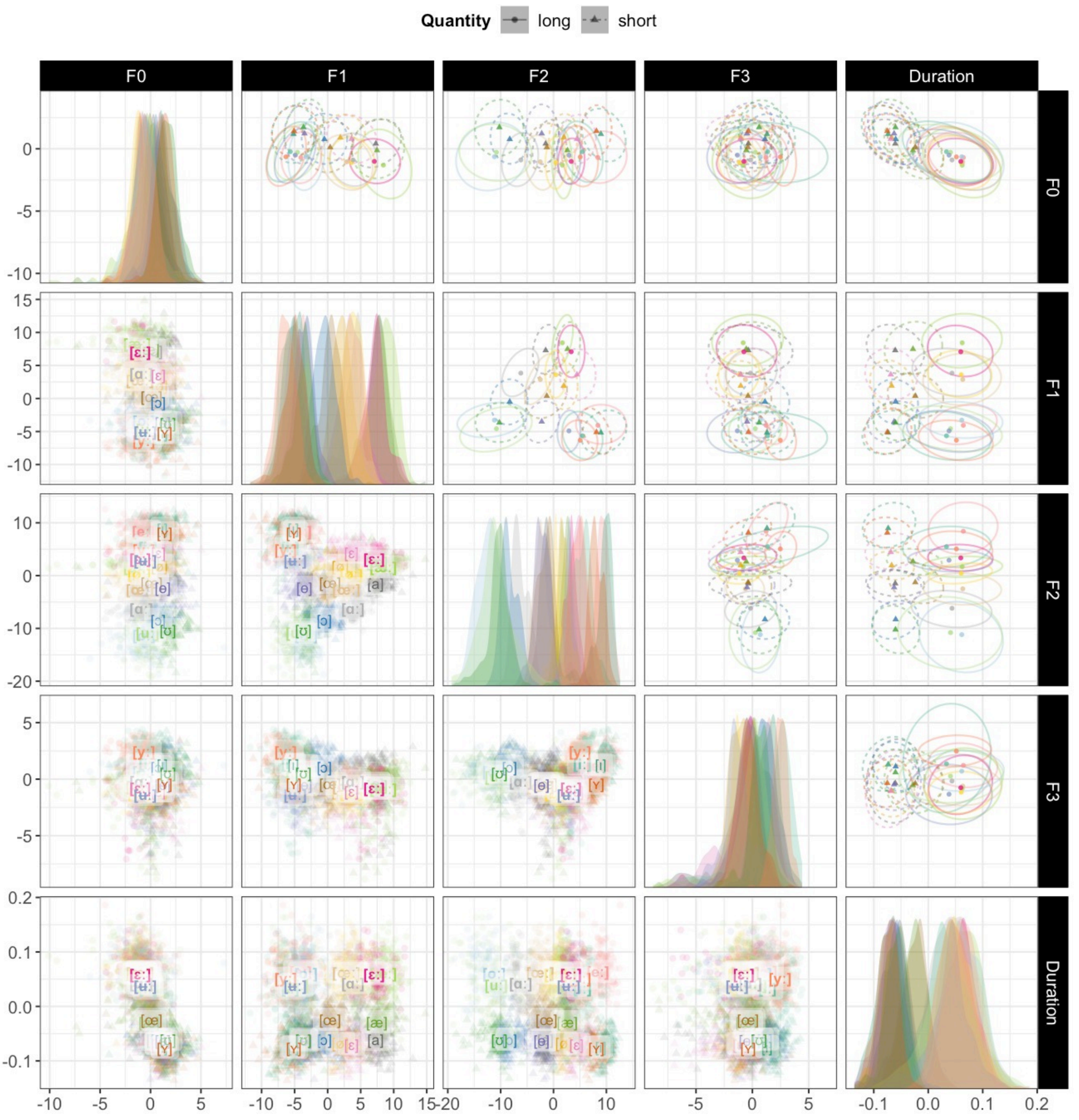

Figure S17: The SwehVd vowel data in C-CuRE semitones space. Points show repetitions of each of the 21 Central Swedish vowels by 16 female native talkers in the database in F0-F3 and vowel duration cue space. Vowel labels indicate category means across talkers. Long vowels are boldfaced. Ellipses show bivariate Gaussian 95% confidence interval of category means.

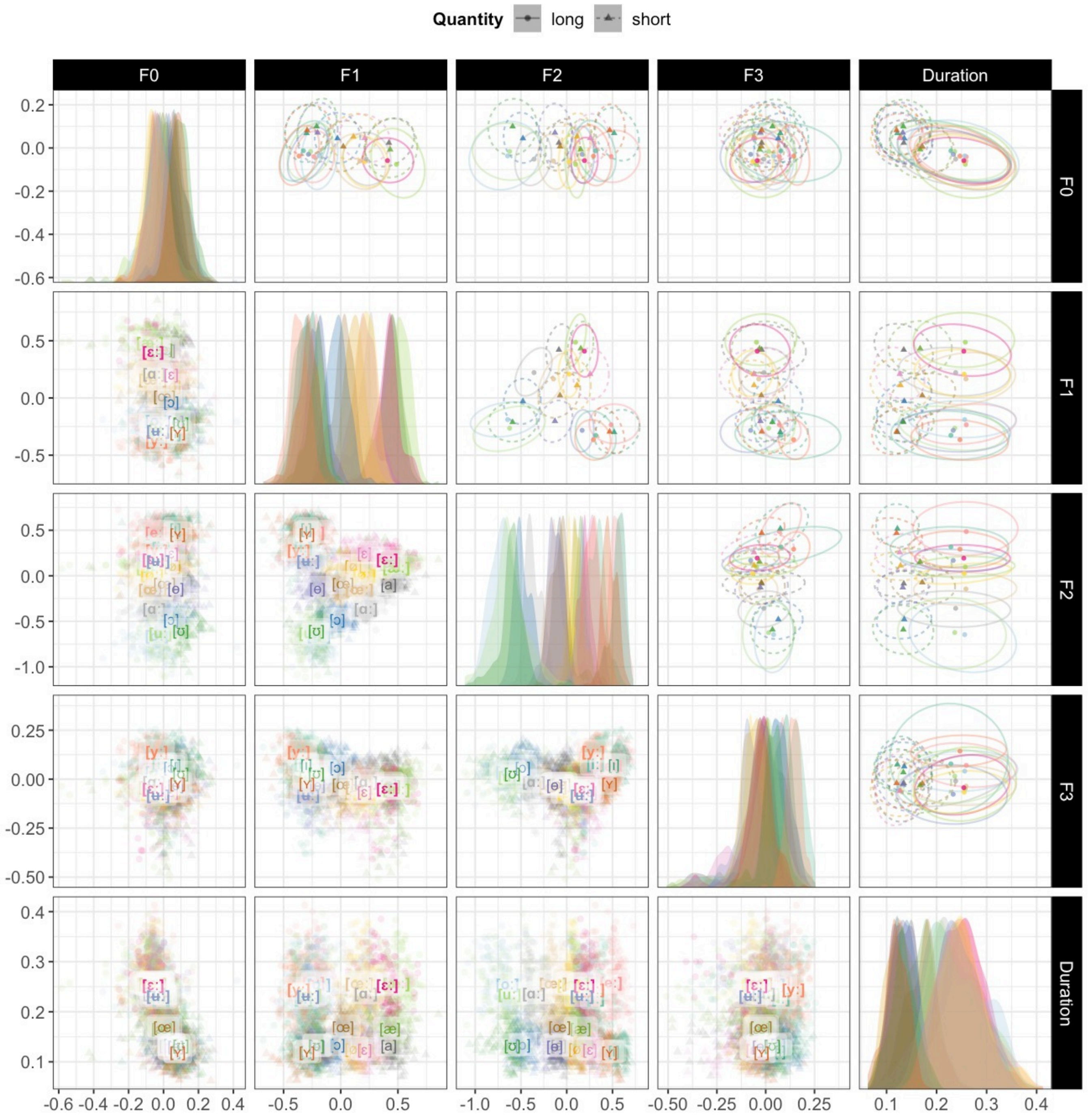

Figure S18: The SwehVd vowel data in Nearey1 (log) space. Points show repetitions of each of the 21 Central Swedish vowels by 16 female native talkers in the database in F0-F3 and vowel duration cue space. Vowel labels indicate category means across talkers. Long vowels are boldfaced. Ellipses show bivariate Gaussian 95% confidence interval of category means.

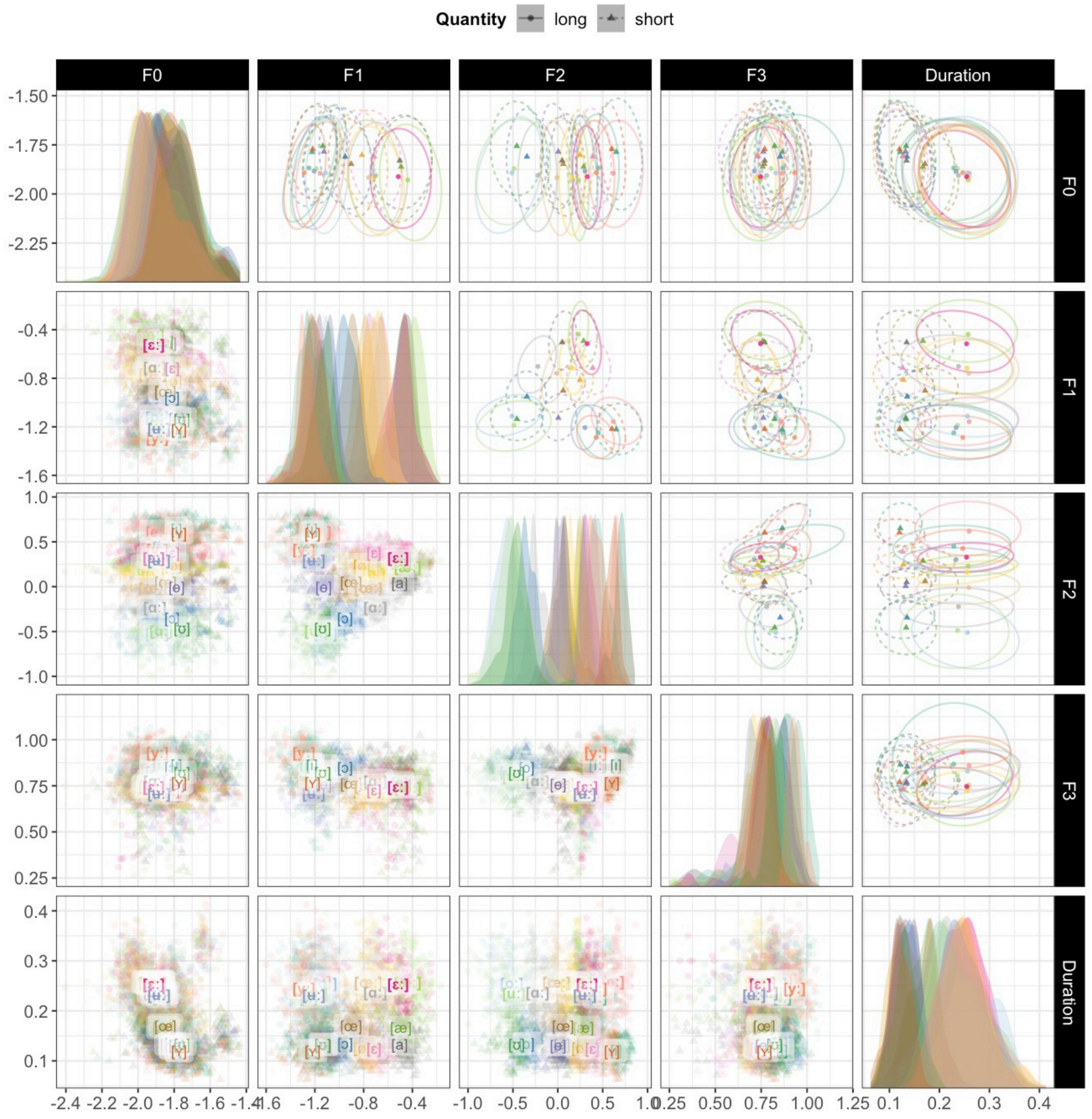

Figure S19: The SwehVd vowel data in Nearey2 (log) space. Points show repetitions of each of the 21 Central Swedish vowels by 16 female native talkers in the database in F0-F3 and vowel duration cue space. Vowel labels indicate category means across talkers. Long vowels are boldfaced. Ellipses show bivariate Gaussian 95% confidence interval of category means.

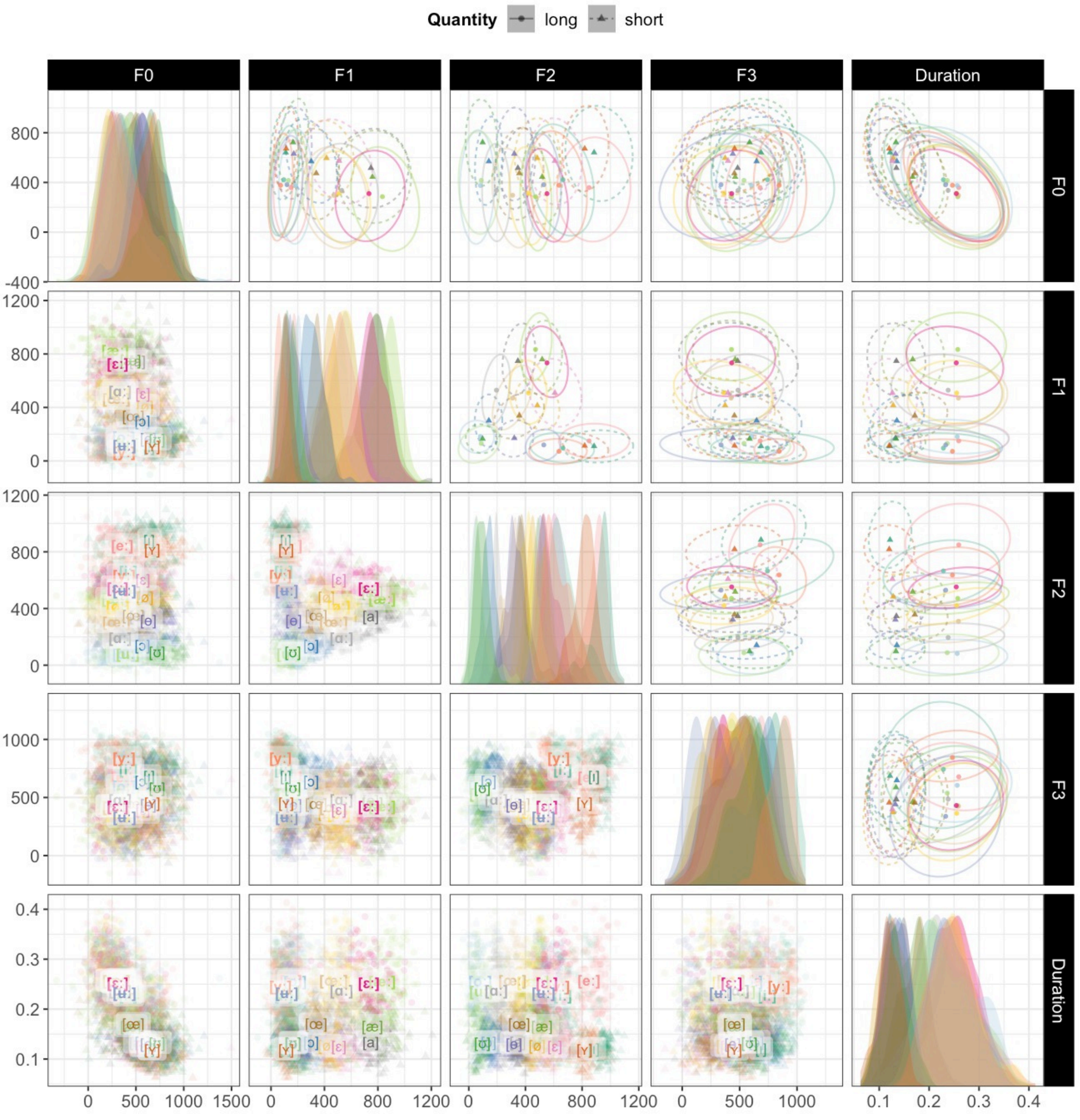

Figure S20: The SwehVd vowel data in Gerstman (Hz) space. Points show repetitions of each of the 21 Central Swedish vowels by 16 female native talkers in the database in F0-F3 and vowel duration cue space. Vowel labels indicate category means across talkers. Long vowels are boldfaced. Ellipses show bivariate Gaussian 95% confidence interval of category means.

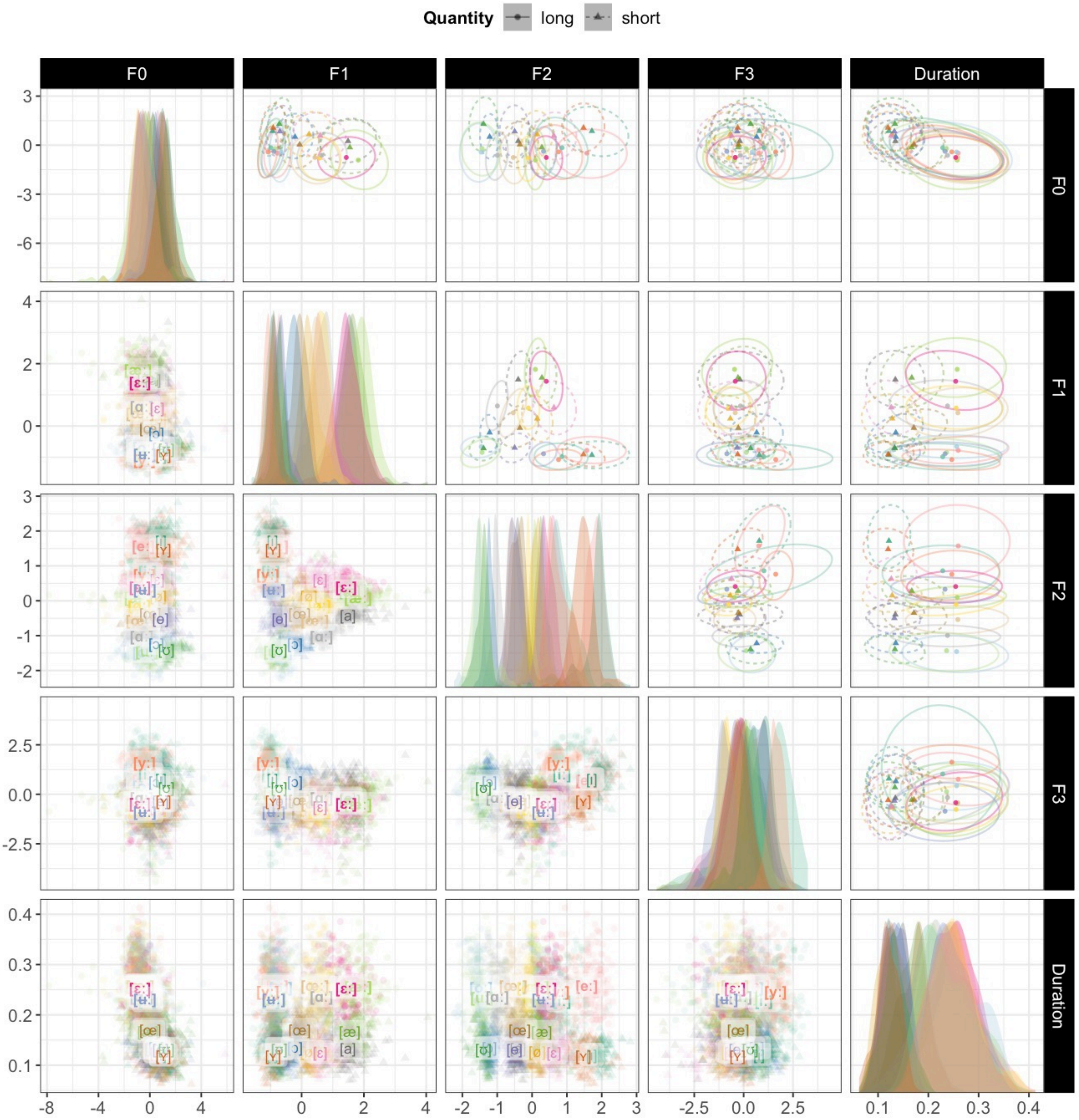

Figure S21: The SwehVd vowel data in Lobanov (Hz) space. Points show repetitions of each of the 21 Central Swedish vowels by 16 female native talkers in the database in F0-F3 and vowel duration cue space. Vowel labels indicate category means across talkers. Long vowels are boldfaced. Ellipses show bivariate Gaussian 95% confidence interval of category means.

6 VOWEL-SPECIFIC IDEAL OBSERVER ANALYSES

The use of a perceptual model (here: ideal observers) also makes it straightforward to assess vowel-specific effects of normalization. The next two subsections provide both the predicted categorization accuracy per vowel in the different evaluations, as well as confusion matrices of the best and the worst performing ideal observers, shedding light on *how* normalization improves recognition accuracy.

6.1 Per-vowel categorization accuracy of models trained on long and short vowels separately

Figures S22 - S26 show the vowel-specific accuracies of the different normalization accounts. Unsurprisingly, some vowels are recognized with higher accuracy than others—at least when uniform category priors are assumed, as we did here. This is a direct consequence of the position of the vowel in the acoustic-phonetic space, relative to neighboring vowels: the more neighboring vowels overlap with each other, the lower the accuracy with which they are recognized. Which vowels will benefit from normalization will thus naturally vary between languages, reflecting the language-specific properties of the vowel space. For instance, [iː] is often described as more easily recognized in previous work on other languages. This contrasts with our findings for Central Swedish: here, [iː] is part of the dense clustering of vowels along the height dimension and so has many close competitors. This highlights that recognition accuracy is due to the position of a vowel *relative* to its competitors (e.g., Peterson and Barney, 1952; Kuhl, 1991; Polka and Bohn, 2003), rather than its *absolute location* in the vowel space (e.g., [iː] being a peripheral vowel).

Also of interest is that not all vowels exhibit the benefit of normalization. In general, across evaluations, it seems to be vowels that are already recognized with high accuracy that does not benefit from normalization, which replicate previous studies that have included per-vowel accuracies (e.g., Adank, 2003; Syrdal and Gopal, 1986). For one vowel in particular, normalization can actually be detrimental to recognition. The accuracy of some normalized models is reduced compared to unnormalized models for [ʉː] when more cues than F1 and F2 are considered. Finally, while there are minor differences across vowels in the relative goodness of different normalizations, the models that perform overall best also perform best on each vowel (in line with Adank, 2003). This further demonstrates the plausibility of these normalization accounts for perception.

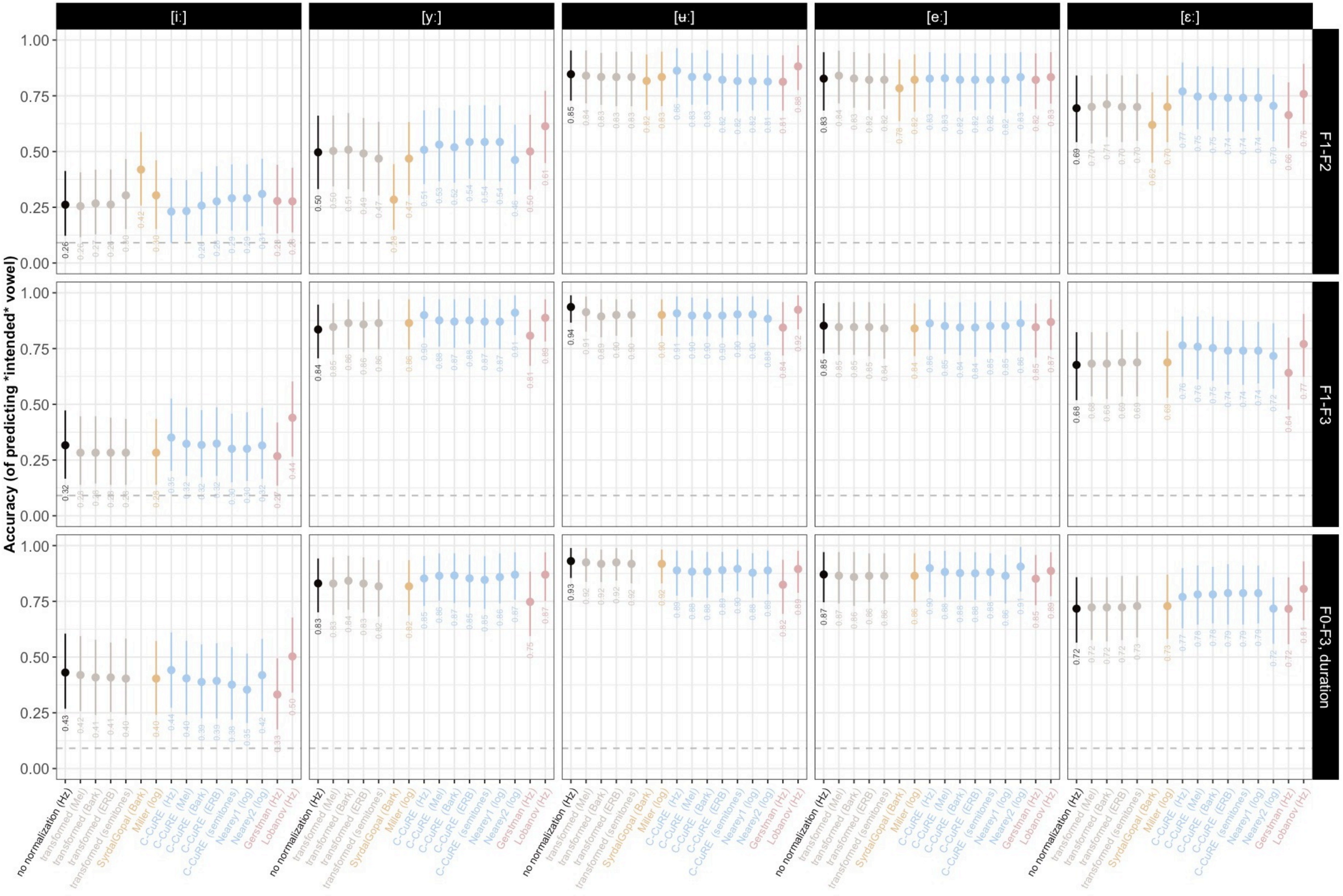

Figure S22: Per-vowel predicted categorization accuracy of the ideal observers trained on the **long** vowels, under different assumptions about the relevant cues. Point ranges indicate the average mean accuracy and average 95% bootstrapped CI across the five folds. Chance level is indicated by grey line.

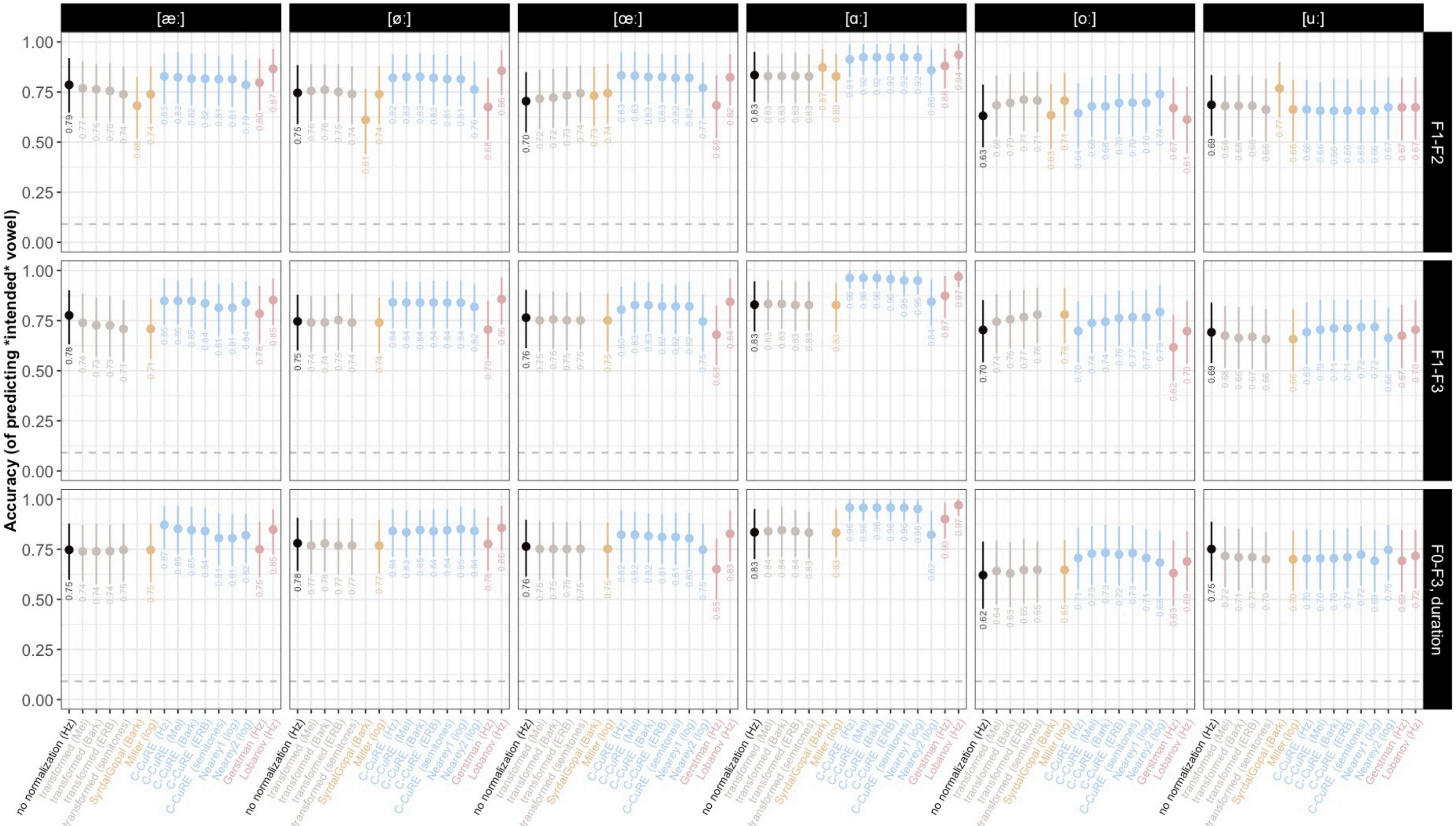

Figure S23: (*Continued from last page*)Per-vowel predicted categorization accuracy of the ideal observers trained on the **long** vowels, under different assumptions about the relevant cues. Point ranges indicate the average mean accuracy and average 95% bootstrapped CI across the five folds. Chance level is indicated by grey line.

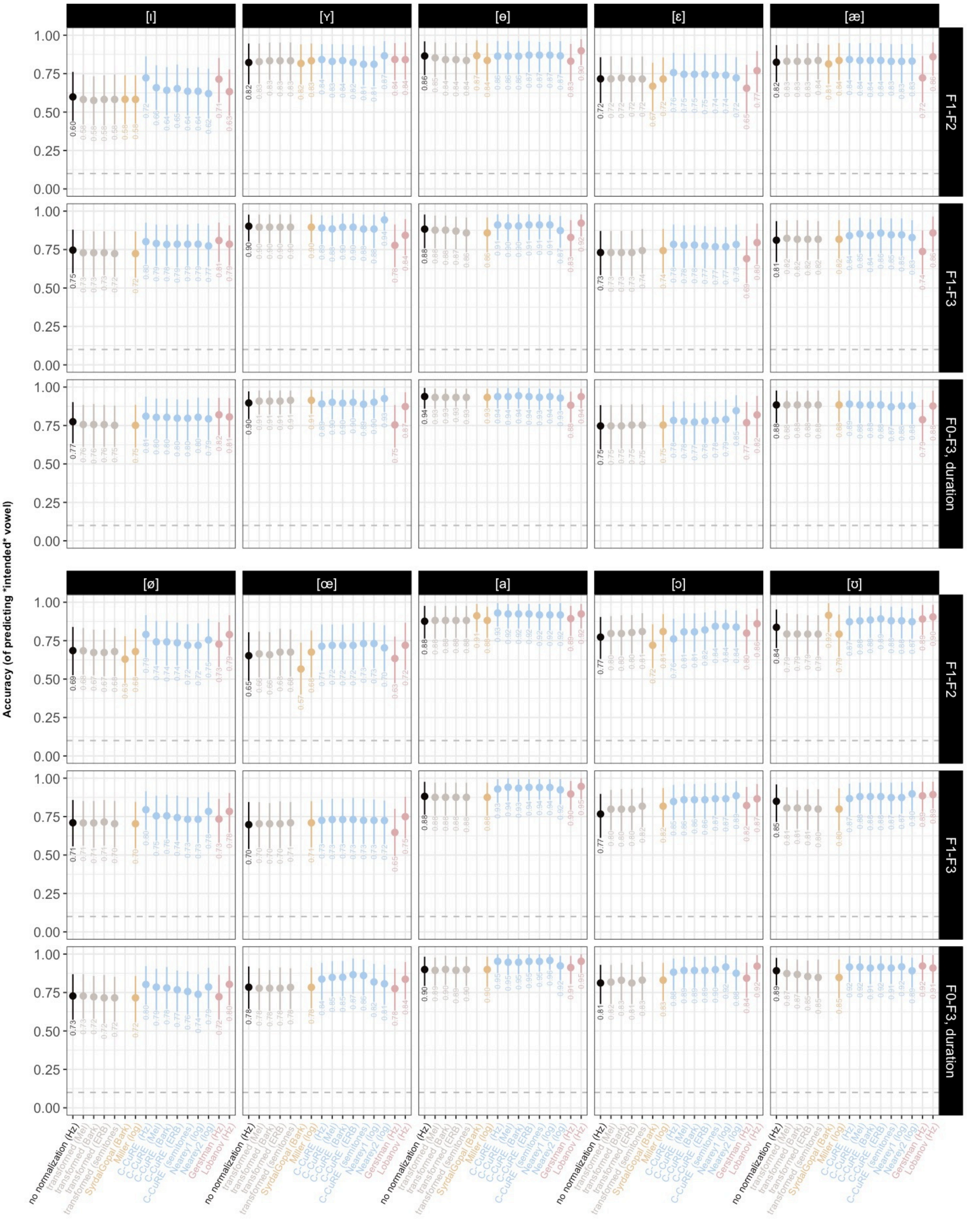

Figure S24: Per-vowel predicted categorization accuracy of the ideal observers trained on the **short** vowels, under different assumptions about the relevant cues. Point ranges indicate the average mean accuracy and average 95% bootstrapped CI across the five folds. Chance level is indicated by grey line.

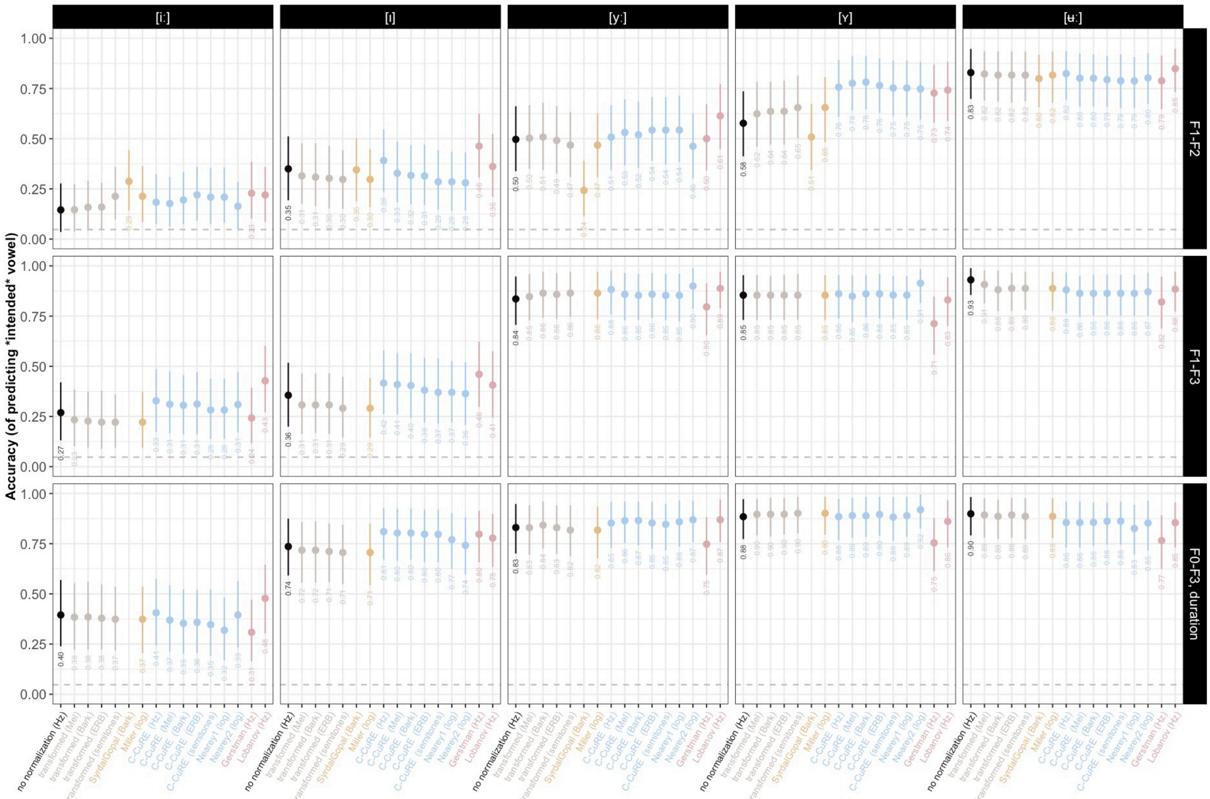

Figure S25: Per-vowel predicted categorization accuracy of the ideal observers trained on **all** vowels, under different assumptions about the relevant cues. Point ranges indicate the average mean accuracy and average 95% bootstrapped CI across the five folds. Chance level is indicated by grey line.

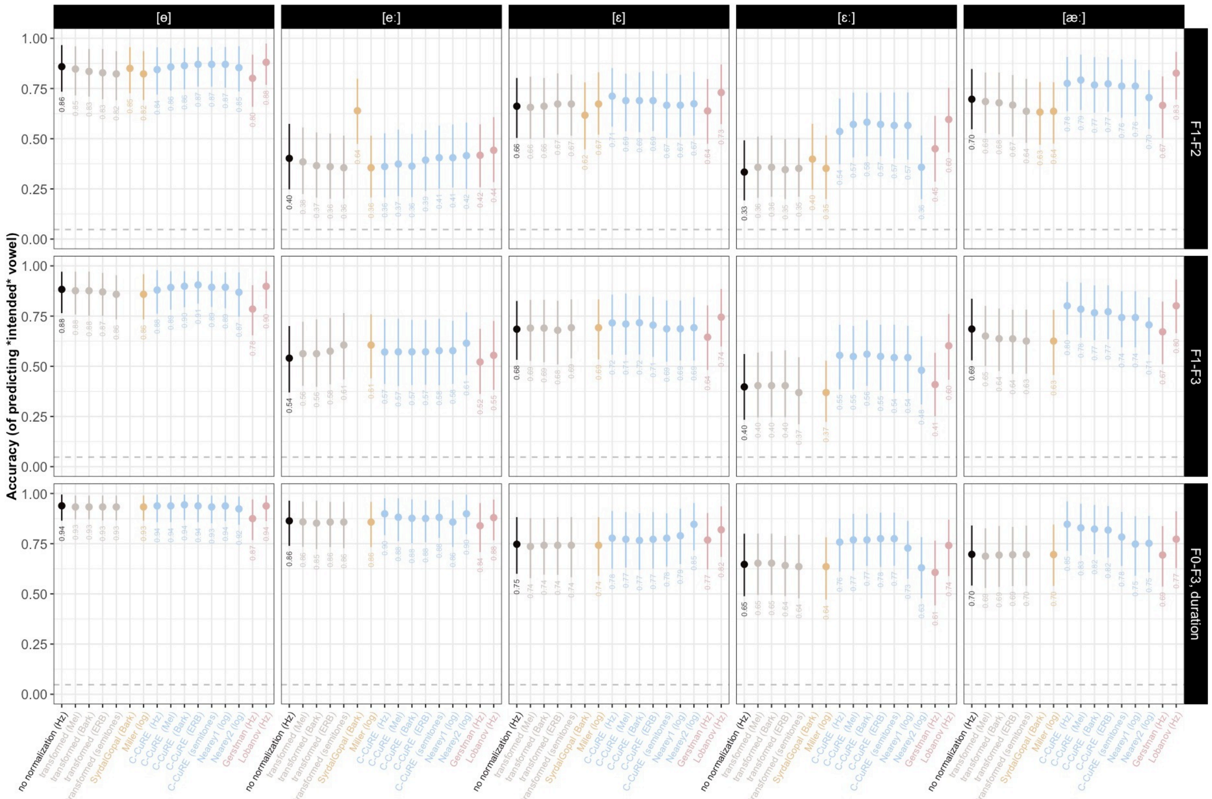

Figure S26: (*Continued from last page*)Per-vowel predicted categorization accuracy of the ideal observers trained on **all** vowels, under different assumptions about the relevant cues. Point ranges indicate the average mean accuracy and average 95% bootstrapped CI across the five folds. Chance level is indicated by grey line.

6.2 Vowel-to-vowel confusion matrices

To further explore effects of normalization on vowel categorization, we investigate how vowel-to-vowel confusion is affected by different normalizations. Figures S27 - S29 show confusion matrices of the worst and best performing models trained on the long, short or all Central Swedish vowels, under the different assumptions about the relevant cues. Next to the confusion matrices, we plot difference matrices to facilitate comparison.

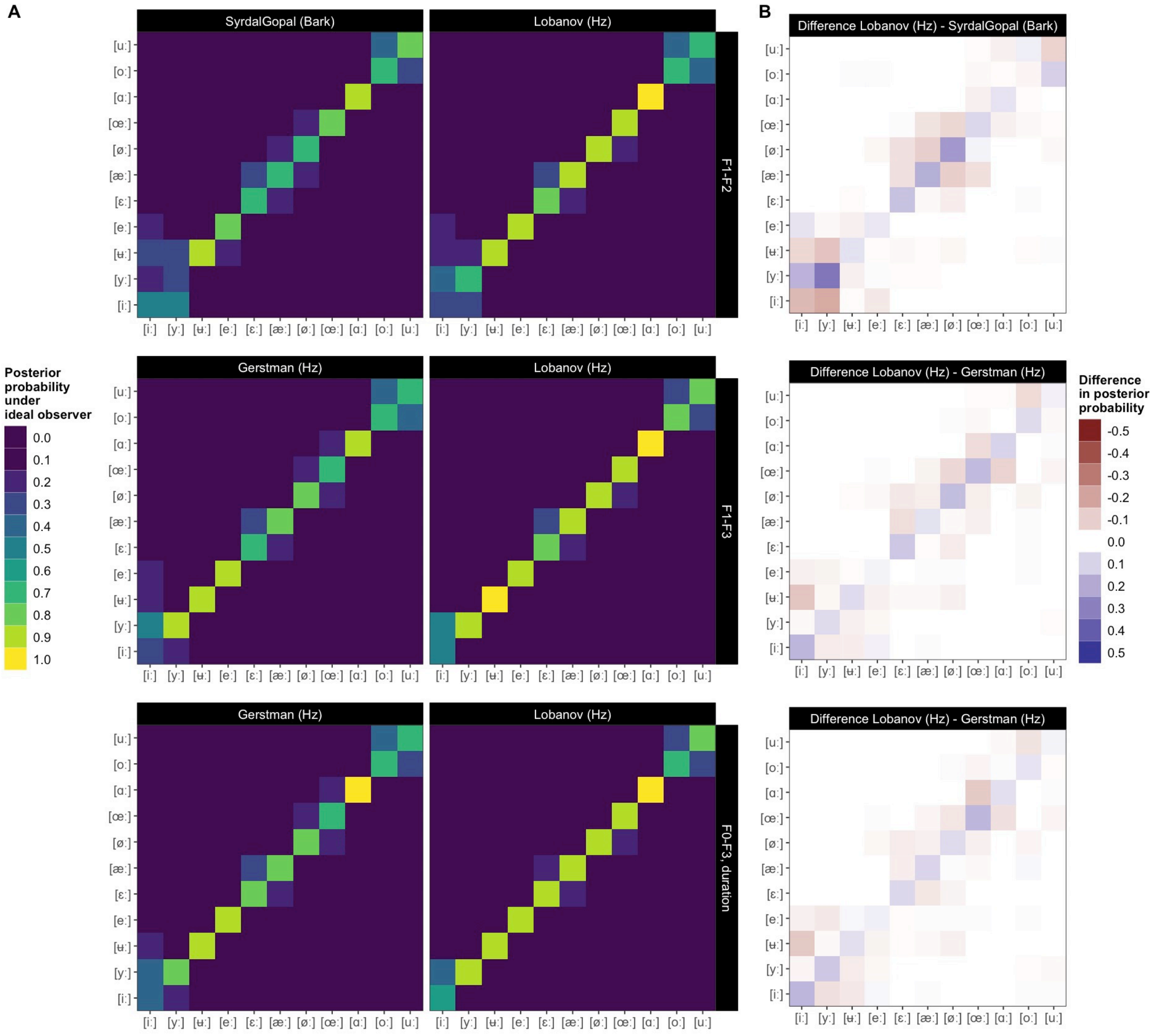

Figure S27: Illustration of the category-specific differences in predictions of the worst and best performing normalization models for each combination of cues (**rows**). The confusion matrices (**Panel A**) plot the predictions for the worst (**left**) and best (**right**) performing models in predicting the **long** vowels, under different assumptions about the relevant cues. Vowel intended by talker (x-axis) is plotted against vowel selected by ideal observer model (y-axis). Color fill indicates the posterior probability of the models predicting the intended vowel. The difference matrices (**Panel B**) illustrates the differences in predictions between the best and the worst performing models. Color fill indicates the difference in the posterior probability of the models predicting the intended vowel. More **purple** indicates an increase in posterior probability for the former over the latter model, more **red** indicates an advantage for the latter over the former.

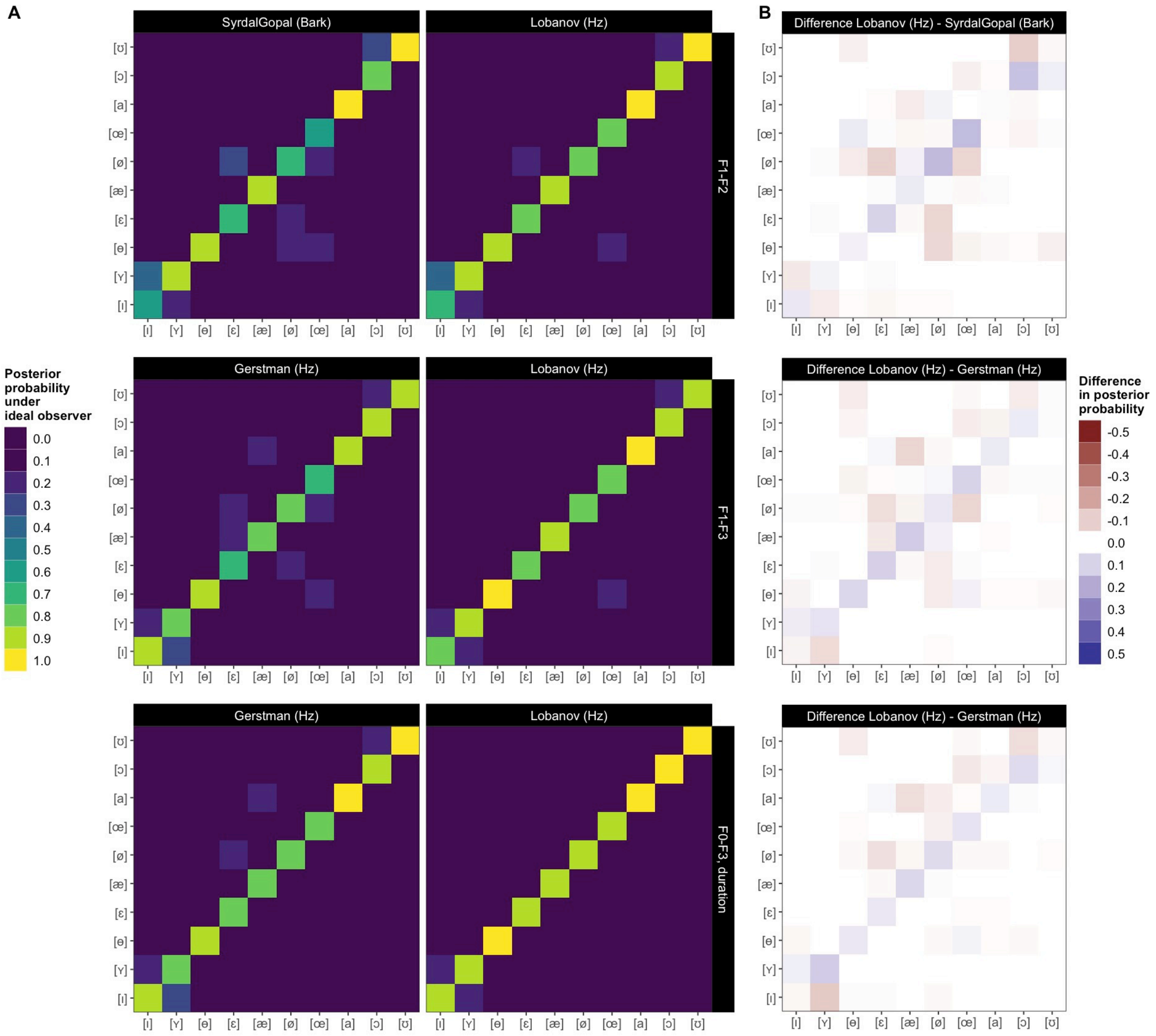

Figure S28: Illustration of the category-specific differences in predictions of the worst and best performing normalization models for each combination of cues (**rows**). The confusion matrices (**Panel A**) plot the predictions for the worst (**left**) and best (**right**) performing models in predicting the **short** vowels, under different assumptions about the relevant cues. Vowel intended by talker (x-axis) is plotted against vowel selected by ideal observer model (y-axis). Color fill indicates the posterior probability of the models predicting the intended vowel. The difference matrices (**Panel B**) illustrates the differences in predictions between the best and the worst performing models. Color fill indicates the difference in the posterior probability of the models predicting the intended vowel. More **purple** indicates an increase in posterior probability for the former over the latter model, more **red** indicates an advantage for the latter over the former.

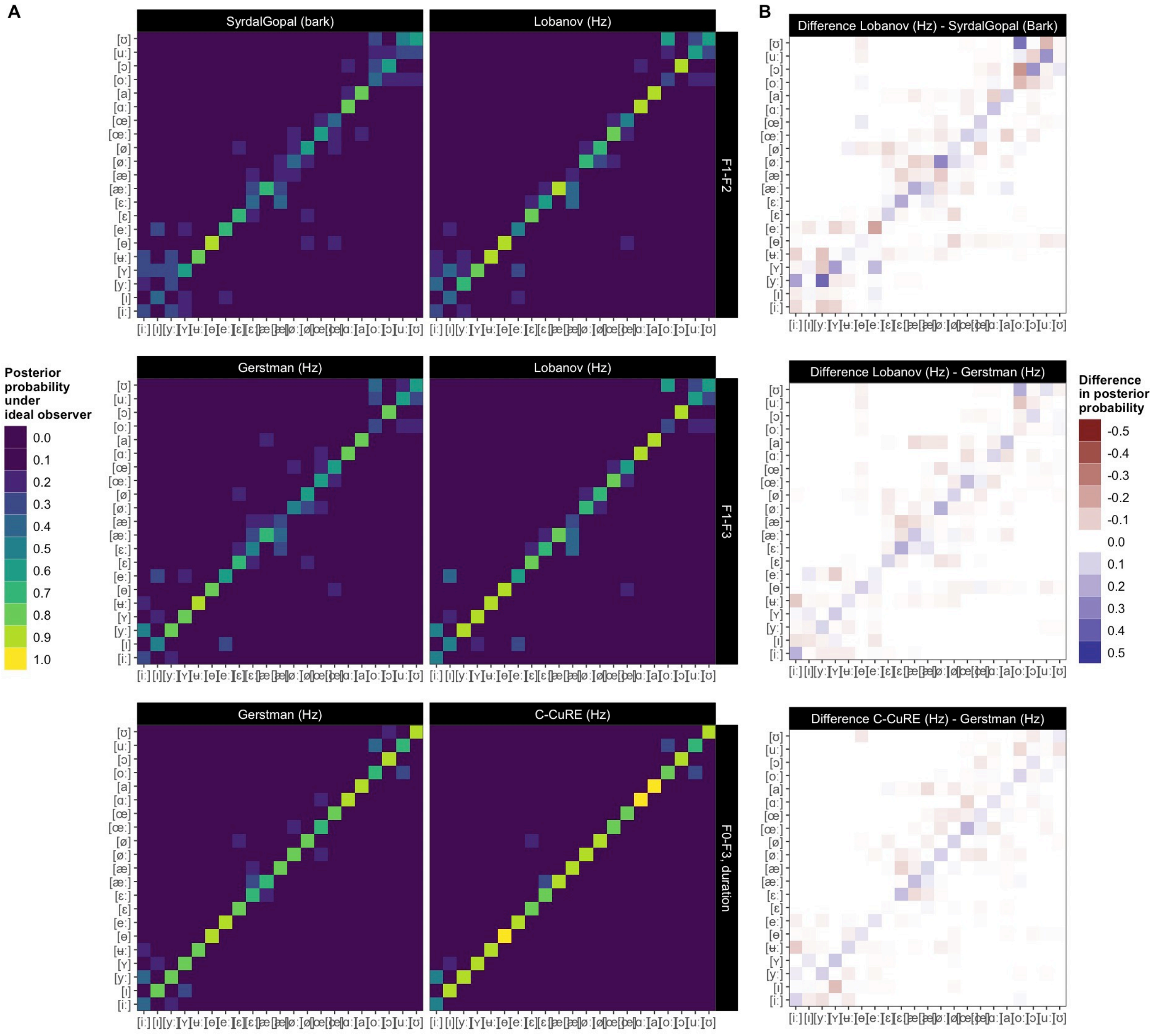

Figure S29: Illustration of the category-specific differences in predictions of the worst and best performing normalization models for each combination of cues (**rows**). The confusion matrices (**Panel A**) plot the predictions for the worst (**left**) and best (**right**) performing models in predicting the **all** vowels, under different assumptions about the relevant cues. Vowel intended by talker (x-axis) is plotted against vowel selected by ideal observer model (y-axis). Color fill indicates the posterior probability of the models predicting the intended vowel. The difference matrices (**Panel B**) illustrates the differences in predictions between the best and the worst performing models. Color fill indicates the difference in the posterior probability of the models predicting the intended vowel. More **purple** indicates an increase in posterior probability for the former over the latter model, more **red** indicates an advantage for the latter over the former.

7 AUXLIARY STUDY: COMPARING THE EFFECTS OF NORMALIZATION ACCOUNTS ON BETWEEN- VS. WITHIN-CATEGORY VARIABILITY

A large portion of previous studies evaluating normalization accounts against production data, has compared approaches in terms of how they affect category variability. In this additional study, we follow this traditional approach and evaluate how effectively different normalization accounts reduce the within-category variability of Central Swedish vowels. We calculate a separability index under different assumptions about the relevant cues and the size of the vowel space (the long and short vowels separately, or the entire space) and assess the effects on vowel category variability. To anticipate one take-home point, the results highlight important shortcomings of separability indices in evaluating normalization accounts and underlines the benefits of using a perceptual model to compare the effects of different normalization accounts.

Before we evaluate how category separability is affected by normalization in F1-F2, F1-F3, and F0-F3 and duration space, we look at how the normalization accounts affect the separability of vowels along each cue separately (Figure S30). As we show below, this is helpful in understanding the subsequently presented results for combinations of cues.

Table S2. Previous studies comparing the effectiveness of normalization accounts in reducing within-category cue variability

Language investigated	Article	Speech materials	Normalization accounts	Approach	Best two performing
US English	Barreda & Nearey, 2018	120,000 simulated languages (of 5 or 9 vowels) modeled on Hillenbrand et al.'s (1995) data (98 female/male child/adult talkers * 12 vowels)	Nearey2, Lobanov, log-mean in linear regression framework	distance between means (Eucledian distance)	log-mean in linear regression framework (1), Nearey2 (2)
	Clopper, 2009	2 female/male talkers from Ohio (1 token * 10 vowels)	Bladon et al.'s scale factor of 1 Bark (1994), Syrdal & Gopal, Nordström & Lindblom, Nearey1, Nearey2, Watt & Fabricius, Gerstman, Lobanov, Miller	variance reduction (visual inspection)	Nearey, Watt & Fabricius, Gerstman, Lobanov (no order)
	Hindle, 1978	Peterson & Barney's (1952) database; 19 female/male talkers from Philadelphia + 60 telephone informants (minimum 3 tokens per category; analysis focus on /ay/)	Nearey2, Nordström-Lindblom, Sankoff-Shorrock-McKay	distance between means, variance reduction (regression)	Sankoff (1)
	Kohn & Farrington, 2012	Longitudinal data from 10 female/male African American talkers from North Carolina (approx. 10 tokens * 10 vowels * 5 ages)	Lobanov, Gerstman, Nearey1, Nordström & Lindblom, Syrdal & Gopal/Thomas, Watt & Fabricius	variance reduction (regression)	Lobanov (1), Gerstman, Watt & Fabricius (2)
	Labov, 2010	Peterson & Barney's (1952) database; Philadelphia/Linguistic Change and Variation project (120 female/male talkers, stratified for age, sociolinguistic factors)	Nearey2, Nordström-Lindblom, Sankoff-Shorrock-McKay	distance between means (F-statistics)	Sankoff (1), Nearey2 (2)
US English, Norwegian, Swedish, German, Danish, Dutch	Disner, 1980	Differing number of tokens, vowels, and phonetic contexts across the six languages	Gerstman, Lobanov, Nearey2, Harshman's PARAFAC model	variance reduction (visual inspection)	Nearey2 (1), Lobanov (2)
UK English	Fabricius, Watt & Johnson, 2009	20 old/young female/male talkers of Received pronunciation (11 vowels); 6 old/young female/male talkers of Aberdeen English (8 vowels in different phonetic contexts)	Watt & Fabricius, Lobanov, Nearey1	variance reduction (SCV in talker-means)	Lobanov (1), Watt & Fabricius (2)
	Flynn & Foulkes, 2011	20 old/young female/male Nottingham talkers (mean 180 recordings per talker; categories not reported)	log-transformation (base 10), log-transformation (natural), Mel, ERB, Bark (*2 gender-specific versions), Syrdal & Gopal, Nordström (*2 gender-specific versions), LCE, Gerstman, Lobanov, Watt & Fabricius (* 4 versions), lettER, Nearey (*4 versions)		Gerstman (1), LCE (2)
Russian	Lobanov, 1971	5 female/male talkers (9 vowels in different phonetic contexts)	linear compression or expansion (Fant, 1960), Gerstman, Lobanov	distance between means	Lobanov (1), Gerstman (2)

7.1 Methods

7.1.1 Speech materials

This study employs the same speech materials as in the main study. Paralleling the main study, we evaluated category separability for each combination of normalization account, cues, and training-test fold. Specifically, we use the exact same cross-validation folds as in the main study.

7.1.2 Separability index

Previous studies have used different measures to assess the relative success of a normalization procedure in reducing inter-talker variability (see Table S2 and Nearey, 1989 for an overview on classification accounts). This includes assessing the reduction in variance or distance between means by visual inspection (e.g., Clopper, 2009; Disner, 1980; Hindle, 1978), or by calculating the reduction in within-category variance across talkers (e.g., Disner, 1980; Fabricius et al., 2009; Flynn and Foulkes, 2011; Hindle, 1978), or comparing the degree of separation between category means for unnormalized and normalized data, i.e., an F-ratio (e.g., Labov, 2010). We will assess how distinguishable vowels become under different normalization accounts by calculating a separability index, as described in Equation (S1). Following some previous studies (e.g., Labov, 2010), this separability index is essentially an F statistics, where the F statistics is the ratio of the within- and between-category variances:

$$
\begin{aligned}
separability\ index &= \frac{between\ category\ MS}{within\ category\ MS} \\
&= \frac{\sum\limits_{c=1,\ldots,K} (N_c - 1)}{K-1} \frac{\sum\limits_{c=1,\ldots,K} (\bar{x}_c - \bar{x})^2}{\sum\limits_{c=1,\ldots,K} \sum\limits_{i=1,\ldots,N_c} (x_{i,c} - \bar{x}_c)^2}
\end{aligned} \tag{S1}
$$

where K is the number of categories, N_c is the number of observations for category c, $x_{i,c}$ is the cue vector (for all cues considered in the calculation of the separability index) for observation i of category c, $\bar{x}_c$ is the cue mean vector for category c, and $\bar{x}$ is the overall cue mean vector. We calculated this separability index separately for each combination of normalization account, cues, and training-test fold, as described next.

7.2 Results

For F1 (first row of Figure S30), we see a clear advantage for centering (in blue) and standardizing (in purple) compared to transformations (in grey) and intrinsic accounts (in yellow). In particular Lobanov normalization seems to maximize category separability along F1, at least for the long vowels and all vowels together. Notably, the accounts pattern differently along F2 (second row of Figure S30). Overall, differences between accounts are much smaller along F2, and the clear advantage of centering and standardizing accounts along F1 does not extend to F2.

An altogether different picture is observed for F3. Compared to F1 and F2, the intrinsic account (Miller) performs substantially better in separating categories along F3, while all other accounts perform poorly. This result is surprising: one of the downsides of intrinsic approaches that has been noted in previous work is their sensitivity to measurement error (Thomas and Kendall, 2007). This

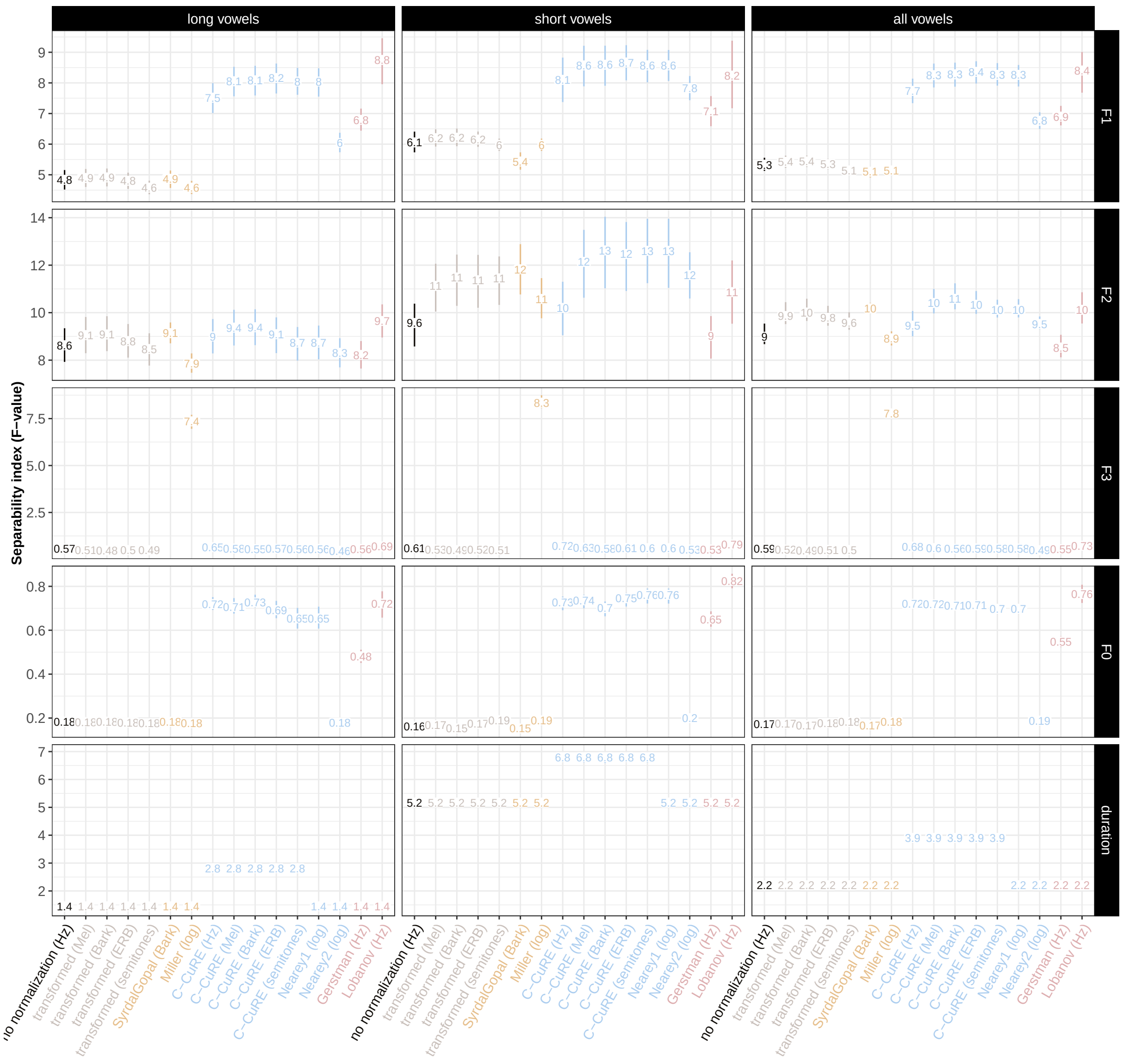

Figure S30: Separability indices by normalization accounts for long vowels, short vowels, and all vowels together (columns), shown for each of the five cues considered in this study (rows). Labels indicate mean across the five test folds. Intervals show average bootstrapped 95% confidence intervals across the test folds. Note that the ranges of the y-axes varies across plots.

sensitivity is caused by the fact that intrinsic accounts use a single measurement for normalization, rather than the less noisy estimates resulting from aggregating across segments that are used in extrinsic accounts. Since the third formant is often described as more difficult to reliably estimate than other formants (leading to more measurement error), F3 would be expected to be particularly affected by this weakness of intrinsic accounts.

Yet, further visualization in Figure S31 confirms that F3 indeed separates categories particularly well when intrinsic normalization is applied. Compared to other accounts, Miller (1989) seems to be particularly successful in separating vowels that differ in lip rounding. For example, Miller (1989) separates two clusters among the high and mid-high vowels, one consisting of the back vowels [oː] and [uː], and the other one of the front [iː], and rounded [yː] and [ʉː]. One possible explanation

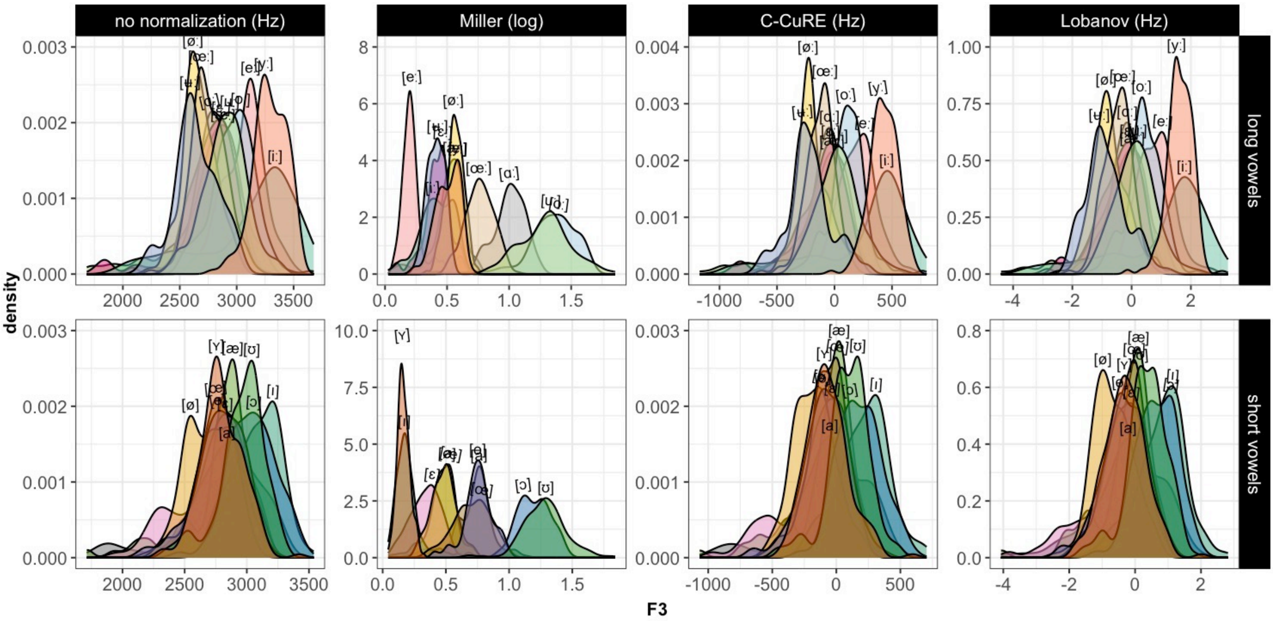

Figure S31: Category densities along F3 illustrates the effectiveness of vowel-intrinsic normalization for this cue. Here shown for Miller, compared to vowel-extrinsic accounts that center and/or standardize cues. For reference, densities in the absence of normalization are also shown.

for this result is that intrinsic normalization is indeed particularly effective for F3, and that our correction of measurement errors—equally applied to all formants—effectively reduced the issue with F3 measurement errors (presumably the human brain, too, can do better than an uncorrected Praat algorithm without error correction). As we show below, this result for F3 carries over to any combination of cues that includes F3. It is, however, an artifact of using category separability to assess the effectiveness of normalization, as we show in the main study. We elaborate on this issue in the discussion further down.

Returning to Figure S30, normalization does not increase category separability for F0. This is expected given that F0 is known to affect vowel separability primarily through its indirect influence on the interpretation of other formants (e.g., Barreda and Nearey, 2012; Barreda, 2020). Finally, for duration all of the C-CuRE accounts group together against the remaining accounts. This, too, is expected since all other accounts are formant-specific and thus do not normalize duration. In summary, the five cues contribute to category separability in different ways, and this is reflected in varying effectiveness of different normalization accounts. We also note that the best performing normalization account for any combination of cues and vowel qualities is typically never significantly better than the next best performing model (the 95% confidence intervals of the best model overlap with the mean of the next best model). In fact, for many combinations of cues and vowel qualities, many of the models perform similarly.

Next, we summarize how normalization affects category separability when combinations of the fives cues are considered. Figure S32 shows the separability index for the different normalization accounts for three different combinations of cues. For the first row of Figure S32, we followed most previous research in assessing category separability for the combination of F1 and F2 (e.g., Disner, 1980; Fabricius et al., 2009; Flynn and Foulkes, 2011; Hindle, 1978; Labov, 2010). Accounts that center against the talker’s overall formant mean (in blue) are among the best

performing normalization accounts. No matter the assumed perceptual scale, centering always improves category separability. Standardizing accounts (in purple), primarily Lobanov (1971), also perform well at separating categories, more so for the long vowels. However, scale transformations (in grey), and intrinsic accounts (in yellow), do not improve category separability compared to unnormalized Hz, at least not when assessed on the long vowels or the entire vowel space.

The remaining rows of Figure S32 compare normalization accounts when F3 (second row) or F0, F3, and duration are included (third row). Overall, the category separability is now lower, a result of how the accounts affect category separability along the cues added (see Figure S30). The most drastic change in performance concerns the intrinsic Miller (1989) and the standardizing accounts. When including F3, Miller (1989) performs as well or better, in absolute numbers, as when evaluated on only the combination of F1 and F2, thereby increasing its performance relative to other accounts. This increase in performance might be particularly pronounced for languages like Swedish, where F3 carries important information about lip rounding and thus vowel identity. In contrast, performance of standardizing accounts drops substantially if F3 or any other cue besides F1 and F2 is included.[S3] This mirrors what was found when assessing category separability separately for each cue (Figure S30).

Finally, looking across all three rows, category separability is consistently higher for short than long vowels. The same pattern is evident for each cue separately in Figure S30. This result conceptually replicates an initially surprising result of the main study: while short vowels are more densely clustered in the center of the vowel space, and thus occupy a smaller perceptual space, they also exhibit less category variability and less category overlap, making them overall more separable.

7.3 Discussion

When only F1 and F2 are considered, as in most previous work on vowel normalization, we find that extrinsic centering and standardizing accounts achieve the best category separability. Within these two types of accounts, there is considerable variability. For example, among the intrinsic accounts, Miller performs worse than Syrdal & Gopal, among the extrinsic accounts, versions of C-CuRE seem to consistently perform best. It is also worth noting, however, that there is never a single account that performs significantly better than all other normalizations. This points to the inherent similarities across normalization accounts, and perhaps limitations of the approach taken here (and in some previous work). This point is also raised in the general discussion in the main paper. Regardless of these caveats, the findings for F1 and F2 in this additional study, revise the results of Disner (1980) for Swedish, and instead replicates previous findings for the other Germanic languages in Disner's sample as well as the majority of previous studies on other languages (e.g., Fabricius et al., 2009; Flynn and Foulkes, 2011; Labov, 2010).

However, when F3 is considered along with F1 and F2, this result does no longer hold. Key to understanding this result and what it says about the suitability of category separability as a measure of normalization accounts is Figure S30: while extrinsic normalization performs better than other approaches for F1 and F2, the absolute differences in performance are small compared to the advantage of the intrinsic account observed for F3. Combined with a seemingly innocuous aspect of the separability index in Equation (S1), this allows separability along F3 to dominate separability along the other cue dimensions. Our separability index takes the *sum* of (squared)

[S3] We confirmed this by conducting additional comparisons using only F1, F2 and F0, or only F1, F2 and duration. For both of these comparisons too, we found that standardizing accounts perform poorly.

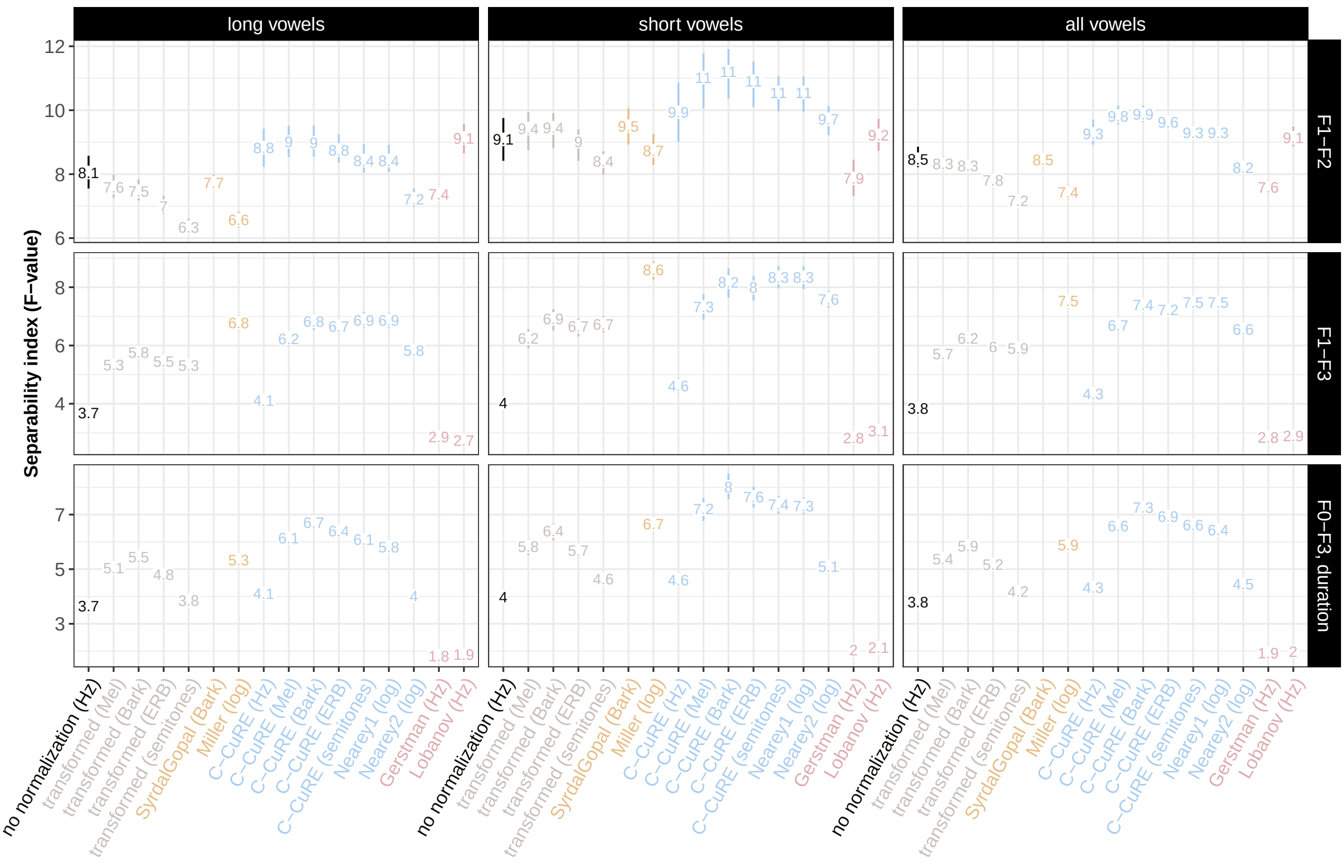

Figure S32: Separability indices by normalization accounts for long vowels, short vowels, and both long and short vowels together (columns) shown for three different combinations of cues (rows). Labels indicate mean across the five test folds. Intervals show average bootstrapped 95% confidence intervals across the test folds. Note that the ranges of the y-axes varies across plots.

distances along each cue dimension, essentially assuming that the effect of all cues is simply a sum of each cue's effect considered separately. This means that the separability index cannot capture the *joint* effect of cues—whether, for example, one cue effectively separates one set of categories and another cue separates another set of categories, rather than both cues separating the same categories. The separability index thus cannot recognize, for example, that F1 and F2 capture largely complementary aspects of the vowel inventory (as evident in, for example, Figures S5 and S6).

This is not the only deficiency of the separability index or similar measures of category variability. The use of *squared* distances means that even a small number of observations located far away from the category mean can disproportionally affect the index. Consider, for example, the F3 densities in Figure S31. For non-intrinsic normalizations, some categories have low but non-zero densities far away from the mode. Because of the use of squared distances, this results in low category separability for these normalization accounts despite the fact that observations with such cue values are rare and thus not expected to have a large effect on the *average* perceptual separability of vowels. For the same reason (the use of squared distances), category separability can be high even if a cue separates only a small subset of categories (as is the case for F3), compared to cues that more gradiently separates *all* categories (F1 and F2; see Figures S7 to S21).

In sum, indices of variability and category separability like that in Equation (S1) fail to adequately assess the expected consequences of normalization for perception, which is the primary interest of this paper, and addressed by the methodology we employed in the main study.

REFERENCES

Adank, P. (2003). *Vowel normalization: A perceptual-acoustic study of Dutch vowels.* Wageningen: Ponsen & Looijen.

Aust, F., and Barth, M. (2020). *papaja: Create APA manuscripts with R Markdown.* Available at: `https://github.com/crsh/papaja`.

Bache, S. M., and Wickham, H. (2020). *Magrittr: A forward-pipe operator for r.* Available at: `https://CRAN.R-project.org/package=magrittr`.

Barreda, S. (2020). Vowel normalization as perceptual constancy. *Language* 96, 224–254. doi:10.1353/lan.2020.0018.

Barreda, S., and Nearey, T. M. (2012). The direct and indirect roles of fundamental frequency in vowel perception. *The Journal of the Acoustical Society of America* 131, 466–477. doi:10.1121/1.3662068.

Barth, M. (2022). *tinylabels: Lightweight variable labels.* Available at: `https://cran.r-project.org/package=tinylabels`.

Bates, D., Mächler, M., Bolker, B., and Walker, S. (2015). Fitting linear mixed-effects models using lme4. *Journal of Statistical Software* 67, 1–48. doi:10.18637/jss.v067.i01.

Bates, D., and Maechler, M. (2021). *Matrix: Sparse and dense matrix classes and methods.* Available at: `https://CRAN.R-project.org/package=Matrix`.

Bürkner, P.-C. (2018). Advanced Bayesian multilevel modeling with the R package brms. *The R Journal* 10, 395–411. doi:10.32614/RJ-2018-017.

Bürkner, P.-C. (2021). Bayesian item response modeling in R with brms and Stan. *Journal of Statistical Software* 100, 1–54. doi:10.18637/jss.v100.i05.

Bürkner, P.-C. (2017). brms: An R package for Bayesian multilevel models using Stan. *Journal of Statistical Software* 80, 1–28. doi:10.18637/jss.v080.i01.

Clopper, C. G. (2009). Computational Methods for Normalizing Acoustic Vowel Data for Talker Differences: Computational Methods for Normalizing Acoustic Vowel Data. *Language and Linguistics Compass* 3, 1430–1442. doi:10.1111/j.1749-818X.2009.00165.x.

Csárdi, G., and Chang, W. (2021). *Processx: Execute and control system processes.* Available at: `https://CRAN.R-project.org/package=processx`.

Disner, S. F. (1980). Evaluation of vowel normalization procedures. *The Journal of the Acoustical Society of America* 67, 253–261. doi:10.1121/1.383734.

Dowle, M., and Srinivasan, A. (2021). *Data.table: Extension of 'data.frame'.* Available at: `https://CRAN.R-project.org/package=data.table`.

Eddelbuettel, D., and Balamuta, J. J. (2018). Extending extitR with extitC++: A Brief Introduction to extitRcpp. *The American Statistician* 72, 28–36. doi:10.1080/00031305.2017.1375990.

Eddelbuettel, D., and François, R. (2011). Rcpp: Seamless R and C++ integration. *Journal of Statistical Software* 40, 1–18. doi:10.18637/jss.v040.i08.

Fabricius, A., Watt, D., and Johnson, D. E. (2009). A comparison of three speaker-intrinsic vowel formant frequency normalization algorithms for sociophonetics. *Language Variation and Change* 21, 413–435. doi:10.1017/S0954394509990160.

Fant, G. (1983). Feature analysis of Swedish vowels - a revisit. *STL-QPSR* 24, 001–019.

Flynn, N., and Foulkes, P. (2011). Comparing vowel formant normalization methods. *Proceedings of ICPhS XVII*, 683–686.

Henry, L., and Wickham, H. (2020). *Purrr: Functional programming tools.* Available at: `https://CRAN.R-project.org/package=purrr`.

Henry, L., and Wickham, H. (2021). *Rlang: Functions for base types and core r and 'tidyverse' features.* Available at: `https://CRAN.R-project.org/package=rlang`.

Hindle, D. (1978). “Approaches to Vowel Normalization in the Study of Natural Speech,” in *Linguistic variation: Models and methods*, ed. D. Sankoff (New York: Academic Press), 161–171.

Hugh-Jones, D. (2021). *Latexdiffr: Diff 'rmarkdown' files using the 'latexdiff' utility.* Available at: `https://CRAN.R-project.org/package=latexdiffr`.

Kleinschmidt, D. F., and Jaeger, T. F. (2015b). Robust speech perception: Recognize the familiar, generalize to the similar, and adapt to the novel. *Psychological Review* 122, 148–203. Available at: `http://dx.doi.org/10.1037/a0038695`.

Kuhl, P. K. (1991). Human adults and human infants show a ”perceptual magnet effect” for the prototypes of speech categories, monkeys do not. *Perception & psychophysics* 50, 93–107. doi:10.3758/BF03212211.

Labov, W. (2010). *Principles of linguistic change. 2: Social factors.* repr. Chichester: Wiley-Blackwell.

Liao, Y. (2019). *Linguisticsdown: Easy linguistics document writing with r markdown.* Available at: `https://CRAN.R-project.org/package=linguisticsdown`.

Lobanov, B. M. (1971). Classification of Russian vowels spoken by different speakers. *The Journal of the Acoustical Society of America* 49, 606–608. doi:10.1121/1.1912396.

Maechler, M. (2021). *Diptest: Hartigan's dip test statistic for unimodality - corrected.* Available at: `https://CRAN.R-project.org/package=diptest`.

Müller, K., and Wickham, H. (2021). *Tibble: Simple data frames.* Available at: `https://CRAN.R-project.org/package=tibble`.

McMurray, B., and Jongman, A. (2011). What information is necessary for speech categorization?: Harnessing variability in the speech signal by integrating cues computed relative to expectations. *Psychological Review* 118, 219–246. doi:10.1037/a0022325.What.

Miller, J. D. (1989). Auditory-perceptual interpretation of the vowel. *The Journal of Acoustical Society of America* 85, 22.

Nearey, T. M. (1978). *Phonetic Feature Systems for Vowels.* Indiana.

Nearey, T. M. (1989). Static, dynamic, and relational properties in vowel perception. *The Journal of the Acoustical Society of America* 85, 2088–2113. doi:10.1121/1.397861.

Ooms, J. (2021). *Magick: Advanced graphics and image-processing in r.* Available at: `https://CRAN.R-project.org/package=magick`.

Pedersen, T. L., and Robinson, D. (2020). *Gganimate: A grammar of animated graphics.* Available at: `https://CRAN.R-project.org/package=gganimate`.

Peterson, G. E., and Barney, H. L. (1952). Control methods used in a study of the vowels. *Journal of the Acoustical Society of America* 24, 175–184.

Polka, L., and Bohn, O.-S. (2003). Asymmetries in vowel perception. *Speech Communication* 41, 221–231.

R Core Team (2021a). *R: A language and environment for statistical computing.* Vienna, Austria: R Foundation for Statistical Computing.

R Core Team (2021b). *R: A language and environment for statistical computing.* Vienna, Austria: R Foundation for Statistical Computing Available at: `https://www.R-project.org/`.

Riad, T. (2014). *The phonology of Swedish.* Oxford: Oxford University Press.

RStudio Team (2020). *RStudio: Integrated development environment for R.* Boston, MA: RStudio, PBC.

Sievert, C. (2020). *Interactive web-based data visualization with r, plotly, and shiny.* Chapman; Hall/CRC Available at: `https://plotly-r.com`.

Statisticat, and LLC. (2021). *LaplacesDemon: Complete environment for bayesian inference.* Bayesian-Inference.com Available at: `https://web.archive.org/web/20150206004624/http://www.bayesian-inference.com/software`.

Syrdal, A. K., and Gopal, H. S. (1986). A perceptual model of vowel recognition based on the auditory representation of American English vowels. *The Journal of the Acoustical Society of America* 79, 1086–1100. doi:10.1121/1.393381.

Thomas, E. R., and Kendall, T. (2007). NORM: The vowel normalization and plotting suite.

Wickham, H. (2019a). *Assertthat: Easy pre and post assertions.* Available at: `https://CRAN.R-project.org/package=assertthat`.

Wickham, H. (2021a). *Forcats: Tools for working with categorical variables (factors).* Available at: `https://CRAN.R-project.org/package=forcats`.

Wickham, H. (2016). *ggplot2: Elegant graphics for data analysis.* Springer-Verlag New York Available at: `https://ggplot2.tidyverse.org`.

Wickham, H. (2020). *Modelr: Modelling functions that work with the pipe.* Available at: `https://CRAN.R-project.org/package=modelr`.

Wickham, H. (2019b). *Stringr: Simple, consistent wrappers for common string operations.* Available at: `https://CRAN.R-project.org/package=stringr`.

Wickham, H. (2021b). *Tidyr: Tidy messy data.* Available at: `https://CRAN.R-project.org/package=tidyr`.

Wickham, H., Averick, M., Bryan, J., Chang, W., McGowan, L. D., François, R., Grolemund, G., Hayes, A., Henry, L., Hester, J., et al. (2019). Welcome to the tidyverse. *Journal of Open Source Software* 4, 1686. doi:10.21105/joss.01686.

Wickham, H., François, R., Henry, L., and Müller, K. (2021a). *Dplyr: A grammar of data manipulation.* Available at: `https://CRAN.R-project.org/package=dplyr`.

Wickham, H., Hester, J., and Bryan, J. (2021b). *Readr: Read rectangular text data.* Available at: `https://CRAN.R-project.org/package=readr`.

Wilke, C. O. (2020). *Cowplot: Streamlined plot theme and plot annotations for 'ggplot2'.* Available at: `https://CRAN.R-project.org/package=cowplot`.

Xie, Y., and Allaire, J. (2022). *Tufte: Tufte's styles for r markdown documents.* Available at: `https://CRAN.R-project.org/package=tufte`.
